# Integrative systematics and ecology of a new deep-sea family of tanaidacean crustaceans

**DOI:** 10.1038/s41598-019-53446-1

**Published:** 2019-12-10

**Authors:** Magdalena Błażewicz, Piotr Jóźwiak, Robert M. Jennings, Maciej Studzian, Inmaculada Frutos

**Affiliations:** 1 University of Lodz, Department of Invertebrate Zoology and Hydrobiology, Laboratory of Polar Biology and Oceanobiology, Banacha St. 12/16, Łódź, 90-237 Poland; 2 Biology Department, Temple University, Philadelphia, PA USA; 3 University of Lodz, Department of Molecular Biophysics, Banacha St. 12/16, Łódź, 90-237 Poland; 4 University of Hamburg, Centre of Natural History, Zoological Museum, Martin-Luther-King-Platz 3, 20146 Hamburg, Germany; 5 Universidad de Alcalá, Dpto. Ciencias de la Vida, EU-US Marine Biodiversity Group, 28871 Alcalá de Henares, Spain

**Keywords:** Zoology, Marine biology

## Abstract

A new family of paratanaoidean Tanaidacea – Paranarthrurellidae fam. nov. – is erected to accommodate two genera without family classification (Paratanaoidea *incertae sedis*), namely *Armatognathia* Kudinova-Pasternak, 1987 and *Paranarthrurella* Lang, 1971. Seven new species of *Paranarthrurella* and two of *Armatognathia* are described from material taken in different deep-sea areas of the Atlantic and Pacific oceans. The type species of *Paranarthrurella* — *P. caudata* (Kudinova-Pasternak, 1965) — is redescribed based on the paratype. The genus *Cheliasetosatanais* Larsen and Araújo-Silva, 2014 originally classified within Colletteidae is synonymised with *Paranarthrurella*, and *Arthrura shiinoi* Kudinova-Pasternak, 1973 is transferred to *Armatognathia*. Amended diagnoses of *Armatognathia* and *Paranarthrurella* genera are given. Choosing characters for distinguishing and defining both genera was supported by Principal Component Analysis. Designation of the new family is supported by molecular phylogenetic analysis of COI and 18S datasets. The distribution of all species currently included in the new family was visualised and their bathymetric distribution analysed.

## Introduction

The tanaidacean tanaidomorph genus *Paranarthrurella* was established by Lang (1971)^1^ to accommodate a conspicuous species described by Kudinova-Pasternak in 1965^2^ — *Leptognathia caudata* Kudinova-Pasternak, 1965. This genus reveals a tangled history. A special ‘general appearance’ expressed by robust cheliped, rounded (swollen) pleon and peculiar mouthparts was often underlined^1–4^ and although all specialists recognized the morphological distinctiveness of the material studied by them, there was no consensus on its systematic position. In consequence, four currently known nominal species of the genus *Paranarthrurella* (*P. arctophylax* (Norman and Stebbing, 1886), *P. caudata* (Kudinova-Pasternak, 1965), *P. dissimilis* (Lang, 1972) and *P. voeringi* (Sars, 1877)), have historically been classified primarily to six distinct genera, namely: *Tanais* Latreille, 1831^5,6^, *Cryptocope* Sars, 1882^7,8^, *Leptognathia* Hansen, 1913^1,3,9–11^, *Strongylura* (synonymized with *Collettea*)^12^, *Biarticulata* (=*Leptognathia*)^13^, and monotypic *Robustognathia* (=*Paranarthrurella*)^4^.

Equally confused was the systematic position of genus *Paranarthrurella*. Considering the width of the pleonite segments, which are narrower than the last pereonite or pleotelson in the females, Lang classified *P. caudata* in the Anarthruridae Lang, 1971 disregarding the connection of the cheliped to the body and the character of the exopod in the uropods^1^. Although the morphological uniqueness of *Paranarthrurella* was later mentioned by Sieg^14^, the genus was retained within the tribe Anarthurini in Anarthruridae. In the morphological phylogenetic approach of Larsen and Wilson^15^, *Paranarthrurella* was transferred to Agathotanaidae.

The revision of *Paranarthrurella*, grounded in revived *Paranarthrurella voeringi* (=*Tanais voeringi* (G.O. Sars, 1877)), has confirmed that this genus, with the cheliped attached to the body *via* a sclerite, cannot be a member of Anarthruridae or Agathotanaidae^16^. It was also emphasised that a unique character of the chelipeds and the mouthparts is distinct among all currently defined families of Tanaidomorpha. Subsequently, *Paranarthrurella* increased the number of genera of Paratanaoidean family ‘*incertae sedis*’.

*Armatognathia*, described by Kudinova-Pasternak (1987)^17^ from the Indian Ocean, represents another monotypic genus that remains unclassified to any of the existing tanaidacean families^15^. The type species, *A. birsteini* Kudinova-Pasternak, 1987, and the genus were originally placed within the family Leptognathiidae^17^, where it was retained until the phylogenetic analysis of Larsen and Wilson^15^. Kudinova-Pasternak (*op. cit*.) pointed out that the main character differentiating *Armatognathia* from the remaining leptognathiid genera was the armament of the mandible molar process. According to the author, *Armatognathia* shows some affinities with *Leptognathia*, but differs in the presence of distal spines on the mandibular molar, nine terminal spines on the maxillule palp, and restriction of setation of the pleopods rami to the distal margin.

During examination of the Tanaidacea from various collections made in different deep-sea parts of the Atlantic and the Pacific oceans, we have revealed a series of distinct species, which we classified to two genera: *Paranarthrurella* and *Armatognathia*. In this paper we (1) describe all the new species, (2) rediagnose both genera, (3) discuss their relationship, and (4) assign them to a new family Paranarthrurellidae. Moreover, we synonymize the genus *Cheliasetosatanais* Larsen and Araújo-Silva, 2014^18^, considered a member of the family Colletteidae, with *Paranarthrurella*, and we transfer the one tanaellid species *Arthrura shiinoi* Kudinova-Pasternak, 1973 to the genus *Armatognathia*. Similar systematic rearrangement was suggested by Bird and Holdich^19^. Our morphological analyses are supported by molecular phylogenetic analysis of mitochondrial COI and nuclear 18S molecular markers. Also, where possible, we use environmental data (as temperature and salinity) to assess distribution of the genus *Paranarthrurella* in the North Atlantic. Finally, we visualize and analyse the zoogeographic and bathymetric distribution of all species belonging to this new family. An identification key to females of the known twelve species of *Paranarthrurella* is also provided.

## Material

The specimens used in this study were collected during different scientific initiatives leaded by various research expeditions and teams. The vast majority of specimens used for species descriptions were loaned from Museum of Comparative Zoology (Harvard University) as the result of the series of the campaigns on the in RVs *Atlantic II*, *Knorr* and *Chain* which explored the western Atlantic from the coast of United States (Gay Head-Bermuda transect) through Guiana and Brazilian basins down to the Argentinian coasts between 1966 and 1972^20^. Further, a small group of specimens came from the SLOPE project gathered during the Australian program assessing diversity in the Bass Strait (SE Australia^21^). All of those collections were first fixed in 4% buffered formaldehyde and transferred into 75–80% ethanol for sorting to the highest taxonomical levels.

Recently, more material was collected on board RV *Meteor* in 2011 during the Icelandic Animals Genetic and Ecology (IceAGE) program in waters of Iceland^22^, and on board RV *Sonne*, in 2014/2015 on both sides of the Mid-Atlantic Ridge at the Vema Fracture Zone during the Vema-TRANSIT project^23^, and in 2015 from the Central Pacific within the framework of the Joint Programming Initiative Healthy and Productive Seas and Oceans platform (JPI Oceans)^24^. That material was collected, fixed and handled following protocols for fixation as described in Riehl *et al*.^25^ and was used for molecular analyses. Information about all the expeditions during which the material used for this study was collected is gathered in Table 1.

**Table 1:** Localities and expedition of the stations from where Tanaidacea for the present studies were collected. RV – research vessel, N – number of individuals, EBS – epibenthic sledge; GKG – box corer; MIG – McIntyre grab.

Location	Date	RV	Cruise	Station	Position		Depth (m)	gear	N
					longitud	latitude			
**Atlantic Ocean**									
Norwegian Basin	17/09/2011	*Meteor*	IceAGE1	1155	69º06.89′N	09º54.72′W	2177–2174	EBS	6
Norwegian Basin	21/09/2011	*Meteor*	IceAGE1	1191	67º04.72′N	13º03.83′W	1577–1578	EBS	2
Porcupine Seabight	22/08/1972	*Chain*	106	326	50º04.90′N	14º23.80′W	3859	EBS	1
Gay Head-Bermuda	01/05/1966	*Chain*	58	100	33º56.80′N	65º47.00′W	4743–4892	EBS	1
Gay Head-Bermuda	16/08/1966	*Atlantis II*	24	115	39º39.20′N	70º24.50′W	2030–2051	EBS	2
Gay Head-Bermuda	20/08/1966	*Atlantis II*	24	120	34º43.00′N	66º32.80′W	5018–5023	EBS	1
Gay Head-Bermuda	21/08/1966	*Atlantis II*	24	121	35º50.00′N	65º11.00′W	4800	EBS	1
Gay Head-Bermuda	22/08/1966	*Atlantis II*	24	122	35º50.00′N	64º57.50′W	4833	EBS	10
Gay Head-Bermuda	18/12/1966	*Atlantis II*	30	131	39º38.50′N	70º36.50′W	2178	EBS	2
Gay Head-Bermuda	23/02/1969	*Chain*	88	210	34º43.00′N	70º46.00′W	2024–2064	EBS	2
Gay Head-Bermuda	24/11/1973	*Knorr*	35	340	38º14.40′N	70º20.30′W	3264–3356	EBS	5
Vema Fracture-Zone	02/01/2015	*Sonne*	Vema-TRANSIT	6–8	10º22.25′N	36º56.05′W	5137–5127	EBS	1
off Brazil	14/02/1967	*Atlantis II*	31	156	00º46.00′S	29º24.00′W	3459	EBS	19
Argentine Basin	14/03/1971	*Atlantis II*	60	245	36º55.70′S	53º01.40′W	2707	EBS	6
Argentine Basin	27/03/1971	*Atlantis II*	60	262	36º05.20′S	52º17.90′W	2440–2480	EBS	13
Argentine Basin	15/07/2009	*Meteor*	DIVA3	533	36º00.20′S	49º01.96′W	4601.8–4605.7	EBS	1
**Pacific Ocean**									
Clarion Clipperton Zone	01/04/2015	*Sonne*	JPIO	81	11º03.97′N	119º37.67′W	4401.4–4397.9	EBS	4
Clarion Clipperton Zone	03/04/2015	*Sonne*	JPIO	95	11º04.41′N	119º39.35′W	4418.3	GKG	2
Clarion Clipperton Zone	04/04/2015	*Sonne*	JPIO	99	11º02.28′N	119º40.89′W	4601.8–4605.7	EBS	2
Clarion Clipperton Zone	05/04/2015	*Sonne*	JPIO	106	11º04.30′N	119º39.29′W	4425.3	GKG	1
Bass Strait	22/07/1986	*Franklin*	SLOPE	25	38º25.54′S	148º58.36′E	1850	EBS	1
Bass Strait	15/05/1994	*Franklin*	SLOPE	140	38º57.44′S	141º37.04′E	1450	EBS	1
Bass Strait	15/05/1994	*Franklin*	SLOPE	142	38º59.43′S	141º33.18′E	1975	EBS	2
Bass Strait	21/05/1994	*Franklin*	SLOPE	170	37º05.53′S	137º42.32′E	1548	MIG	1

Additionally, the type species of *Paranarthrurella*, *P. caudata* (Kudinova-Pasternak, 1965), borrowed from the Zoological Museum in Moscow, was examined and redescribed^2^. Furthermore, in order to provide a complete description of *Paranarthurella arctophylax* (Norman and Stebbing, 1886) material collected in the Iceland Basin during BIOICE (Benthic Invertebrates of Icelandic waters) project was also examined and consequentely redescribed. Unfortunately, the other poorly-described historical types — *Arthrura shiinoi* Kudinova-Pasternak, 1973, *Armatognathia birsteini* Kudinova-Pasternak, 1987 and *Leptognathia dissimilis* Lang, 1972 — were inaccessible for our study.

## Methods

### Taxonomic analyses

A total of 87 individuals of nine new species was examined morphologically with a Leica M125 stereomicroscope. For all specimens, the body length (BL) was measured from the tip of the rostrum to the distal edge of the pleotelson.

Seven life stages are recognized for the studied individuals: two stages of manca, one neuter, two stages of females, and two stages of males. The terms “manca-2” and “manca-3” refers to specimens without or with buds of pereopod-6, respectively^26^; ‘preparatory female’ and ‘ovigerous female’ are bearing oostegites buds either fully developed oostegites, respectively; ‘juvenile male’ and ‘mature male’ (swimming)^27^ show incompletely or completely developed sexual dimorphic characters, respectively. Finally, the term ‘neuter’ is retained for the stage developed from manca-3 that cannot be classified as precopulatory female with oostegites buds (including non-ovigerous females), or juvenile male. Appendages from the chosen specimens were dissected in a glycerine solution using chemically-sharpened tungsten needles, mounted in glycerine on slides, and sealed with paraffin wax. For staining, methylene blue or chlorazol black were used.

Initial drawings were made using a Nikon Eclipse 50*i* microscope combined with a *camera lucida*; they were then digitally inked as proposed by Coleman^28^.

The general morphological terminology follows that proposed by Jóźwiak *et al*.^16^. The body length-to-width ratio was assessed dividing a measurement of total body length (see above) and by a measurement the widest part of cephalothorax. Length of the articles and segments was measured along the central axis, whereas width was assessed at the mid-length of the article. As proposed by Bird and Bamber^29^, the ‘spines’ are called the articulated and unflexible cuticular structures while ‘setae’ are flexible and bristle or hair-like articulated structures; the apophyse (or teeth) are reserved for the nonarticulated cuticular outgrowth. The short, weakly calcified, round tip setae in mandibule molar of *Paranarthrurella* are called finger-shape setae.

All of the measurements were performed with Leica M205C and LAS V4.5 software. To simplify species descriptions, the expression ‘*N*x’ replaces ‘*N* times as long as’ and ‘*N* L:W’ replaces ‘*N* times longer than wide’.

Photographs were made using the focus stacking method on a Leica M205C stereo-microscope combined with a DFC295 camera and LAS V4.5 software. For the redescription of *Paranarthrurella arctophylax*, confocal microscopy imaging was used. Images were registered with the confocal laser scanning microscope LSM 780 (Zeiss) equipped with EC Plan-Neofluar 10x/0.30 M27 objective, 405 nm laser diode and and InTune tunable excitation laser system (set to excitation wavelength 595 nm). Natural autofluorescence of the specimen was enhanced by chemical crosslinking by transient incubation in formalin. Otherwise unstained, ethanol-fixed animal was imaged in 100% glycerol. Autofluorescence was registered sequentially in two emission channels: 410–580 nm (405 nm excitation) and 600–735 nm (595 nm excitation). In 3 × 3 tile scan area (3188.22 µm × 3188.22 µm), images were collected for stitching along with optimal number of Z-frames, each in 2048 × 2048 pixels format with 3.15 µs pixel dwell and 2 × line averaging. Images in both channels were then combined, pseudo-colored in gold and reconstructed into 2D image stack by maximum intensity projection using ZEN 2012 software (Zeiss).

The type material is deposited in the Museum of Comparative Zoology (MCZ, United States), Zoological Museum of Hamburg (ZMH, Germany) and Melbourne Museum (NMH, Australia). The material for the redescription of *Paranarthrurella arctophylax* is deposited in the Icelandic Institut of Natural History (NI, Iceland).

### DNA extraction, alignment, and phylogenetic analysis

Single appendages (chela or pereopod-1) were taken from each specimen for DNA extraction using sterile needles, and transferred to buffer solution. Extraction of DNA was performed at the Smithsonian Laboratories for Analytical Biology^25^. Two markers were sequenced: the nuclear ribosomal small subunit (18S), and the mitochondrial cytochrome *c* oxidase subunit I (COI). Polymerase Chain Reactions (PCR) protocols, primer sequences, and sequencing protocols were according to Riehl *et al*.^25^.

Sequencing reads were assembled in Geneious v. 11.1.4 and checked by hand to resolve ambiguities and remove primer sequences. To place sequences of *Paranarthrurella* in the proper phylogenetic context, nine sequences of 18S and 14 of COI from other genera within superfamily Paratanaoidea were obtained from GenBank (Agathotanaidae, Akanthophoreidae, Cryptocopidae, Paratanaidae, Leptocheliidae, Nototanaidae, Typhlotanaidae; Table 2 ^27,30–35^). Finally, a gammarid species for which both COI and 18S sequences were accessible in GenBank.

**Table 2:** Details on the tanaidacean taxa used for genetic analysis applying COI and 18S markers.

Species	Specimen code	18S	COI	Reference
**Agathotanaidae Lang, 1971**				
*Agathotanais ingolfi* Hansen, 1913	ITan029		KJ934610	Błażewicz-Paszkowycz *et al*.^27^
*Agathotanais ingolfi* Hansen, 1913	ITan053		KJ934609	Błażewicz-Paszkowycz *et al*.^27^
*Paranarthrura* sp.		AB618196		Kakui *et al*.^31^
**Akanthophoreidae Sieg, 1986**				
*Akanthophoreus* cf. *alba*	ITan165	**MK804189**		this paper
*Brixia aurora* Jóźwiak, Drumm, Bird & Błażewicz, 2018	ITan028	**MK804190**	KJ934607	this paper; Jóźwiak *et al*.^30^
*Brixia aurora* Jóźwiak, Drumm, Bird & Błażewicz, 2018	ITan117		KJ934608	Jóźwiak *et al*.^30^
*Chauliopleona armata* (Hansen, 1913)	ITan059	**MK804188**		this paper
*Chauliopleona* sp.		AB618200		Kakui *et al*.^31^
**Colletteidae Larsen and Wilson, 2002**				
*Collettea* aff. *wilsoni*	ITan017	**MK804191**		this paper
*Collettea* sp.	ITan057	**MK804192**		this paper
**Cryptocopoidae Sieg, 1977**				
*Cryptocopoides* aff. *arcticus*	ITan092		KJ934611	Błażewicz-Paszkowycz *et al*.^27^
*Cryptocopoides* aff. *arcticus*	ITan122		KJ934612	Błażewicz-Paszkowycz *et al*.^27^
**Leptocheliidae Lang, 1973**				
*Chondrochelia dubia* (Krøyer, 1842)			HM016215	Drumm 2010
*Chondrochelia dubia* (Krøyer, 1842)			JX402115	Larsen *et al*.^34^
*Leptochelia forresti* Stebbing, 1896			HM016206	Drumm 2010
*Leptochelia forresti* Stebbing, 1897			KP255266	Leray and Knowlton^33^
*Leptochelia longichelipes* (Lang, 1973)			HM016201	Drumm 2010
*Makassaritanais itoi* (Ishimaru, 1985) (=*Leptochelia itoi)*		AB618197		Kakui *et al*.^31^
*Leptochelia* sp.		AF496660		Wägele *et al*. unpublished 2004
**Paranarthrurellidae fam. nov**.				
*Paranarthrurella polonez* sp. nov.	ind 171	**MK804182**		this paper
*Paranarthrurella polonez* sp. nov.	ind 182	**MK804183**		this paper
*Paranarthrurella* sp.1	ITan159		**MK751353**	this paper
*Paranarthrurella* sp.1	ITan163		**MK751356**	this paper
*Paranarthrurella* sp.1	ITan164		**MK751357**	this paper
*Paranarthrurella* sp.2	ITan158	**MK804177**	**MK751352**	this paper
*Paranarthrurella* sp.2	ITan160	**MK804178**	**MK751354**	this paper
*Paranarthrurella* sp.2	ITan162	**MK804179**		this paper
*Paranarthrurella* sp.2	ITan161		**MK751355**	this paper
*Paranarthrurella* sp.2	ITan170		**MK751358**	this paper
**Paratanaidae Lang, 1949**				
*Metatanais* sp.		AB618201		Kakui *et al*.^31^
*Aparatanais malignus* (Larsen, 2001) (=*Paratanais malignus*)		AY781429		Larsen^32^
*Paratanais* sp.		AB618199		Kakui *et al*.^31^
**Nototanaidae Sieg, 1976**				
*Nesotanais ryukyuensis* Kakui, Kajihara & Mawatari, 2010		AB618198		Kakui *et al*.^31^
**Tanaellidae Larsen and Wilson, 2002**				
*Tanaella ochracea* Hansen, 1913	ITan027	**MK804194**		this paper
*Tanaella unguicillata* Norman & Stebbing, 1886	ITan015	**MK804193**		this paper
*Tanaella unguicillata* Norman & Stebbing, 1886	ITan043	**MK804195**		this paper
*Tanaella unguicillata* Norman & Stebbing, 1886	ITan135	**MK804196**		this paper
**Typhlotanaidae Sieg, 1984**				
*Typhlotanais cornutus* (G.O. Sars, 1879)	ITan040		**MK751359**	this paper
*Typhlotanais cornutus* (G.O. Sars, 1879)	ITan041		**MK751360**	this paper
*Typhlotanais eximus* Hansen, 1913	ITan076		**MK751361**	this paper
*Typhlotanais eximus* Hansen, 1913	ITan128		**MK751362**	this paper
*Typhlotanais mixtus* Hansen, 1913	ITan021	**MK804185**		this paper
*Typhlotanais trispinosus* Hansen, 1913	ITan014	**MK804184**		this paper
*Typhlotanais variabilis* Hansen, 1913	ITan050		KJ934599	Błażewicz-Paszkowycz *et al*.^27^
*Typhlotanais variabilis* Hansen, 1913	ITan068	**MK804186**	KJ934602	this paper; Błażewicz-Paszkowycz *et al*.^27^
Typhlotanais variabilis Hansen, 1913	ITan069	**MK804187**	KJ934600	this paper; Błażewicz-Paszkowycz *et al*.^27^

Alignment of 18S was performed with the online MAFFT server v7^36^, followed by deletion of poorly-aligned regions using the online Gblocks server^37^, employing all three options for less-stringent selection. Alignment of COI was performed on DNA codons using the ClustalX algorithm^38^ in BioEdit (written by Tom Hall, Ibis Theraputics). Both alignments were trimmed to remove large front- and back-end blocks of gaps. Twenty-nine new sequences obtained for this project were deposited in GenBank (Accessions MK751352– MK751362 and MK804177–MK804196, Table 2). Pairwise distances within *Paranarthrurella*, within non-*Paranarthrurella*, and between the two groups were calculated in MEGA5^39^ using the K2P distance model^40^.

Bayesian phylogenetic trees were computed in BEAST 2.5.0^41^ using a four-category gamma-distributed model of sequence mutation for both markers. For COI, the HKY mutational model was employed, whereas for 18S the GTR model was used. Strict clocks and Yule tree priors were used for both markers. All gamma priors for GTR rates were replaced with default lognormal priors. Convergence of the runs was assessed with Tracer v1.6^41^ to choose a burn-in such that all effective sample sizes (ESSs) were at least 200. Consensus trees were produced and annotated with Bayesian posterior probabilities (PP) using TreeAnnotator in the BEAST2 package. Because there were few ingroup specimens for which both 18S and COI were obtained (five), no multilocus tree was estimated.

### Species delimitation

Species delimitation was performed on COI sequences of *Paranarthrurella* using three methods: the ABGD algorithm (Automated Barcode Gap Discovery)^42^, GMYC method (General Mixed Yule Coalescent)^43^, and mPTP algorithm (Multiple Threshold PTP)^44^. Because species delimitation (SD) analyses can be sensitive to the taxonomic depth of the tree, a pruned dataset was used, including only *Paranarthrurella* and *Cryptocope* sequences. The ABGD method required the alignment as input, and was performed using the online version (snv.jussieu.fr/public/abgd/abgdweb.html) on Kimura 2-parameter (K2P)^45^ corrected pairwise distances and 20 algorithm steps. The GMYC and mPTP analyses required the ultrametric tree produced by BEAST; GMYC analysis was conducted in R with the single-threshold option, and mPTP was performed using the command-line software, employing 20,000 burn-in steps and three replicate runs.

### Nomenclatural acts

The electronic edition of this article conforms to the requirements of the amended International Code of Zoological Nomenclature, and hence the new names contained herein are available under that Code from the electronic edition of this article. This published work and the nomenclatural acts it contains have been registered in ZooBank, the online registration system for the ICZN. The ZooBank LSIDs (Life Science Identifiers) can be resolved and the associated information viewed through any standard web browser by appending the LSID to the prefix “http://zoobank.org/”. The LSID for this publication is: urn:lsid:zoobank.org:pub: E6E09F88-9F8B-4FB9-9FAB-078CFB893D40. The electronic edition of this work was published in a journal with an ISSN, and has been archived and is available from the following digital repositories: PubMed Central and LOCKSS.

### Principal component analysis

To quantify the characters used for distinguishing the genus *Paranarthrurella* from *Armatognathia*, a Principal Component Analysis (PCA) was conducted on 13 morphological features for the 16 currently known species of the new family herein described (Table 3). The analysis was run on normalized data using PRIMER 5 software^46^

**Table 3:** Morphological characters used to define the genera *Paranarthrurella* and *Armatognathia* by means of Principal Component Analysis (PCA): BL, body length; H, hyposphaenium; A2, antenna; Mdb, mandible; Mxp gc, maxilliped endite gustatory cusps; Ch, chela; Chp, cheliped; Chp b, Cheliped basis distal part; Pl, pleopods; U, uropod; end, endopod; exo, exopod; —: data not available.

Character species	BL	H2	H3	H4	A2	Mdb	Mxp gc	Ch	Chp	Chp b	Pl ♀	U end article 1/2	U end/exo	Source
*A. birsteini*	long	absent	absent	absent	spine	spines	tubercles?	robust	1.1	long	present	longer	1.6	Kudinova-Pasternak^17^
*A. milonga* sp. nov.	long	absent	absent	absent	spine?	spines	tubercles	robust	1.0	long	present	longer	—	current study
*A. shiinoi*	long	—	—	—	spine?	spines?	tubercles?	robust	1.4	long	present	longer	1.7	Kudinova-Pasternak^86^
*A. swing* sp. nov.	long	absent	absent	absent	spine	spines	tubercles	robust	1.0	long	present	longer	1.8	current study
*P. arctophylax*	short	absent	well developed	absent	seta	palpate setae?	long?	slender	1.3	short	absent	subequal	—	Norman and Stebbing 1886
*P. caudata*	long	absent	absent	absent	seta	palpate setae	long	slender	1.6	short	absent	subequal	1.5	Kudinova-Pasternak^2^, current study
*P. corroboree* sp. nov.	short	small	well developed	absent	seta	palpate setae	palpate setae	slender	1.4	short	absent	subequal	1.5	current study
*P. dissimilis*	long	absent	absent	absent	seta	palpate setae	long	slender	1.4	short	absent	subequal	1.3	Lang^9^
*P. kizomba* sp. nov.	long	absent	absent	absent	seta	palpate setae	palpate setae	slender	1.3	short	absent	subequal	1.6	current study
*P. moonwalk* sp. nov.	short	small	well developed	absent	seta	palpate setae	palpate setae	slender	1.1	short	absent	subequal	1.6	current study
P. polonez sp. nov.	long	absent	absent	absent	seta	palpate setae	palpate setae	slender	1.6	short	absent	subequal	1.1	current study
*P. rocknroll* sp. nov.	short	absent	small	absent	seta	palpate setae	palpate setae	slender	1.3	short	absent	subequal	—	current study
*P. samba* sp. nov.	long	absent	absent	absent	seta	palpate setae	palpate setae	slender	1.6	short	absent	subequal	1.4	current study
*P. spinimaxillipeda*	long	absent	absent	absent	seta	palpate setae?	—	slender	1.4	short	absent	subequal	1.4	Larsen and Araújo-Silva^18^
*P. tango* sp. nov.	short	absent	small	absent	seta	palpate setae	palpate setae	slender	1.2	short	absent	subequal	1.2	current study
*P. voeringi*	short	well developed	well developed	small	seta	palpate setae	palpate setae	slender	1.4	short	absent	subequal	1.5	Jóźwiak *et al*.^16^, current study

Morphological characters:

1. Body length (BL): short (length/width ratio <6); long (length /width ratio >8).
2. Hyposphaenium (H2) of pleonite-2: absent; small; well developed.
3. Hyposphaenium (H3) of pleonite-3: absent; small; well developed.
4. Hyposphaenium (H4) of pleonite-4: absent; small.
5. Antenna (A2) article-2 ornamentation: spine; seta.
6. Mandible (Mdb) molar ornamentation: finger-shape (tip rounded) setae; robust spines.
7. Maxilliped endite gustatory cusps (Mxp gc): slender; round (tubercles).
8. Chela (Ch): slender (>1.6 L:W); robust (<1.3 L:W).
9. Cheliped (Chp) carpus length/width.
10. Cheliped basis (Chp b) distal part: long (dorsal concave in midlength); short (dorsal concave in distal half);
11. Pleopods (Pl) in female: present; absent.
12. Uropod (U) endopod article-2/article-1 ratio: subequal; longer.
13. Uropod (U) exopod/endopod length.

## Results

Morphological analysis of 87 individuals provisionally classified to *Paranarthrurella* allowed the observation of a series of morphological features (e.g.: left mandible *lacinia mobilis* broad, widely separated from incisor forming “spoon-like” gap, rounded and swollen pleon) which are also present in a few other species known from the literature, such as *Armatognathia birsteini* Kudinova-Pasternak, 1987 and the tanaellid *Arthrura shiinoi* Kudinova-Pasternak, 1973, and the colletteid recently described *Cheliasetosatanais spinimaxillipedus* Larsen & Araújo-Silva, 2014. The morphological analysis of those synapomorphies allowed us to propose to erect a new family — Paranarthrurellidae **fam. nov**. — which include the three aforementioned species as well. The morphological analysis was supported by genetic results (see below). The investigation of the material has revealed the presence of seven new species which represent the genus *Paranarthrurella* and two which represent the genus *Armatognathia*.

### Genetics

**Figure 1:**
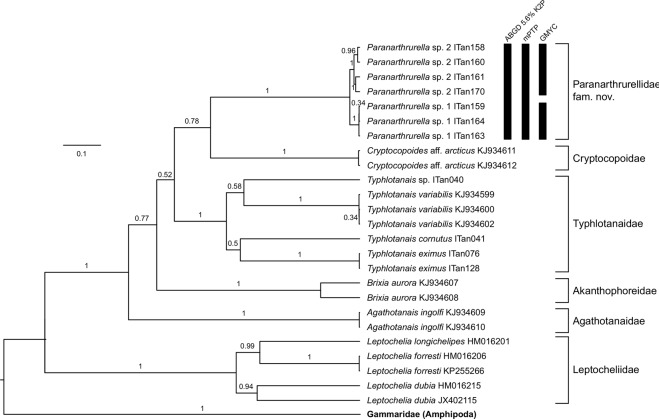
Species delimitation on the COI data using the ABGD algorithm (automatic barcode gap discovery), GMYC (general mixed Yule coalescent), and mPTP (multiple threshold PTP).

**Figure 2:**
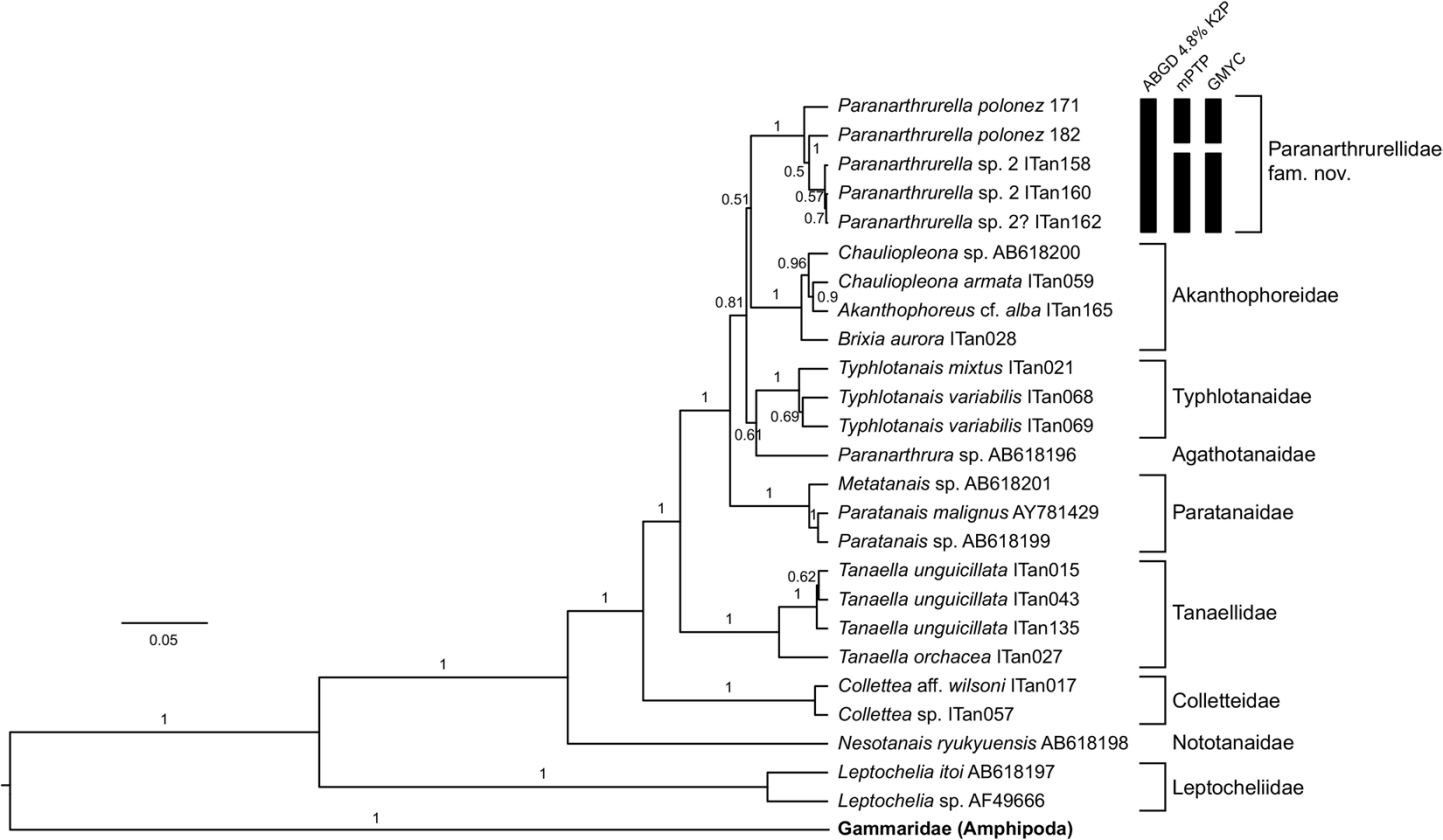
The maximum-parsimony trees based on 18S rRNA gene sequence data demonstrating monophyly of six families e.g. Akanthophoreidae Sieg, 1986, Typhlotanaidae Sieg, 1984, Agathotanaidae Lang, 1971, Paratanaidae Lang, 1949, Nototanaidae Sieg, 1976 and Leptocheliidae Lang, 1973, and two genera: *Tanaella* Norman and Stebbing, 1886 and *Collettea* Lang, 1973.

From the 16 individuals of genus *Paranarthrurella* that were preserved for molecular analysis, we were able to successfully amplify six sequences for 18S and seven sequences for COI. The PCR success was rather low (38% and 44%) which is rather typical for small and poorly-investigated deep-sea organisms (Table 3). Pairwise K2P distances for COI within the proposed Paranarthrurellidae **fam. nov**. were much smaller than those between members of this family and other tanaids, although genetic diversity was low in the group due to the small sampling of taxa (Table 4). Nevertheless, phylogenetic analysis recovered a monophyletic Paranarthrurellidae with maximum support in both loci (Figs 1 and 2). The GMYC analysis of *Paranarthrurella* COI sequences (n = 7) delimited two species, one corresponding to *Paranarthrurella* sp. 1 and the other to *Paranarthrurella* sp. 2; ABGD and mPTP analyses delimited all sequences to a single species (Fig. 1). Both the GMYC and mPTP analyses grouped 18S sequences of *Paranarthrurella* into two species corresponding to *P. polonez* sp. nov. and *Paranarthrurella* sp.2; ABGD analysis delimited all 18S sequences to a single species (Fig. 2).

**Table 4:** Genetic distance based on COI and 18S markers between *Paranarthrurella* (P) and non-*Paranarthrurella* (NP) taxa, and between both groups.

		COI	18S
**within P**			
	min	0	0
	max	0.05816	0.022
	mean	0.0280	0.01256
**within NP**			
	min	0	0.0089
	max	0.73081	0.3111
	mean	0.4629	0.1333
**between P and NP**			
	min	0.39796	0.04065
	max	0.68622	0.35183
	mean	0.5289	0.10706

### Principal component analysis

**Figure 3:**
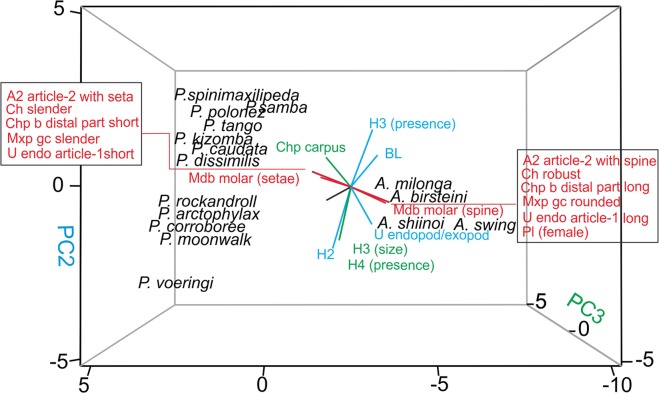
Principal Components Analysis plot (PCA) of twelve *Paranarthrurella* species and four *Armatognathia* species and thirteen characters listed in the Table 3.

PCA divides the taxa onto three groups (Fig. 3). The first, second and third axes explain 52.7%, 15.3% and 8.4% of total variation, respectively. Axes 4 and 5 (not illustrated on Fig. 3) explain together only 11.4% of a variation. At first glance, two groups of features clearly differentiate the two genera, *Armatognathia* and *Paranarthrurella*, along axis 1. The genus *Armatognathia* is characterised by: presence of spine on antennular article-2, presence of spines on the mandible molar, rounded gustatory cusps (tubercles) on the maxilliped endites, robust chela, cheliped distal part long, presence of pleopods in females, and article-1 of the uropod endopod longer than article-2. The genus *Paranarthrurella* is characterised by: presence of a seta on antennulal article-2, finger-shape setae on the mandible molar, slender gustatory cusps (spine-like) on the maxilliped endites, slender chela, cheliped distal part short, absence of pleopods in females, and subequal articles 1 and 2 of the uropod endopod.

Axes 2 and 3 cluster *Paranarthrurella* into two species groups. The first group, which includes *P. spinimaxillipeda, P. samba* sp. nov.*, P. polonez* sp. nov.*, P. tango* sp. nov.*, P. kizomba* sp. nov.*, P. caudata* and *P. dissimilis*, has a longer body (except *P. tango* sp. nov.), uropod endopod/exopod length ratio lower and absence of hyposphaenium on pleonites 2 and 3. Additionally, *P. polonez* sp. nov. and *P. samba* sp. nov. have an elongated cheliped carpus. In the second group, *P. rocknroll* sp. nov., *P. arctophylax*, *P. corroboree* sp. nov. and *P. moonwalk* sp. nov. have short bodies, presence of hyposphaenium on pleonites 2 and 3 (only in *P. rocknroll* the hyposphaenium on pleonite 2 is absent), uropod endopod/exopod length ratio higher and the cheliped carpus L/W ratio is smaller. *Paranarthrurella voeringi* is an outlier in the distinct presence of small hyposphaenium in pleonite 4 and large hyposphaenium on 3. Additionally, features localised on pereopods as follows: pereopod 4–6 propodus spine, pereopod-1 merus/carpus spines were correlated with PC Axes 4 and 5, are not illustrated on Fig. 3. They do not play a significant role in morphological differentiation of the two genera, but are important for discrimination of particular species.

## Systematics and Taxonomy

Suborder Tanaidomorpha Sieg, 1980

Superfamily Paratanaoidea Lang, 1949

**Family Paranarthrurellidae fam. nov. Błażewicz, Jóźwiak and Frutos**

urn:lsid:zoobank.org:act:F9179CD9-B880-4A25-BE83-33CF8F5F5E02

### Diagnosis

Body strongly calcified with six well developed pereonites and five distinct pleonites; eyes absent; antennule with four articles, with additional cap-like article; antennae with six articles; mandible molar wide, rounded with row of blunt distal spines; mandible left lacinia mobilis broad, widely separated from incisor forming “spoon-like” gap; maxillule endite with distal spines and numerous setae along outer margin; maxilliped basis fused, endites separated, with two round and well-developed (tubercle) or slender spine-like (gustatory cusps); cheliped attached to cephalothorax with sclerite; sclerite large, triangular (proximal margin little shorter than sclerite main axis); pereopods 1–3 with coxa, merus and carpus with usually small and fine spines; pereopods 4–6 carpus with four spines; pereopod-6 propodus with three distodorsal spines; uropods shorter than pleotelson, biramous, each rami with two articles.

Males: Pleotelson with terminal elongated apophysis; antennule with seven articles (three peduncular and four flagellar); mouthparts reduced; maxilliped present; pleopods well developed; uropod endopod with three articles, exopod with three articles.

Type genus: *Paranarthrurella* Lang, 1971

Genera included: *Armatognathia* Kudinova-Pasternak, 1987; *Paranarthrurella* Lang, 1971

### Remarks

The current systematics of the superfamily Paratanaoidea rely on the number of articles in the antennule (three or four, regardless that some families retain a fifth vestigial article^47^), character of the uropod exopod, and a character of the cheliped attachment to cephalothorax^15,48^. From this perspective the new family is readily distinguished from:

- Heterotanaoididae Bird, 2012^49^. Leptocheliidae Lang, 1973^50^, Nototanaidae Sieg, 1976^51^ Pseudotanaidae Sieg, 1976^51^, Pseudozeuxidae Sieg, 1982^52^, Tanaissuidae Bird and Larsen, 2009^48^, and Typhlotanaidae Sieg, 1984^53^ which have three, well developed articles on the antennule – by presence of four well developed articles in antennule;
- Agathotanaidae Lang, 1971^1^ and Anarthruridae Lang, 1971^1^, which have pseudocoxa – by having cheliped attached to cephalothorax by sclerite (= cheliped basis with a free posterior lobe).
- Tanaellidae Larsen and Wilson, 2002^15^, which missing or fused exopod in uropods – by having well developed both exopod and endopod in uropods;
- Mirandotanaidae Błażewicz-Paszkowycz and Bamber, 2009^54^ which have enlarged pleotelson^54^ – by having regularly developed pleotelson;
- Teleotanaidae Bamber, 2008^55^ and Paratanaidae Lang, 1949^56^ – by absence of the eyes and absence of the robust lateral seta on pleonites 1–4; additionally shallow-water Teleotanaidae have dark pigmentation of tegument that never occurs in deep-water Paranarthrurellidae;
- Cryptocopidae Sieg, 1977^57^, by shape of the sclerite that connect cheliped to tegument. It is very narrow and almost parallel to ventroproximal corner of the carapace^58^, while it is wide and triangular in Paranarthrurellidae fam. nov.;
- Tanaopsidae Błażewicz-Paszkowycz and Bamber, 2012^59^ – by absence of the crenulation on cheliped dactylus and coxal spur on pereopod-1;
- Akanthophoreidae Sieg, 1986^14^ – by presence of relatively short and robust uropods. In contrast to Paranarthrurellidae, Akanthophoreidae uropods are always slender and elongated. Yet, Akanthophoreidae have often robust and prominent spines on merus and carpus of the pereopods 1–3, while Paranarthrurellidae fam. nov. have usually slender and relatively short spines in those appendages.

From the list of 18 families, two of them, namely Colletteidae Larsen and Wilson, 2002 and Leptognathiidae Sieg, 1976 are obviously polyphyletic and/or repository taxa with biramous uropods, antennule with four articles, with broad (Colletteidae) or pointed (Leptognathiidae) mandible molar; certainly, both families require severe revision. Until such analysis can be done, the new family can still be distinguished from those two families by characteristic mouthpart details (wide mandible molar with row of blunt distal spines; left *lacinia mobilis* broad and widely separated from incisor forming “spoon-like” gap; maxillule endite numerous setae along outer margin; maxilliped endites separated, with two well-developed or slender gustatory cusps and peculiar, rounded pleotelson).

### Genus *Paranarthrurella* Lang, 1971

*Tanais* Latreille, 1831^5^ (partim.): G.O. Sars, 1877: 347, 370^6^; G.O. Sars, 1882: 50^7^; G.O. Sars, 1896: 33; Sieg, 1980: 11–12^60^.

*Cryptocope* G.O. Sars, 1882^7^ (partim.): G.O. Sars, 1882: 50–51^7^; G.O. Sars, 1885: 74–78^61^; Forsstrand, 1886: 47^62^; Norman, 1899: 340^63^; Zirwas, 1911: 105^64^; Hansen, 1913: 106, 109–110, pl X^8^; Nierstrasz, 1913: 33^65^; Lang, 1949: 6, 8^56^; Stephensen, 1932: 349^66^; Lang, 1971: 403^1^.

*Leptognathia* G.O. Sars, 1882 (partim.)^7^: Kudinova-Pasternak, 1965: 75, 88–91^2^; Belyaev, 1966: 88^67^; Kudinova-Pasternak, 1968: 73^68^; Lang, 1968: 160–161^69^; Lang, 1971: 361–362^70^; Gardiner, 1975: 225^71^; Lang, 1972: 229–235^9^; Kudinova-Pasternak, 1981: 115^3^; Sieg 1983: 317^72^; Sieg 1986 168, 170^73^.

*Strongylura* G.O. Sars, 1882 (partim.): Norman and Stebbing, 1886: 110, 133^12^.

*Paranarthrurella* Lang, 1971: 361, 363, 367^70^; Sieg, 1973: 34–281^74^; Sieg, 1976: 178^51^; Sieg, 1978: 121^75^.

*Robustognathia* Kudinova-Pasternak, 1989: 68, 33–34^4^.

*Biarticulata* Larsen and Shimomura, 2007 (partim)^13^: 19; Bird, 2007: 75^76^.

*Cheliasetosatanais* Larsen and Araújo-Silva, 2014: 969–972^18^; Larsen, Gutu and Sieg 2015: 257, 260, 304, Fig. 59.3^77^; Wi, Suh and Kim, 2015: 725^78^; Morales-Nuñez, Larsen and Cooke, 2016: 11, Tab^79^.

Type species: *Paranarthrurella caudata* (Kudinova-Pasternak, 1965)

Species included: *Paranarthrurella arctophylax* (Norman and Stebbing, 1886), *P. caudata* (Kudinova-Pasternak, 1965), *P. corroboree* sp. nov., *P. dissimilis* (Lang, 1972), *P. kizomba* sp. nov., *P. moonwalk* sp. nov., *P. polonez* sp. nov.; *P. rocknroll* sp. nov.; *P. samba* sp. nov.; *P. spinimaxillipeda* (Larsen and Araújo-Silva, 2014), *P. tango* sp. nov., *P. voeringi* (Sars, 1877).

### Diagnosis

Female: Body long or short (five to ten L:W). Pereonites subrectangular or subsquare; pereonites 1–3 usually slightly wider anteriorly; pereonite-6 slightly wider posteriorly. Antenna article-2 with seta distally. Molar with short (finger-shape) spines. Maxilliped endites with slender gustatory cusps. Cheliped carpus slender (usually >1.6 L:W). Cheliped basis distal part short (dorsal concave in distal half). Chela slender. Pleopods absent in females. Uropod endopod article-1 subequal article-2.

***Paranarthrurella caudata*** **(Kudinova-Pasternak, 1965)**

Figures 4−6

**Figure 4:**
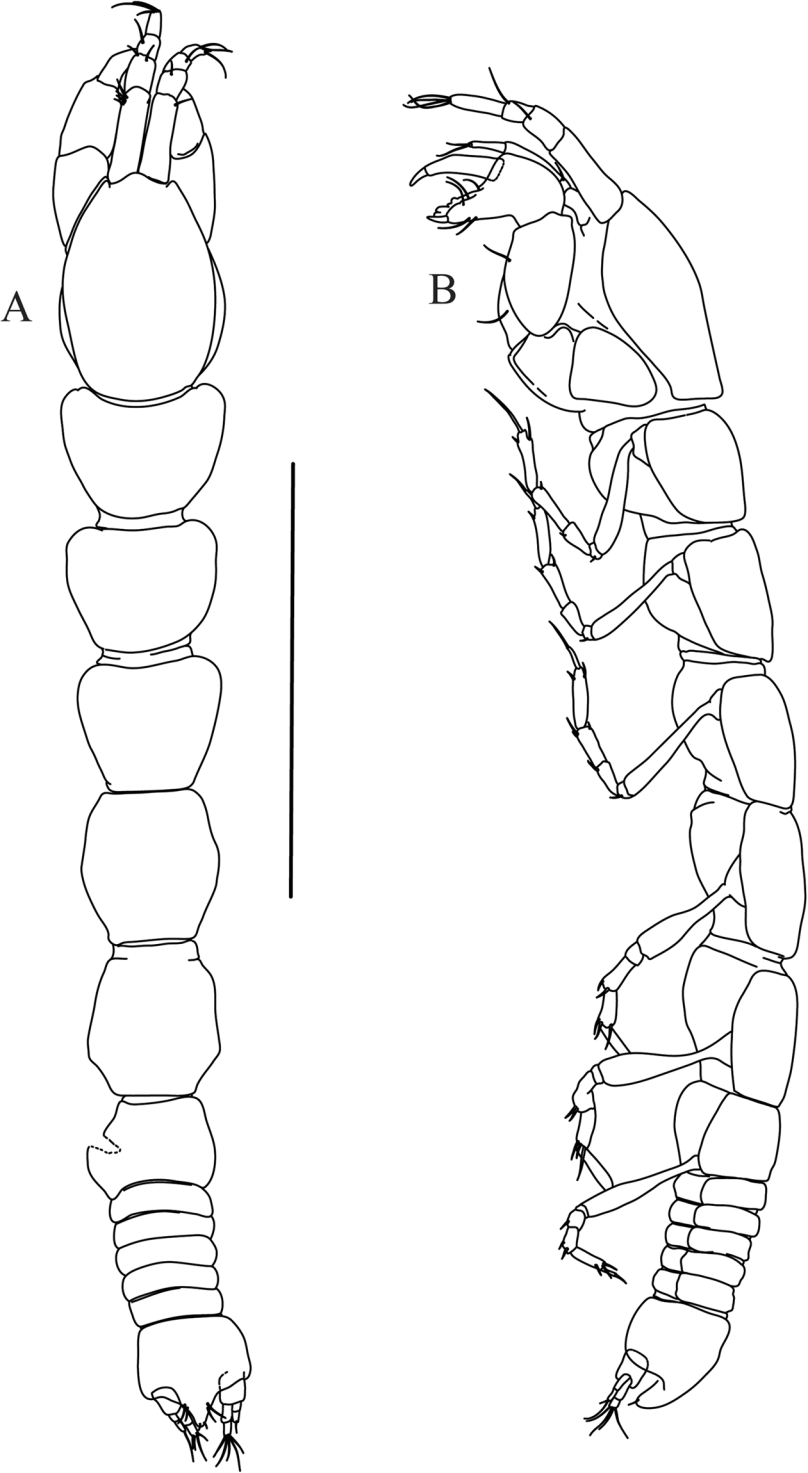
*Paranarthrurella caudata* (Kudinova-Pasternak, 1965), holotype, female (Mc-938). (**A**) Dorsal. (**B**) Lateral. Scale 1 mm.

**Figure 5:**
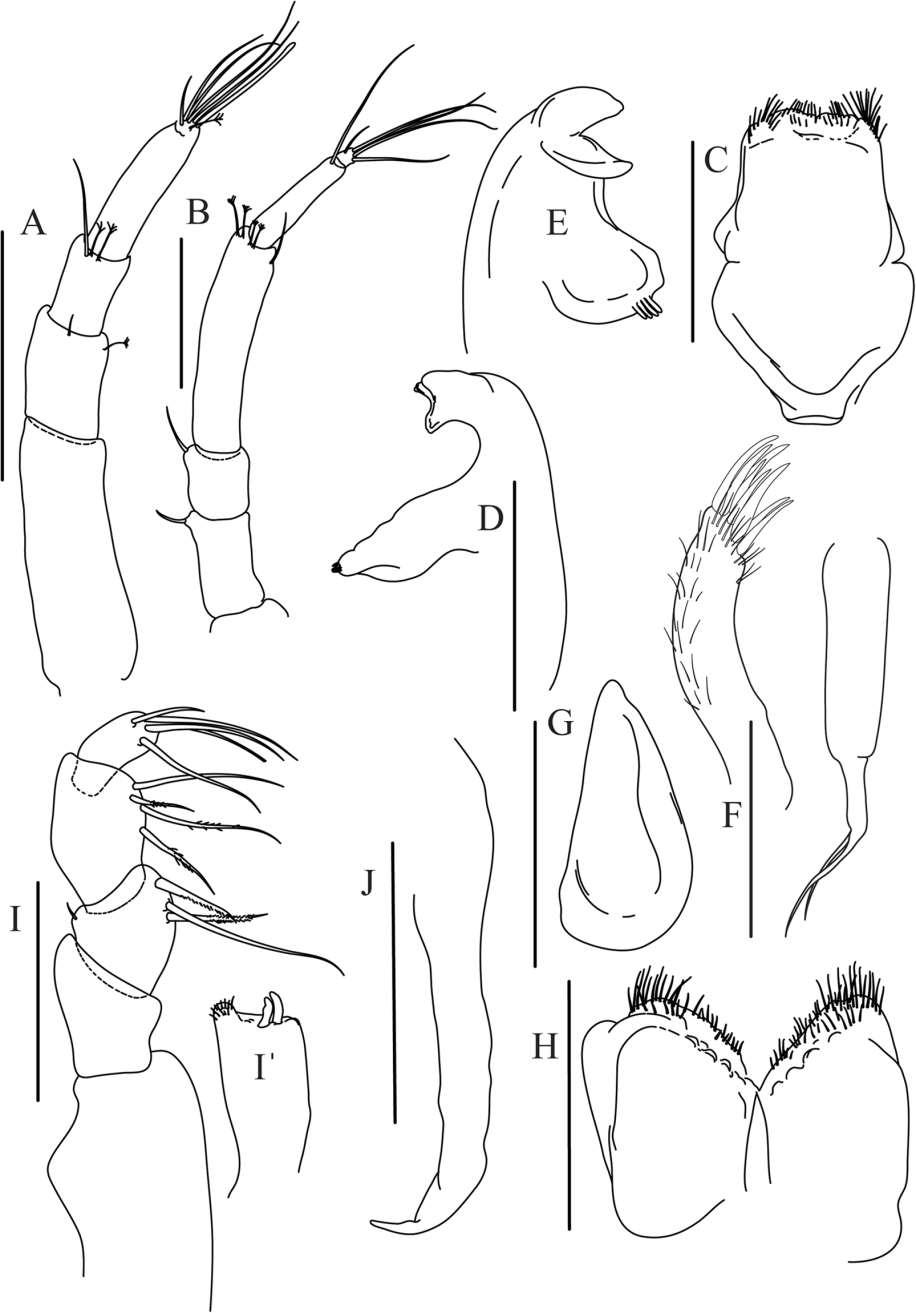
*Paranarthrurella caudata* (Kudinova-Pasternak, 1965), holotype, female (Mc-938) (**A**) Antennule. (**B**) Antenna. **(C**) Labrum. (**D**) Mandible, right. (**E**) Mandible, left. (**F**) Maxillule. (**G**) Maxilla. (**H**) Labium. (**I**) Maxilliped. (**I’**) Maxilliped endite. (**J)** Epignath. Scale 0.1 mm.

**Figure 6:**
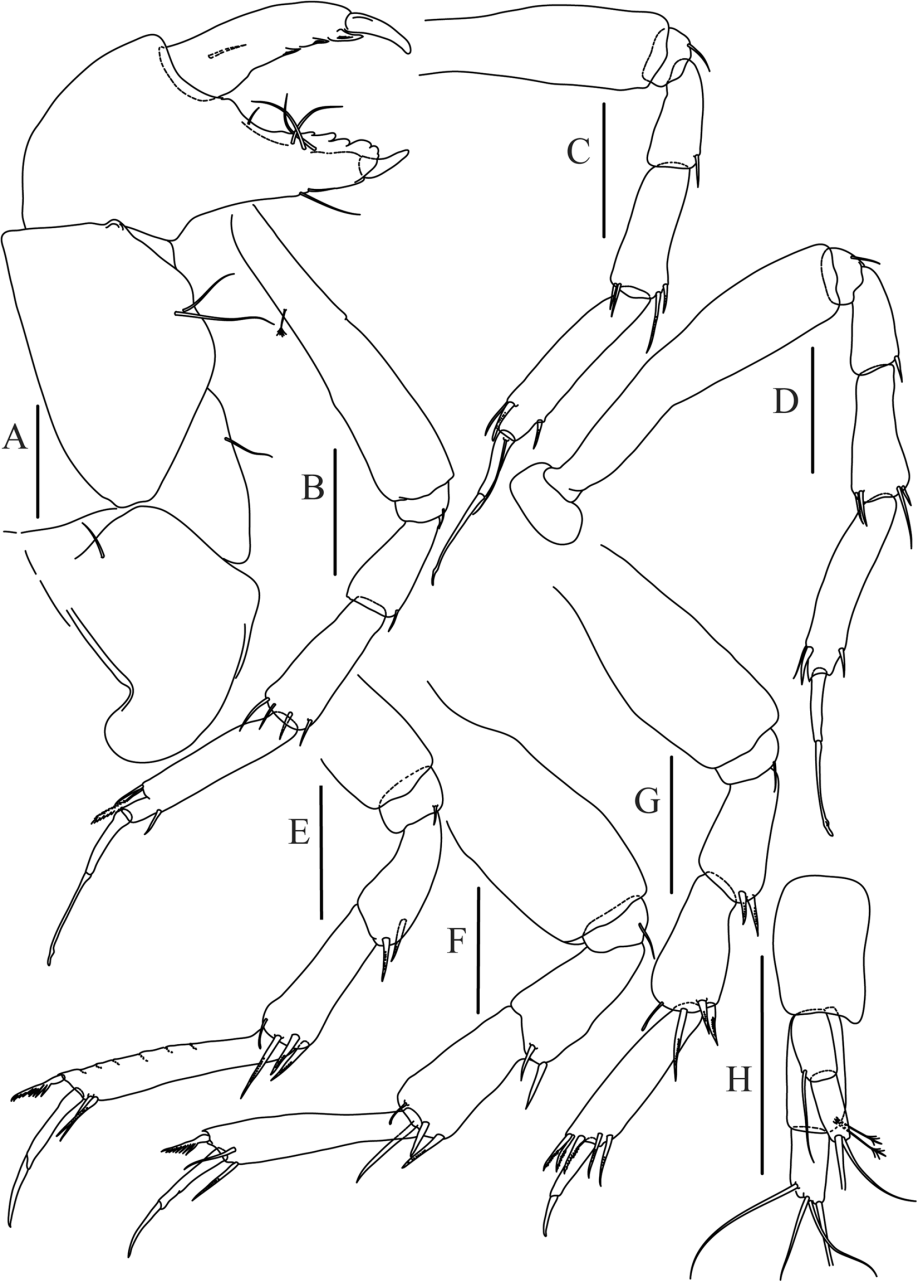
*Paranarthrurella caudata* (Kudinova-Pasternak, 1965), holotype, female (Mc-938) (**A**) Cheliped. (**B**–G) Pereopods 1–6, respectively. (**H**) Uropod. Scale 0.1 mm.

### Material examined

Holotype, neuter 3.0 mm BL (Mc 938), RV *Vityaz*, St. 3663, 6°13.4′S, 153° 43.7′E, 7974–8006 m, (coll. Birstein in 1957).

### Diagnosis

Female: Body long (>8 L:W). Pereonite-1 0.7 L:W. Pleonites lateral margin smooth. Pleonites without hyposphaenium. Pleotelson subrectangular, apex large. Cheliped carpus 1.6 L:W. Pereopod-1 merus with one spine. Pereopods 4–6 carpus with three spines. Pereopods 4–5 propodus dorsodistal spine strongly serrate. Pereopod-6 propodus dorsodistal spines weakly serrated. Uropod endopod 1.5x exopod.

**Description of neuter**, length 3.0 mm. Body (Fig. 4A,B) elongate, 9.0 L:W; cephalothorax 1.5 L:W, 2.1x pereonite-1. Pereonites 1−6: 0.7, 0.8, 0.9, 1.1, 1.1 and 0.7 L:W, respectively. Pereonites 2–5 about as long as wide, pereonites 1–3 wider proximally, pereonites 4–5 wider in midlength. Pleon 0.2 of total body length. All pleonites the same size, 0.3 times L:W. Pleotelson as long as four pleonites together, subrectangular in the dorsal view, apex large, pointed, directed backward.

Antennule (Fig. 5A) article-1 3.0 L:W, 2.3x article-2, no setae observed; article-2 1.5 L:W, 1.4x article-3, with one simple and one penicillate setae distally; article-3 1.4 L:W, 0.6x article-4, with one simple and two penicillate setae distally; article-4 3.3 L:W; article-5 vestigial, partly fused with article-4, with five simple and one penicillate setae, and aesthetasc distally.

Antenna (Fig. 5B) article-1 fused with body; article-2 1.8 L:W, 1.4x article-3, with distodorsal seta; article-3 1.1 L:W, 0.3 x article-4, with distodorsal seta; article-4 4.1 L:W, 1.8x article-5, with one simple and four penicillate setae distally; article-5 4.7 L:W, with distal seta; article-6 as long as wide, with four long and one short distal setae.

Mouthparts. Labrum (Fig. 5C) large, elongate; distally obtuse and with relatively sparse robust setae distally. Right mandible (Fig. 5D) incisor with smooth, weakly rounded edges, *lacinia mobilis* fused with incisor; molar distally elongated with four finger-shape setae. Left mandible (Fig. 5E) incisor distally simple; *lacinia mobilis* well developed with smooth edge; molar wide, with four finger-shape distal setae. Maxillule endite (Fig. 5F) with at least eight strong distal spines of various length and numerous setae distally and along outer margin; palp with two long simple setae. Maxilla (Fig. 5G) semitriangular. Labium (Fig. 5H) with two lobes; inner lobe with numerous robust setae on inner and distoinner margin; outer lobe small. Maxilliped (Fig. 5I) basis not observed; palp article-1 1.1 L:W, naked; article-2 1.1 L:W, with one long simple and two short serrate inner setae, and small outer seta; article-3 1.5 L:W, with one simple and three sparsely serrate inner setae; article-4 2.6 L:W, with four distal/subdistal inner setae (fifth seta not seen) and outer seta; maxilliped endites separated, narrow, 2.2 L:W, distally with two slender gustatory cusps; no distal seta observed. Epignath (Fig. 5J) distally pointed.

Cheliped (Fig. 6A) sclerite large semitriangular; basis 2.1 L:W, with one subdistal dorsal seta, posterior lobe small; merus wedge-shape, with ventral seta; carpus 1.6 L:W, with two ventral setae, no dorsal setae observed; chela slender, 1.2x carpus, 1.7 L:W; propodus (palm) with one seta near dactylus insertion, seta on inner side not seen; fixed finger with sharp distal spine (unguis), incisive margin well calcified, with five obtuse unequal teeth, and with three setae; fixed finger and dactylus unifacial; dactylus almost straight with three spines on inner margin and subproximal seta on inner side.

Pereopod-1 (Fig. 6B) longer than pereopods 2–3; basis 5.5 L:W, with distoproximal penicillate seta; ischium with ventral seta; merus 1.9 L:W, 0.7x carpus, with ventrodistal fine spine; carpus 3.0 L:W, 0.8x propodus, with four short distal spines; propodus 5.4 L:W, 2.7x dactylus, with one subdistal ventral spine, one fine and one long serrate dorsodistal spine; dactylus 6.7 L:W, 0.7x unguis, proximal seta not seen; unguis and dactylus about 0.9x propodus.

Pereopod-2 (Fig. 6C) basis broken during dissection; ischium with ventral seta; merus 2.5 L:W, 0.7x carpus, with ventrodistal spine; carpus 3.0 L:W, 0.75x propodus with four distal spine (ventrodistal spine longer); propodus 4.9 L:W and 2.9x dactylus, with small subdistal ventral spine, and two (one fine and one regular) subdistal dorsal spines; dactylus 6.1 L:W, 0.7x unguis, with seta reaching beyond dactylus.

Pereopod-3 (Fig. 6D) similar to pereopod-2, but dactylus seta not observed.

Pereopod-4 (Fig. 6E) basis broken; ischium with one ventral seta; merus 2.7 L:W, 0.9x carpus, with two ventrodistal serrate spines; carpus 3.6 L:W, 0.7x propodus, with three distal serrate spines and rod-like dorsodistal seta; propodus 6.2 L:W, with two ventrodistal serrate spines and strongly serrate dorsodistal spine; dactylus 7.1 L:W, 1.2x unguis; unguis and dactylus 0.8x propodus.

Pereopod-5 (Fig. 6F) as pereopod-4.

Pereopod-6 (Fig. 6G) similar to pereopod-5, but propodus 3.6 L:W, with three serrate dorsodistal spines; dactylus shorter, 5.5 L:W.

Uropod (Fig. 6H) exopod with two articles, 0.7x endopod, just longer than article-1 of endopod, article-1 1.9 L:W, with distal seta, article-2 2.8 L:W, with two subdistal penicillate setae and two simple distal setae; endopod with two articles, article-1 2.1 L:W; article-2 2.0 L:W, with at least four distal setae.

Male unknown.

### Distribution

The species is known only from the type locality - Bougainville Trench from 7974–8006 m depth.

### Remarks

*Paranarthrurella caudata* was described by Kudinova-Pasternak based on five specimens of length from 3.2 mm to 4 mm. From this collection, only the holotype (marked by Kudinova-Pasternak on the original label) exists and was available for our studies. For this reason, we took the advantage and partly dissected the specimen to redescribe this inadequately-described species.

*P. caudata* is the only member of the genus with a relatively slender, elongated and almost rectangular pleotelson and with three spines on carpus of the pereopods 4–6. Those two characters combined allow *P. caudata* to be distinguished from the other members of the genus, which have a rounded pleotelson (“onion-shape” in the description of Kudinova Pasternak^2^) and four spines on carpus of the pereopods 4–6. Moreover, the apex of the pleotelson in *P. caudata* is robust and directed backward, but small and directed downward in all other *Paranarthrurella* species.

Six species: *P. caudata*, *P. dissimilis, P. kizomba* sp. nov. *P. spinimaxillipeda, P. polonez* sp. nov. and *P. samba* sp. nov. (see below) are the only known species of the genus with elongated body habitus whose body is eight or more times longer than wide. Yet, none of them has a pleonite hyposphaenium (see remarks on page 6). *Paranarthrurella caudata* has strongly serrated dorsodistal seta on the propodus of pereopods 4–6. This character is present in a few members of the genus (described below), as well as in *P. dissimilis*, although the latter has four spines on carpus of pereopod 4–6, while *P. caudata* has only three.

***Paranarthrurella arctophylax*** **(Norman and Stebbing, 1886)**

Figures 7–9

**Figure 7:**
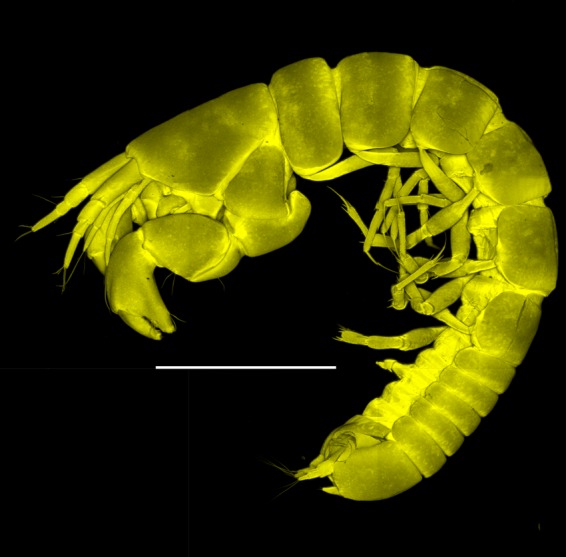
*Paranarthrurella arctophylax* (Norman and Stebbing, 1886), neuter (NI-39666) autofluorescence confocal image. Reconstructed maximum intensity projection of 3 x 3 area tile scan with Z-axis scan in two excitation/emission channels covering entire visible light spectrum. Scale 1 mm.

**Figure 8:**
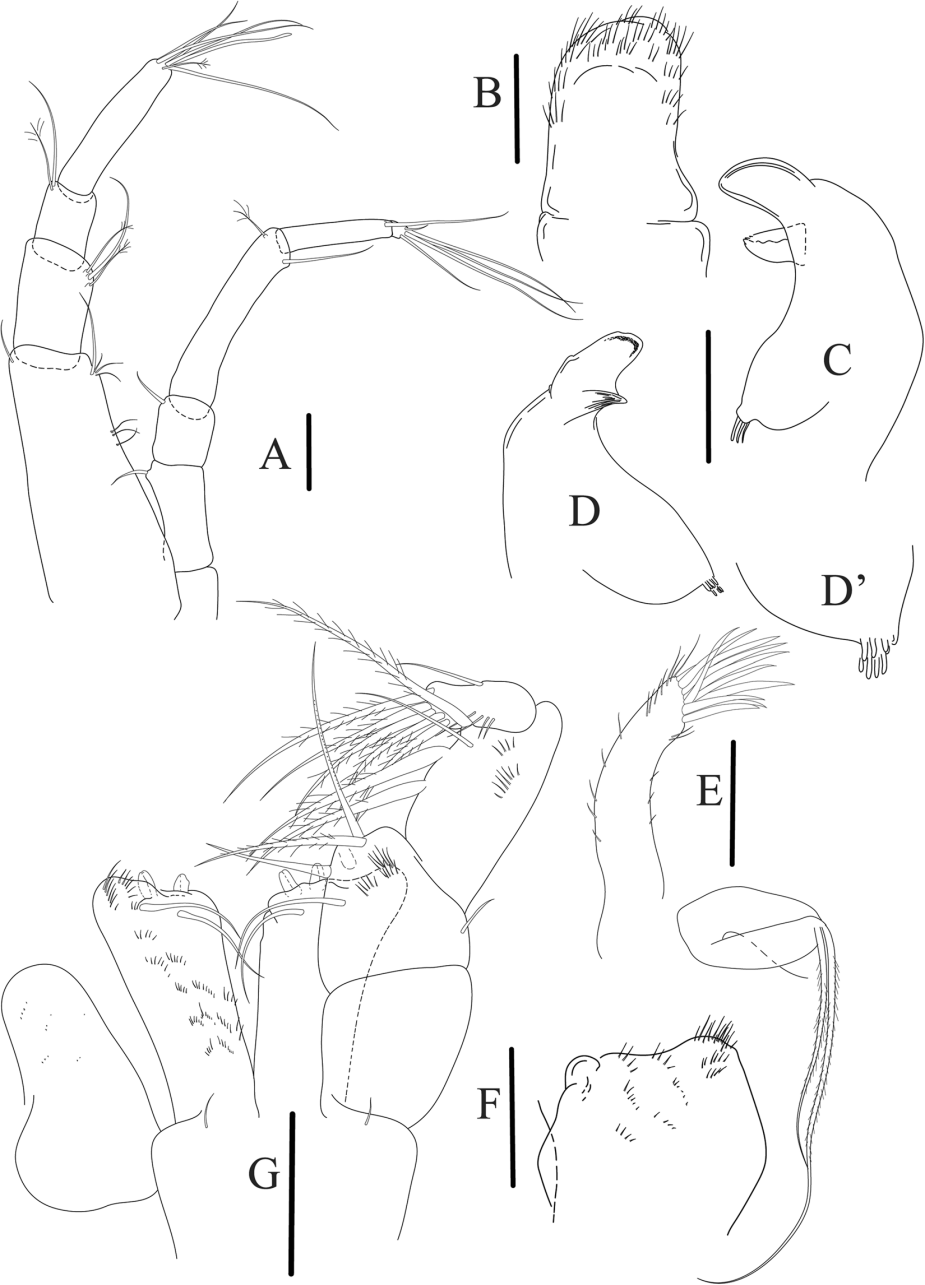
*Paranarthrurella arctophylax* (Norman and Stebbing, 1886), female (NI-39587) **(A**) Antennule and antenna. (**B**) Labrum. (**C**) Mandible, left. (**D**) Mandible, right. (**E**) Maxillule endite. (**F**) Labium. (**G**) Maxilla and maxilliped. Scale 0.1 mm.

**Figure 9:**
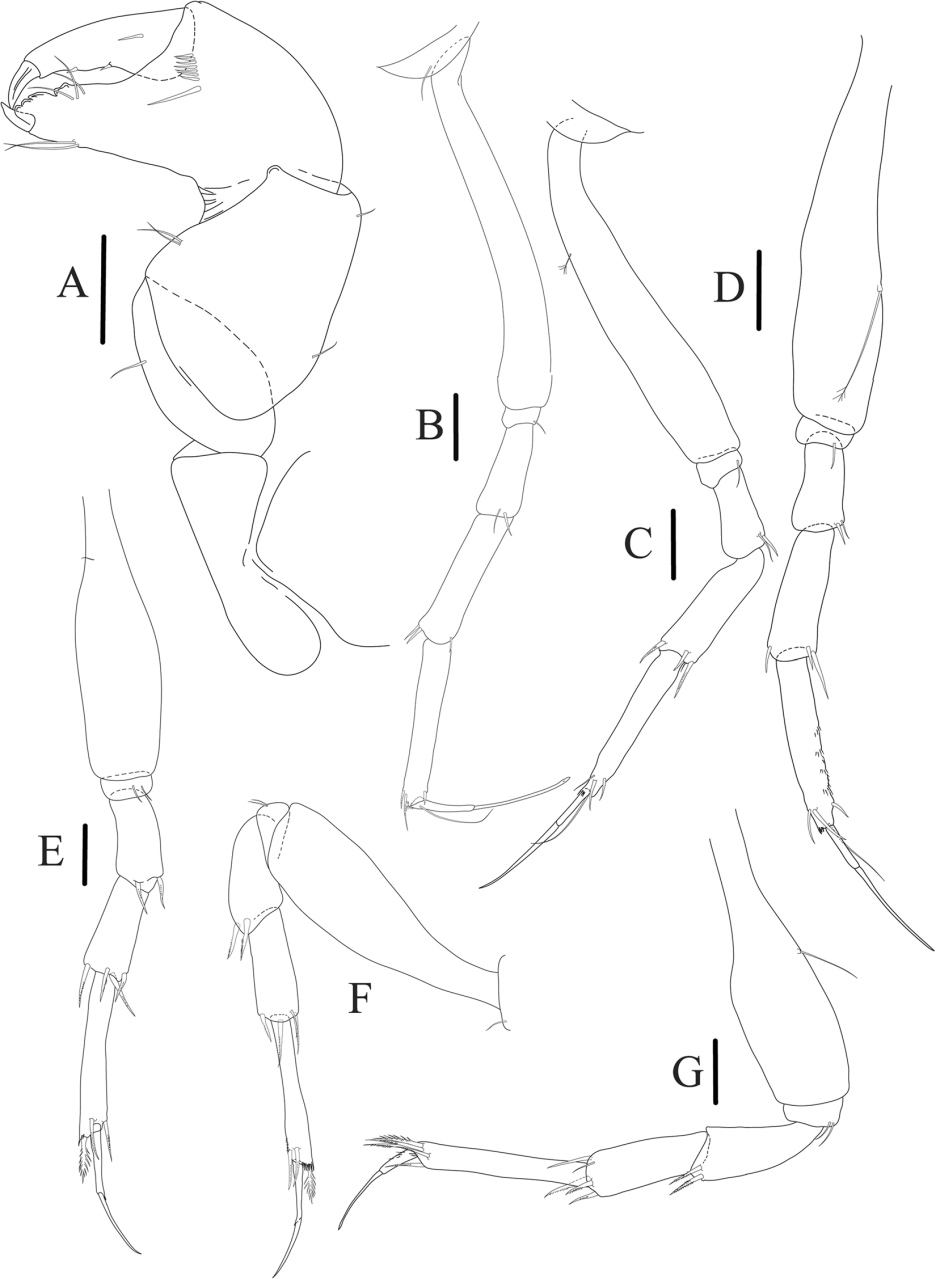
*Paranarthrurella arctophylax* (Norman and Stebbing, 1886), female (NI-39587) (**A**) Cheliped, outer side. (**B**–**G**) Pereopod 1–6, respectively. Scale 0.1 mm.

*Strongyrula arctophylax:* Norman and Stebbing, 1886:12, 110, 116–117, pl. 24 Fig. III^12^.

*Cryptocope arctophylax:* Hansen, 1913: 3, 106, 110–113, pl. 11 Fig. 2a–k ^8^.

*Leptognathia arctophylax:* Lang, 1971: 403^1^; Sieg, 1986: 168^73^.

*Paranarthrurella voeringi:* Jóźwiak *et al*., 2009: 59^16^.

### Material examined

*Strongylura arctophylax*, type (NHM 1903.5.20.8), 56°24′N, 11°19′W, between Ireland and Rockall, 2524 m.

*Paranarthrurella arctophylax*, ovigerous female (4.4 mm BL, dissected), (NI-39587), BIOICE St. 3176; neuter (non-ovigerous female) (4.3 mm BL), (NI-39666), BIOICE St. 2860; manca-3 (3.2 mm BL), (NI-39667), BIOICE St. 2863; neuter (non-ovigerous female) (4.4 mm BL), (NI-39668), BIOICE St. 2859.

*Paranarthrurella* cf. *arctophylax*, adult male (3.3 mm BL), (MCZ:IZ:47842), RV *Chain*, Cr. 106, St. 326.

### Diagnosis

Body short (<6.5 L:W). Pereonite-1 0.5 L:W. Pleonite 2 and 4 without hyposphaenium; pleonite-3 with large, pointed hyposphaenium. Cheliped carpus 1.3 L:W. Pereopods 4–6 carpus with four spines.

**Description of female**, length 4.0 mm. Body robust (Fig. 7), cephalothorax 2.3x pereonite-1. Pleon 0.3 of total body length. Pleonites 1 and 5 slightly longer than 2–4. Pleotelson just shorter than four pleonites together, rectangular in the dorsal view, apex large, pointed, directed backward.

Antennule (Fig. 8A) article-1 2.6 L:W, 2.3x article-2, with three penicillate setae at midlength and two simple and three penicillate setae distally; article-2 1.7 L:W, 1.7x article-3, with one simple and two penicillate setae distally; article-3 1.4 L:W, 0.5x article-4, with one simple and one penicillate setae distally; article-4 4.4 L:W, with five simple and one penicillate setae, and aesthetasc distally.

Antenna (Fig. 8A) article-1 fused with body; article-2 2.9 L:W, 1.9x article-3, with distal seta; article-3 1.2 L:W, 0.3x article-4, with distal seta; article-4 6.4 L:W, 1.7x article-5, with one simple and one penicillate setae distally; article-5 5.2 L:W, with distal seta; article-6 as long as wide, with four long and one short distal setae.

Mouthparts. Labrum (Fig. 8B) large, elongated; flattened and with setae distally. Left mandible (Fig. 8C) incisor distally simple; *lacinia mobilis* well developed with smooth edge; molar wide, with three palpate distal setae. Right mandible (Fig. 8D) incisor with smooth, gently rounded edges, *lacinia mobilis* fused with incisor; molar distally elongated with at least eight palpate setae (Fig. 8D’). Maxillule endite (Fig. 8E) with ten strong distal spines of various length and numerous setae distally and along outer margin; palp with two long plumose setae. Maxilla (Fig. 8G) semitriangular. Labium (Fig. 8F) with numerous setae on inner and distoinner margin and distoinner tubercle. Maxilliped (Fig. 8G) basis with short simple seta distally; palp article-1 1.1 L:W, naked; article-2 0.8 L:W, with two simple and two serrate inner setae, and one simple outer seta; article-3 1.7 L:W, with one simple and three plumose inner setae and some microtrichia; article-4 2.4 L:W, with five distal/subdistal plumose inner setae and one simple outer seta; maxilliped endites separated, narrow, 2.7 L:W, distally with two slender tubercles (gustatory cusps) and one pair of simple setae; microtrichia also present. Epignath not found.

Cheliped (Fig. 9A) basis 5.5 L:W, naked; sclerite large rectangular; merus wedge-shape, with ventral seta; carpus 1.3 L:W, with two ventral setae, and two dorsal setae observed; chela slender, 1.2x carpus, 1.6 L:W; propodus (palm) with one long and seven setae on inner side; fixed finger with sharp distal spine (unguis), incisive margin well calcified, divided by transversal grooves into processes, and with three setae, and two setae ventrally; fixed finger and dactylus unifacial; dactylus with two spines on inner margin and subproximal seta on inner side.

Pereopod-1 (Fig. 9B) coxa with seta; basis 8.8 L:W, naked; ischium with ventral seta; merus 3.0 L:W, 1.4x carpus, with two ventrodistal setae; carpus 4.3 L:W, 0.8x propodus, with two short dorsodistal setae and one short ventrodistal seta; propodus 7.2 L:W, 2.8x dactylus, with one subdistal ventral seta, one short and one long simple dorsodistal setae; dactylus 8.7 L:W, 0.6x unguis, with proximal seta.

Pereopod-2 (Fig. 9C) basis 6.1 L:W, with dorsoproximal penicillate seta; ischium with ventral seta; merus 2.2 L:W, 0.7x carpus, with two ventrodistal setae; carpus 3.4 L:W, 0.75x propodus, with one simple and three serrate distal setae; propodus 7.2 L:W and 3.6x dactylus, with small subdistal ventral spine, seta and spine dorsally; dactylus 9.1 L:W, 0.4x unguis, with seta reaching over dactylus.

Pereopod-3 (Fig. 9D) similar to pereopod-2, but basis with long penicillate seta ventrally.

Pereopod-4 (Fig. 9E) basis 3.5 L:W, with short dorsal seta; ischium with two ventral setae; merus 2.3 L:W, 1.1x carpus, with two ventrodistal serrate spines; carpus 2.2 L:W, 0.6x propodus, with four distal serrate spines; propodus 7.2 L:W, with two ventrodistal serrate spines and strongly serrate (spinose) dorsodistal spine; dactylus 1.0 L:W, 0.9x unguis; unguis and dactylus 0.9x propodus.

Pereopod-5 (Fig. 9F) as pereopod-4, but no seta on basis.

Pereopod-6 (Fig. 9G) similar to pereopod-4, but carpus with additional simple seta distally; propodus with addistional serrate seta; dactylus shorter, 4.2 L:W

### Distribution

The species was described for the NE Atlantic based on one specimen dredged during the Porcupine expedition at 2524 m depth between Ireland and Rockall. Later on, during the Ingolf expedition, new records in the David Strait and subsequently during BIOGAS expeditions in the northern Bay of Biscay, extended its depth range between 1970 and 4720 m (Table 5, Fig. 39). The species has been also collected in the Iceland Basin during BIOICE cruises, and it has been also recorded in the Ibero-Moroccan Gulf (Bird, pers. comm.).

### Remarks

*Paranarthrurella arctophylax* is the only *Paranarthrurella* species in the North Atlantic that has well developed hyposphaenium only on pleonite-3^80^. Another species that also occurs at higher latitude in the North Atlantic is *P. voeringi*, whose hyposphenia is present also in pleonite 2 and 4 as well. Those two taxa have distinct temperature preferences conditioning their geographical distribution (see Table 5; also remarks under *Paranarthrurella* sp.2). The individual collected during *Chain* Cr. 106, is a fully mature male, so certain identification is not possible. However, because this individual was collected in the distributional area where *P. arctophylax* occurs^81^, we anticipate it probably represents this species, until abiotic data could corroborate it.

**Table 5:** Localities and abiotic characteristic of the stations for the northern *Paranarthrurella* species distributed in the North Atlantic. – data not available.

Plotted station	Expedition Station	Date	Location	Depth (m)	Temperature (ºC)	Salinity (pps)	Sediment	Source
***P. arctophylax***								
1	Porcupine_St 30	1869	Rockall Basin	2523.7	2.83	—	—	Norman and Stebbing^12^
2	Ingolf_St 24	25/06/1895	Davis Strait	2258	2.4	35.04	gray deep-sea clay	Knudsen^108^; Boeggild^109^; Hansen^8^
3	Ingolf_St 36	28/07/1895	Davis Strait	2702	1.5	34.93	transition clay	Knudsen^108^; Boeggild^109^; Hansen^8^
4	BIOGAS_St 1	1973‒1980	Bay of Biscay	1970‒2227	3.28	34.99	silt	Holdich and Bird^81^, Vangrieshem^107^, Auffret^110^
5	BIOGAS_St 2	1973‒1980	Bay of Biscay	3250‒3548	2.67	34.96	silt‒very find sand	Holdich and Bird^81^, Vangrieshem^107^, Auffret^110^
6	BIOGAS_St 3	1973‒1974	Bay of Biscay	3992‒4260	2.13	34.92	silt	Holdich and Bird^81^, Vangrieshem^107^, Auffret^110^
7	BIOGAS_St 4	1974	Bay of Biscay	4720‒4721	2.12	34.89	silt‒very find sand	Holdich and Bird^81^, Vangrieshem^107^, Auffret^110^
8	BIOICE_St 2859	29/08/1995	Iceland Basin	2270‒2271	2.37	34.95	silty sand	https://en.ni.is/node/27982
9	BIOICE_St 2860	30/08/1995	Iceland Basin	2295‒2298	2.60	34.96	silty sand	https://en.ni.is/node/27982
10	BIOICE_St 2863	30/08/1995	Iceland Basin	2400‒2400	2.07	34.95	sand, shell fragments	https://en.ni.is/node/27982
11	BIOICE_St 3176	29/07/2000	Iceland Basin	2537‒2546	—	—	—	https://en.ni.is/node/27982
***P****.* **cf**. ***arctophylax***								
12	Chain106_St 326	22/08/1972	Porcupine Seabight	3859	—	—	—	—
***P. voeringi***								
13	Norske Nordhavs_St 31	29/06/1876	Storeggen Bank	763	−1.0	—	sabulous clay	Sars G.O.^61^
14	Norske Nordhavs_St 124	19/06/1877	off Helgeland	640	−0.9	—	coarse clay	Sars G.O.^61^
15	Norske Nordhavs_St 248	08/08/1877	off Helgeland	1423	−1.4	—	bioculina clay	Sars G.O.^61^
16	Ingolf_St 116	23/07/1896	S of Jan Mayen	699	−0.4	—	transition clay	Knudsen^108^; Boeggild^109^; Hansen^8^
17	Ingolf_St 117	23/07/1896	S of Jan Mayen	1889	−1.0	35.00	globigerina clay	Knudsen^108^; Boeggild^109^; Hansen^8^
18	Ingolf_St 138	10/08/1896	NW Faeroes	887	−0.6	35.22	transition deep-sea clay	Knudsen^108^; Boeggild^109^; Hansen^8^
19	Vicking_KGS1	23/05/2006	Storega, off shore Norway	742	−0.7	33.40	soft-bottom	Błażewicz-Paszkowycz and Bamber^83^, Olu pers. comm.
20	PS66_St 103-1	01/07/2004	Hausgarten, E Fram Strait	1300	−0.88	34.91	silt	Jóźwiak *et al*.^16^, Budéus^111^
21	PS72_St 137-5	12/07/2008	Hausgarten, E Fram Strait	1273	−0.84	34.92	silt	Jóźwiak *et al*.^16^, Beszczynska-Möller & Wisotzki^112^
***Paranarthrurella*** **sp.1**								
22	IceAGE1_St 1155	17/09/2011	NE Iceland, Norwegian Sea	2203.8‒2173.9	−0.75	34.91	very find sand	Brix *et al*.^113,114^
***Paranarthrurella*** **sp.2**								
23	IceAGE1_St 1155	17/09/2011	NE Iceland, Norwegian Sea	2203.8‒2173.9	−0.75	34.91	very find sand	Brix *et al*.^113,114^
24	IceAGE1_St 1191	21/09/2011	NE Iceland, Norwegian Sea	1574.7‒1581.1	−0.74	34.91	silt	Brix et al.^113,114^

***Paranarthrurella corroboree*** **Błażewicz and Jóźwiak, sp. nov.**

Figures 10–13

**Figure 10:**
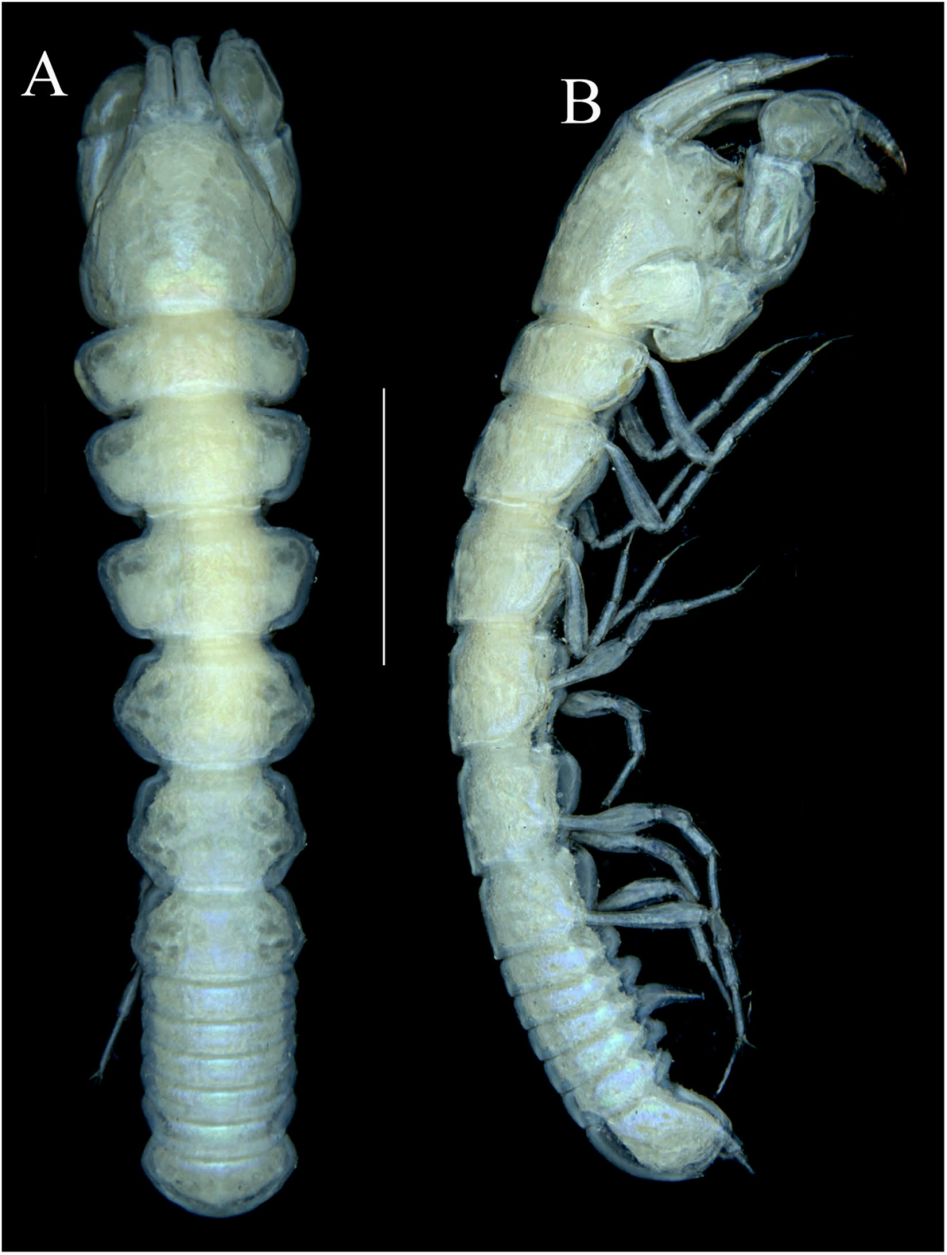
*Paranarthrurella corroboree* sp. nov., holotype, female (J57829) (**A**) Dorsal. (**B**) Lateral. Scale 1 mm.

**Figure 11:**
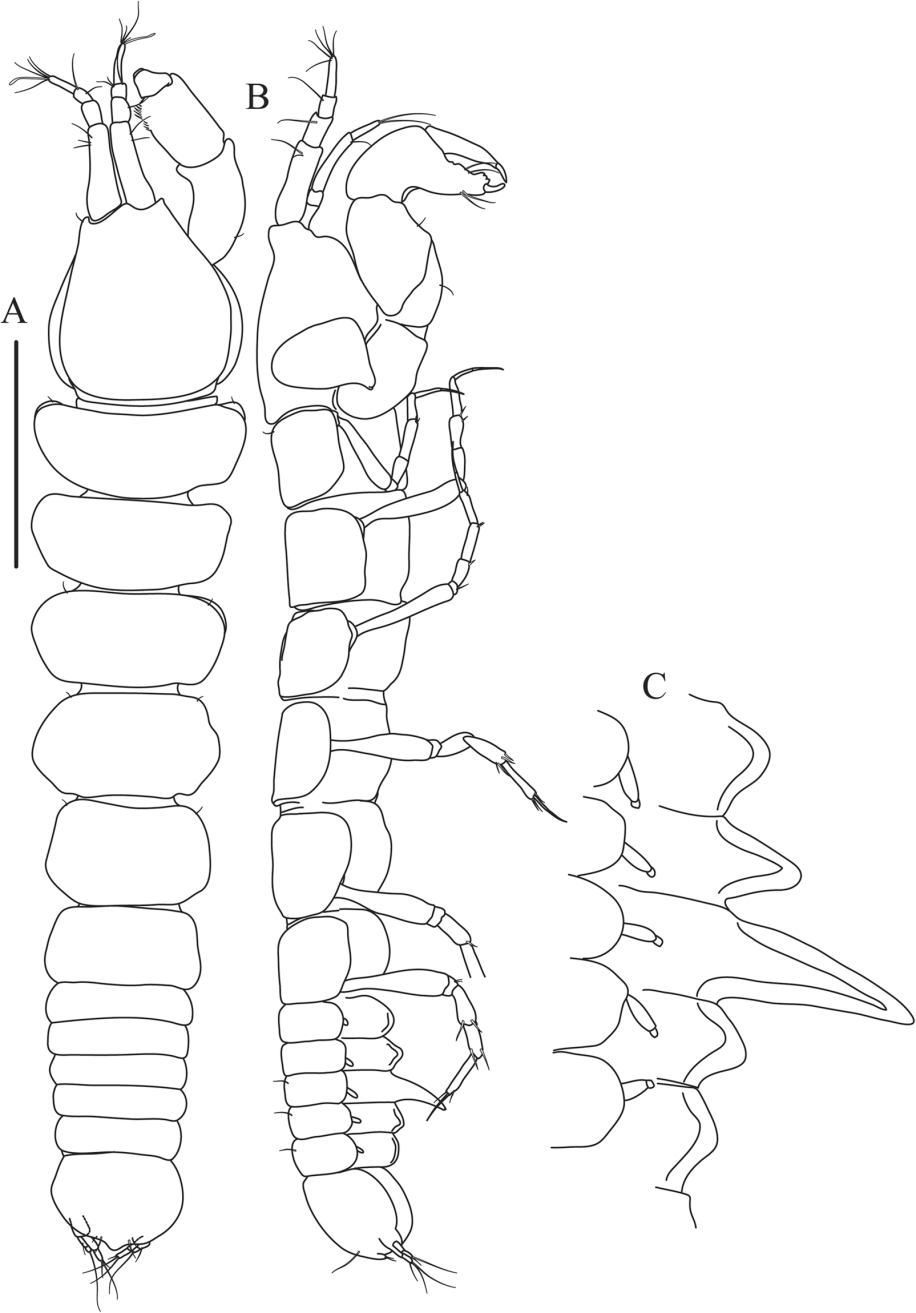
*Paranarthrurella corroboree* sp. nov., paratype, female (J61551) (**A**) Dorsal. (**B**) Lateral. (**C**) Pleon lateral. Scale 1 mm.

### Material examined

Holotype ovigerous female (6.2 mm BL), (J57829), SLOPE St. 25.

Paratypes: neuter (5.7 mm BL), (J61550), SLOPE St. 140; juvenile male, 5.0 mm BL, manca-3, (2.5 mm BL), (J61552), SLOPE St. 142; female dissected on slides, (5.8 mm BL) (J61551), SLOPE St. 170.

urn:lsid:zoobank.org:act:903EFAA4–8807–47FC-BEF3-2235A7A03EC7.

### Diagnosis

Female: Body short (<6.5 L:W). Pereonite-1 0.3 L:W. Pleonites lateral margin smooth. Pleonite-2 with small and pleonite-3 with big, weakly curved backward hyposphaenium. Pleotelson rounded and swollen, apex small. Cheliped carpus 1.4 L:W. Pereopod-1 merus with two spines. Pereopods 4–6 carpus with four spines. Pereopods 4–6 propodus dorsodistal spine strongly serrate. Uropod endopod 1.5x exopod.

### Etymology

In Aboriginal language ‘corroboree’ is an event during which the particular interaction with the Dreamtime is reached through dance and music. Noun in apposition.

**Description of neuter**, length 4.53 mm. Body (Fig. 11A,B) robust, 5.2 L:W; cephalothorax 1.4 L:W, 2.35x pereonite-1. All pereonites longer than wide. Pereonites 1−6: 0.3, 0.5, 0.5, 0.5, 0.6 and 0.5 L:W, respectively; all pereonites wider than long; pereonites 1–3 wider proximally, pereonites 4–5 wider in midlength. Pleon 0.25x of total body length. All pleonites the same size, 1.6 W:L. Pleonite-3 with large triangular hyposphaenium, weakly curved backward, pleonite-2 with small, but wide hyposphaenium (Fig. 11C). Pleotelson 3.5x pleonite-6.

**Figure 12:**
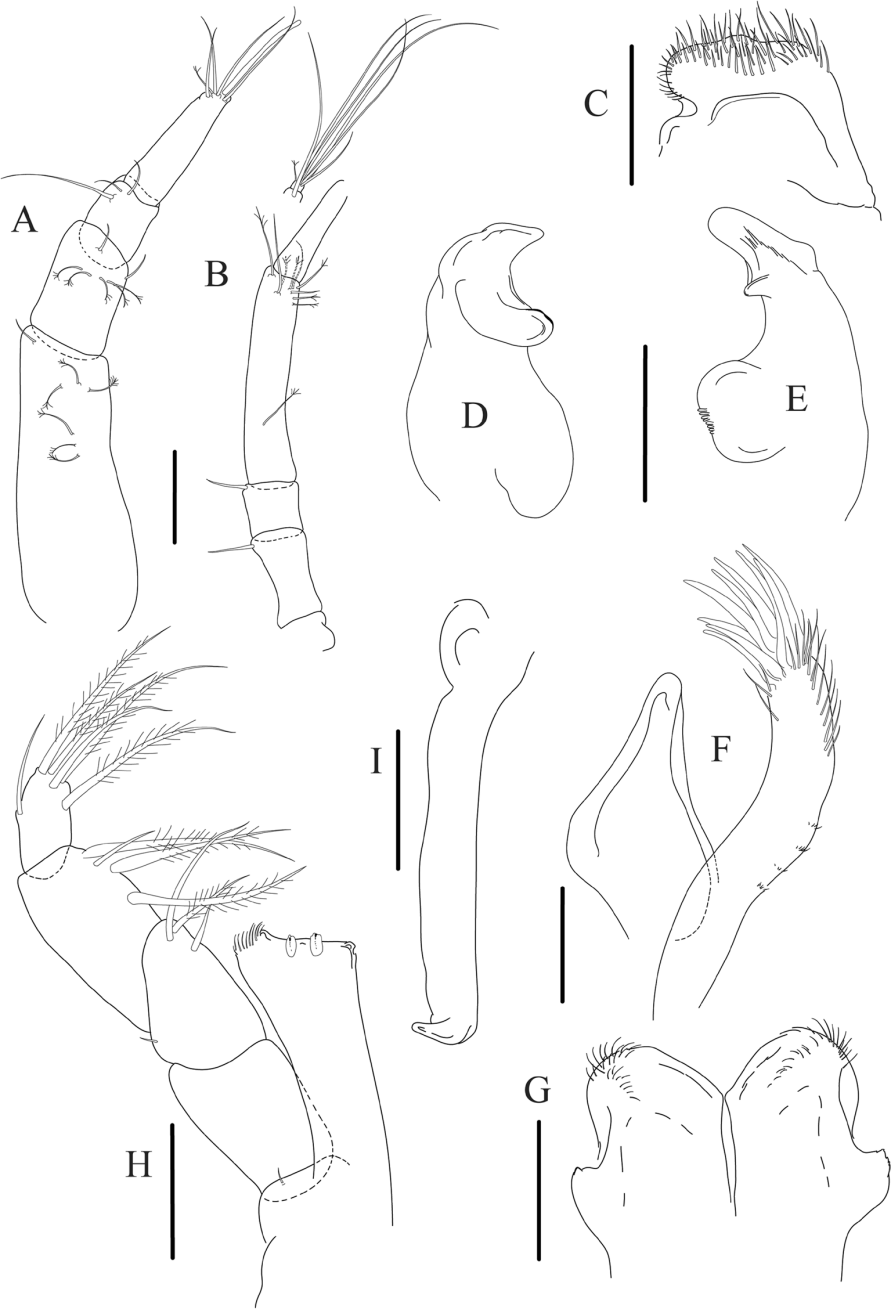
*Paranarthrurella corroboree* sp. nov., paratype, female (J61551) (**A**) Antennule. (**B**) Antenna. (**C**) Labrum. (**D**) Mandible, left. (**E)** Mandible, right. (**F**) Maxillule and maxilla. (**G**) Labium. (**H**) Maxilliped. (**I**) Epignath. Scale 0.1 mm.

Antennule (Fig. 12A) article-1 2.8 L:W, 2.4x article-2, with three penicillate setae at midlength, one simple and three penicillate setae distally; article-2 1.5 L:W, 1.5x article-3, with two simple and five penicillate setae distally; article-3 1.3 L:W, 0.6x article-4, with two short simple and one long setae distally; article-4 3.8 L:W; article-5 almost completely fused with article-4, with four simple, one penicillate setae and one aesthetasc distally.

Antenna (Fig. 12B) 0.8 times length of antennule; article-1 fused with body; article-2 2.2 L:W, 1.5x article-3, with distodorsal seta; article-3 1.2 L:W, 0.3x article-4, with distodorsal seta; article-4 5.1 L:W, 1.8x article-5, with penicillate seta at midlength, three simple and six penicillate setae distally; article-5 five L:W, with distal seta; article-6 minute, with one penicillate and five distal setae.

Mouthparts. Labrum (Fig. 12C) hood-shape, distally setose. Left mandible (Fig. 12D) incisor pointed, *lacinia mobilis* rounded, margins simple; molar process distally broad and rounded. Right mandible (Fig. 12E) incisor margin simple, distally rounded, *lacinia mobilis* in shape of small protrusion; molar process broad and rounded distally with row of seven short finger-shape spines. Maxillule endite (Fig. 12F) with nine spines, distal and dorsodistal margin setulose. Maxilla (Fig. 12F) triangular, naked. Labium (Fig. 12G) bilobed; inner lobe with numerous, short setae along distal margin; outer lobe, vestigial. Maxilliped (Fig. 12H) basis partly broken during dissection, with minute distal seta; palp article-1 1.4 L:W, naked; article-2 1.6 L:W, with three inner setae (one simple and two plumose) and one outer seta; article-3 1.6 L:W, with four inner setae (one simple and three plumose); article-4 2.1 L:W, with five plumose setae distally and one simple subdistal seta; maxilliped endites separated, with pair of slender gustatory cusps, distouter corner setulated. Epignath (Fig. 12I) distally pointed, naked.

**Figure 13:**
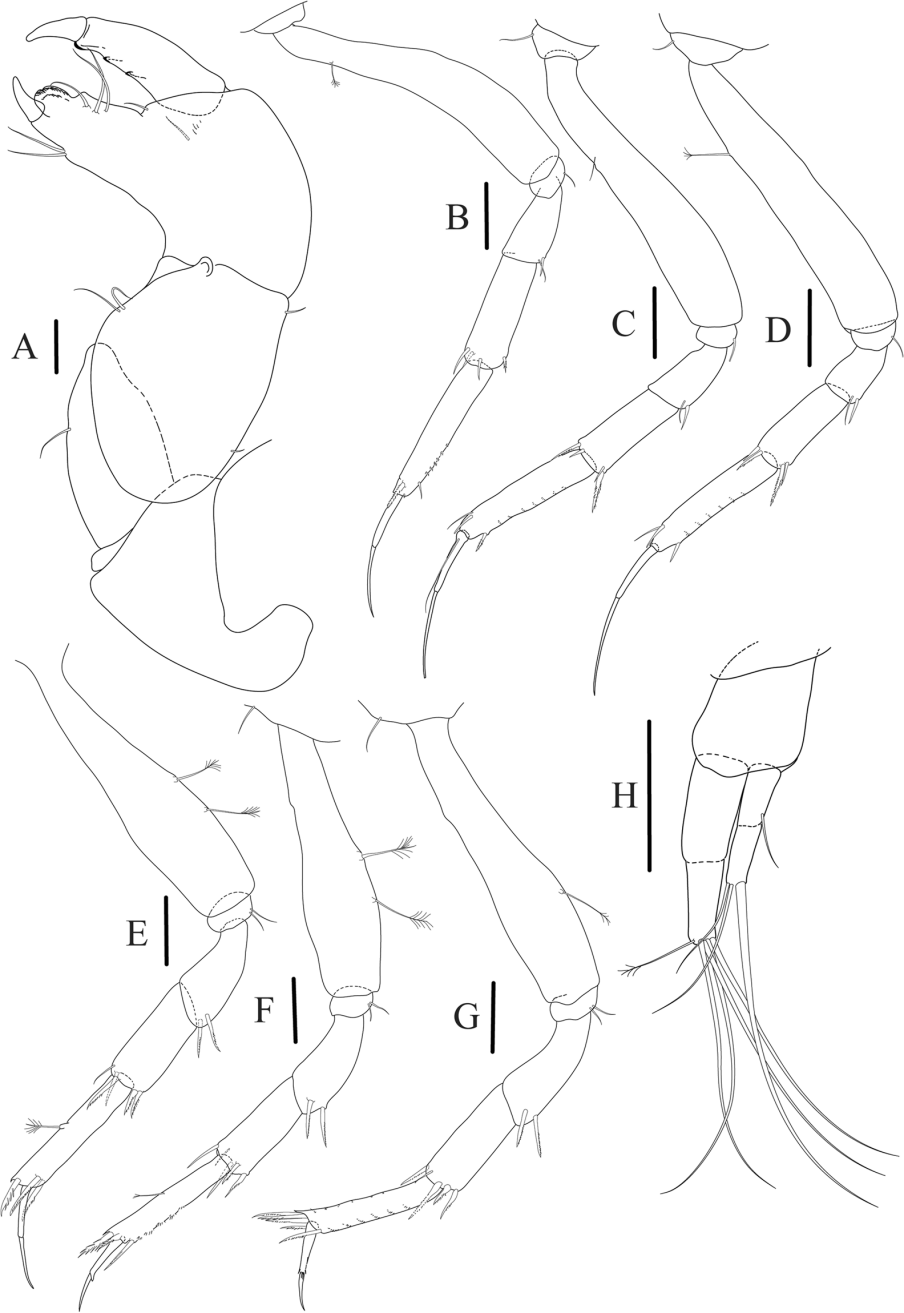
*Paranarthrurella corroboree* sp. nov., paratype, female (J61551) (**A**) Cheliped. (**B**–**G**) Pereopod 1–6, respectively. (**H**) Uropod. Scale 0.1 mm.

Cheliped (Fig. 13A) sclerite large triangular; basis 1.3 L:W; merus ventral margin longer than that of carpus, with ventral seta; carpus wider medially, 1.4 L:W, with two ventral setae and one distal and one subproximal setae on dorsal margin; chela 1.2x carpus, propodus (palm) with seta near dactylus insertion, one longer and four short setae on inner side; fixed finger with two simple setae ventrally and three setae on cutting edge, incisive margin well calcified, distally with large protrusion; fixed finger and dactylus unifacial; dactylus weakly bent downward, with three blunt spines on cutting margin.

Pereopod-1 (Fig. 13B) basis 6.1 L:W, with dorsoproximal penicillate seta; ischium with ventral seta; merus 1.8 L:W, 0.7x carpus, with fine and regular spine; carpus 3.0 L:W, 0.7x propodus, with one serrate and three simple spines distally; propodus 5.7 L:W, 2.6x dactylus, with one seta and one serrate spine subdistally, ventral margin with microtrichia; dactylus 0.8x unguis; unguis and dactylus about 0.8x propodus.

Pereopod-2 (Fig. 13C) coxa with seta; basis 5.8 L:W, with penicillate seta dorsally; ischium with ventral seta; merus 1.9 L:W, with fine and regular spine; carpus 2.8 L:W, 0.7x propodus, with one simple and one serrate spines and one large serrate and one small simple ventrodistal spines; propodus 4.6 L:W and 2.1x dactylus, with subdistal dorsal fine and regular spines and serrate ventrodistal spine, ventral margin with microtrichia; dactylus 0.7x unguis, with one proximal seta; dactylus and unguis 1.1x propodus.

Pereopod-3 (Fig. 13D) similar to pereopod-2, dactylus seta not observed.

Pereopod-4 (Fig. 13E) basis 4.4 L:W, with two penicillate ventral setae; ischium with two ventral setae; merus 2.2 L:W, 0.9x carpus, with two ventrodistal serrate spines; carpus 2.4 L:W, 0.9x propodus, with simple dorsodistal seta and with four distal spines; propodus 5.7 L:W, with one penicillate dorsal seta, two ventrodistal serrate spines and strongly serrate dorsodistal spine; dactylus 0.9x unguis; dactylus and unguis 0.7x propodus.

Pereopod-5 (Fig. 13F) as pereopod-4.

Pereopod-6 (Fig. 13G) similar to pereopod-5, but basis with only one penicillate seta, propodus with two ventrodistal serrate spines and three dorsodistal spines.

Uropod (Fig. 13H) basal article 1.4 L:W; exopod with two articles, 0.6x endopod, article-1 2.3 L:W, with simple seta, article-2 2.6 L:W, with three distal setae; endopod with two articles, article-1 2.2 L:W, naked; article-2 2.6 L:W, with one penicillate and four simple distal setae.

### Distribution

*Paranarthrurella corroboree* sp. nov. is known from SE Australia (Bass Strait slope) from depths of 1450–1975 m.

### Remarks

*Paranarthrurella corroboree*, is one of four species (see below) with a short body habitus and well developed hyposphaenium on pleonite-3 and small hyposphaenium on pleonite-2. Two other species with large hyposphaenium on pleonite-3 are: *P. voeringi* that has also small on pleonite-4, and *P. moonwalk* n. sp (see below) whose hyposphaenium on pleonite-3 is directed backward. Additionally, *P. corroboree* lacks a projection on the inner margin of the cheliped dactylus that is well developed in *P. voeringi*^16^ and has a slender endopod in uropod that is about four times as long as wide (endopod uropod is only three times as long as wide in *P. moonwalk*). Another species with a hyposphaenium on pleonite-3 is *P. arctophylax*, but it lacks hyposphaenia on pleonites 2 and 4.

***Paranarthrurella dissimilis*** **(Lang, 1972)**

For synonyms see Jóźwiak *et al*.^16^.

***Diagnosis*** (amended)^16^. Body long (>8 L:W). Pleonite 2–4 without hyposphaenia. Cheliped carpus 1.4 L:W. Pereopod-1 merus with two spines. Pereopods 4–6 carpus with four spines. Pereopods 4–6 propodus dorsodistal spine strongly serrate. Uropod endopod 1.3x exopod.

### Remarks

From six species of *Paranarthrurella* which have a long body, *P. dissimilis* can be distinguished by: a rounded pleotelson with a small, pointed downward apex (large and pointed backward in *P. caudata*); smooth lateral margins of pleonites (pointed in *P. polonez*); by presence of strongly serrated spine on the propodus of pereopods 4–6; *P. spinimaxillipeda*, *P. samba* and P*. kizomba* have weakly serrated spine at this location.

### Distribution

The species is known only from the type locality in the Sargasso Sea (NW Atlantic), at 6000 m depth.

***Paranarthrurella kizomba*** **Błażewicz and Frutos sp. nov.**

Figures 14 and 15

**Figure 14:**
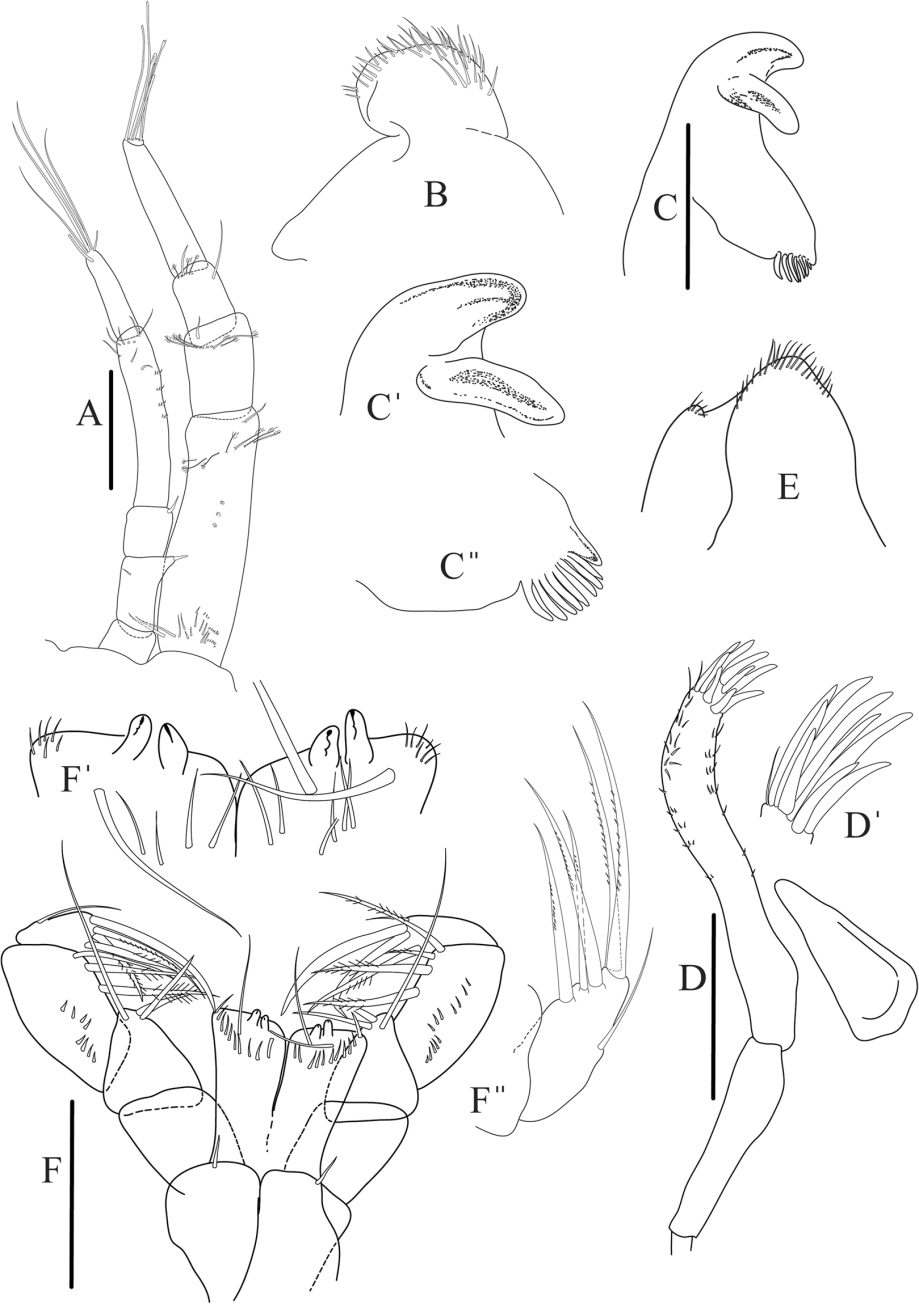
*Paranarthrurella kizomba* sp. nov., holotype neuter (ZMH K-55987) (**A)** Antennule and antenna. (**B**) Labrum. (**C**) Mandible, left. (**C’**) mandible left incisor details. (**C”**) Mandible left, molar details (**D**) Maxillule and maxilla. (**D’**) Maxillule endite details. (**E**) Labium. **(F**) Maxilliped. (**F’**) Maxilliped endites, details. (**F”**) Maxilliped, palp article-4 details. Scale 0.1 mm.

**Figure 15:**
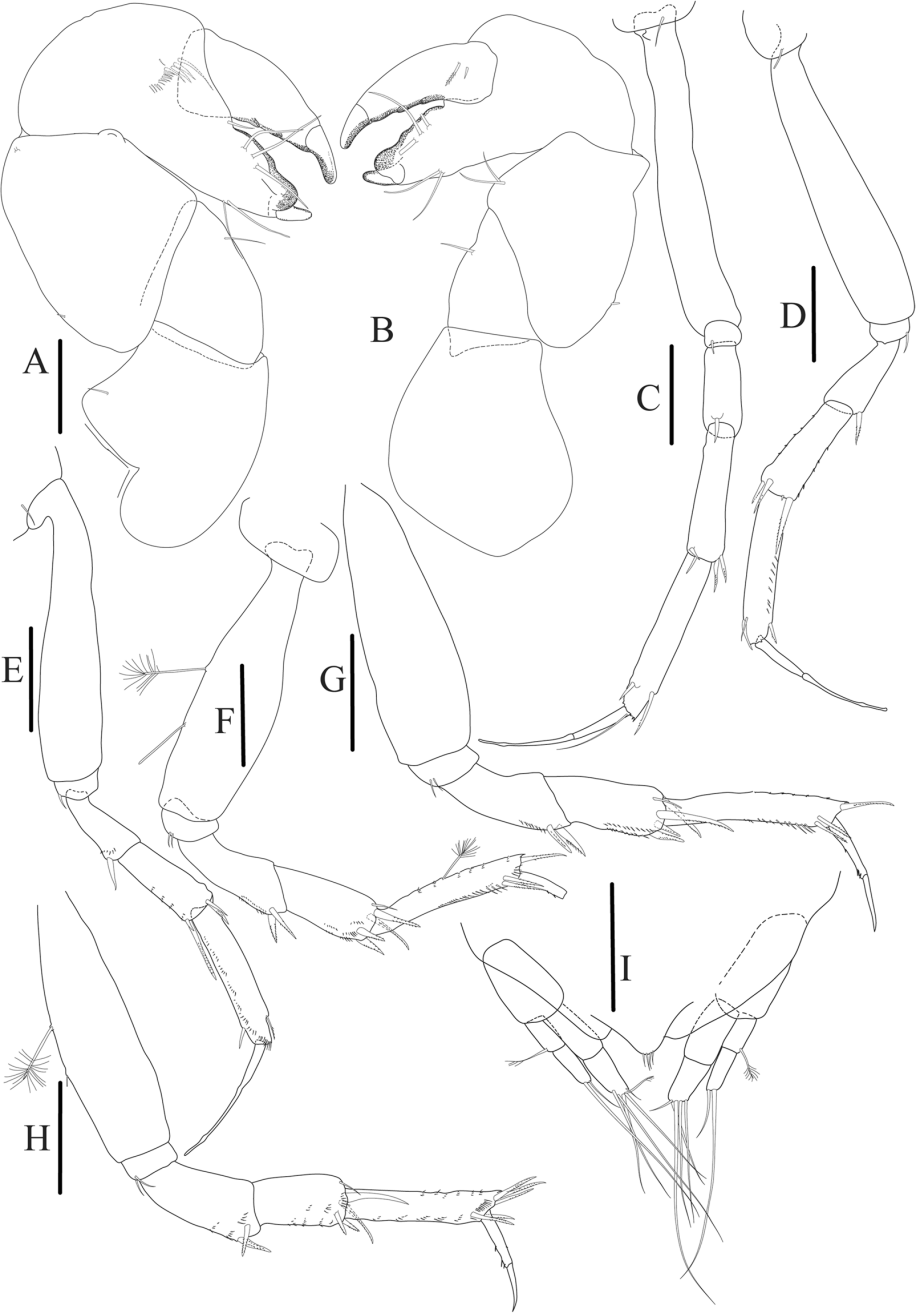
*Paranarthrurella kizomba* sp. nov., holotype neuter (ZMH K-55987) (**A**) Cheliped, outer side. (**B**) Cheliped, inner side. (**C**–**H**) Pereopod 1–6, respectively. **(I**) Uropod. Scale 0.1 mm.

### Material examined

Holotype neuter (3.1 mm BL), (ZMH K-55987), RV *Sonne*, SO-237, Vema-TRANSIT, St. 6–8.

### Diagnosis

#### Female

Body elongate. Pleonites lateral margin smooth. Pleonites 2–4 without hyposphaenium. Pleon rounded and swollen, apex small. Cheliped carpus 1.3 L:W. Pereopod-1 merus with one spine. Pereopods 4–6 carpus with four spines. Pereopods 4–6 propodus dorsodistal spine finely serrate. Uropod endopod 1.6x exopod.

### Etymology

The species name is given for a dance with African origin in Angola. Noun in apposition.

**Description of neuter**, length 3.1 mm. Body elongate (not illustrated because of poor condition), 9.6 L:W; cephalothorax about 1.5 L:W, 1.7x pereonite-1. Pleon 0.2x total body length. All pleonites of the same size. Pleotelson 4.0x pleonite-5, swollen, apex small, directed downward.

Antennule (Fig. 14A) article-1 3.5 L:W, 2.5x article-2, with three middle penicillate setae (broken), seven penicillate setae arranged in vertical row to main axis of the article and simple distal seta; article-2 1.5 L:W, 1.3x article-3, with nine penicillate distal setae arranged in vertical row to main axis of the article and short distal simple seta; article-3 1.7 L:W, 0.5x article-4, with two simple and three penicillate setae distally; article-4 4.2 L:W; article-5 vestigial, with five simple distal setae and one aesthetasc.

Antenna (Fig. 14A) 0.7 times length of antennule; article-1 fused with body; article-2 1.8 L:W, 1.4x article-3, with short distodorsal seta, situated on apophysis in right angle to axis of the article; article-3 1.7 L:W, 1.3x article-4, with short distodorsal seta situated in right angle to axis of the article; article-4 5.5 L:W, 2.0x article-5, with microtrichia on dorsal margin, and at least seven short distal and subdistal setae; article-5 4.3 L:W, with distal seta; article-6 as long as wide, with four long and one short distal setae.

Mouthparts. Labrum (Fig. 14B) distally rounded and with relatively sparse and long setae distally. Left mandible (Fig. 14C,C’) incisor with smooth margin; *lacinia mobilis* little longer than incisor, with smooth edge; molar distally rounded, with blunt spine and eight, weak finger-shape setae (Fig. 14C”). Maxillule endite (Fig. 14D,D’) with nine strong distal spines and numerous setae along outer margin. Maxilla (Fig. 14D) semitriangular. Labium (Fig. 14E) with two lobes; inner lobe with numerous spines distally; outer lobe smaller than inner lobe, finely setose. Maxilliped (Fig. 14F) basis 1.8 L:W, with small distal seta; palp article-1 1.2 L:W, naked; article-2 as long as wide, with three serrate inner setae (two short, one long); article-3 1.7 L:W, with four sparsely hirsute inner setae; article-4 1.7 L:W, with five distal and subdistal, sparsely setose inner setae and one outer seta (Fig. 14F”); maxilliped endites separated, narrow, 2.0 L:W, with two setae and two slender gustatory cusps on distal margin, distolateral corner setose (Fig. 14F’). Epignath not seen.

Cheliped (Fig. 15A,B) sclerite large semitriangular; basis 1.8 L:W, with one subdistal dorsal seta; merus wedge-shape, with ventral seta; carpus 1.2 L:W, with two ventral setae, one subdistal and one subproximal dorsal setae; chela rather slender 1.2x carpus, 1.5 L:W; propodus (palm) with seta near dactylus insertion and four setae (one long, three short) and microtrichia on inner side; fixed finger as long as wide, with robust distal spine (unguis); incisive margin irregular and well calcified; with obtuse distal tooth; three setae on cutting edge and two ventral setae; fixed finger and dactylus unifacial; dactylus almost straight 3.3 L:W, with three short spines on cutting margin and two subproximal setae on inner side.

Pereopod-1 (Fig. 15C) longer than pereopods 2–3; basis 6.5 L:W, naked; ischium with seta; merus 1.8 L:W, 0.7x carpus, with one short distal spine; carpus 4.0 L:W, 0.8x propodus, with four distal spines; propodus 5.6 L:W, 2.9x dactylus, with one subdistal ventral short spine and one subdistal dorsal spine; dactylus 8.0 L:W, 2.0x unguis, with seta reaching 0.3 of unguis; unguis and dactylus about as long as propodus.

Pereopod-2 (Fig. 15D) coxa with seta; basis 5.4 L:W, naked; ischium with ventral seta; merus 2.5 L:W, 0.8x carpus, with serrate ventrodistal spine; carpus 3.1 L:W, 0.7x propodus, with one long and two short serrate spines and one minute spine distally; propodus 5.0 L:W and 2.6x dactylus, with subdistal ventral spine and subdistal fine and regular spines dorsodistally; dactylus 7.0 L:W, 0.6x unguis, with seta reaching 0.3 unguis, proximal seta not seen; dactylus and unguis as long as propodus.

Pereopod-3 (Fig. 15E) as pereopod-2.

Pereopod-4 (Fig. 15F) basis 3.2 L:W, with two penicillate ventral setae; ischium with two ventral setae; merus 3.0 L:W, 1.1x carpus, with two serrate ventrodistal spines; carpus 2.4 L:W, 0.7x propodus, with four distal serrate spines and rod-like seta distally; propodus 8.0 L:W, with middle penicillate seta on dorsal margin, two ventrodistal serrate spines and dorsodistal serrate spine; merus, carpus and propodus with microtrichia ventrally; dactylus 6.7 L:W; unguis broken.

Pereopod-5 (Fig. 15G) as pereopod-4, but no penicillate seta on basis and propodus.

Pereopod-6 (Fig. 15H) similar to pereopod-5, but propodus with three dorsodistal serrate spines.

Uropod (Fig. 15I) basal article 2.3 L:W; exopod with two articles, 0.7x endopod, just longer than endopod article-1, article-1 2.1 L:W, with distal penicillate seta, article-2 2.8 L:W, with two distal setae; endopod with two articles, article-1 2.1 L:W, naked, article-2 2.0 L:W, with five simple and one penicillate setae distally.

### Distribution

The species is known only from the type locality, NE Atlantic (Vema Fracture Zone), from 5127–5137 m depth. At this station the environmental parameters were as follow: bottom temperature: 2.21 °C, bottom O_2_: 245.4 μM, bottom current: 2.1 cm/s.

### Remarks

*Paranarthrurella kizomba* sp. nov. is one of eight species of *Paranarthrurella* present in the Atlantic, and the only one with long body habitus and no pleonite hyposphaenia. It is one of five species that have an elongate body (>8 L/W) and regular (wide) pleotelson with small apex directed downward (large apex directed backward in *P. caudata*). The new species is readily distinguished from *P. polonez* sp. nov. (see below) by presence of the smooth lateral margins of pleonites (pointed in *P. polonez*). The distal spine in propodus of pereopod 4–6 is weakly serrate in *P. kizomba*, in contrast to *P. dissimilis* (strongly serrate spine). Furthermore, *P. kizomba* can be distinguished from *P. spinimaxillipeda* (from Central Pacific) by having only one spine on the merus of the pereopods 1–3 (two in *P. spinimaxillipeda*). Finally, *P. kizomba* elongated seta in the maxilliped palp article-3, that is equal to the other setae in *P. samba* sp. nov. (see below).

*Paranarthrurella moonwalk* **Błażewicz and Jóźwiak sp. nov.**

Figures 16–19

**Figure 16:**
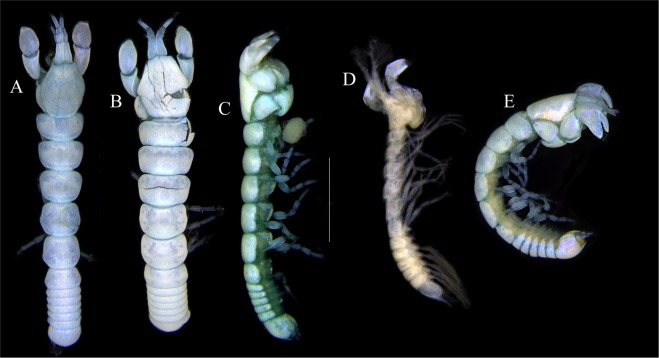
Species of the genus *Paranarthrurella* from Gay-Head Bermuda: (**A**) *P. rocknroll* sp. nov. (**B**–**E**) *P. moonwalk* sp. nov. (**A**) *P. rocknroll* sp. nov. holotype neuter (MCZ:IZ: 48498) dorsal view. (**B**) *P. moonwalk* sp. nov. paratype, male (MCZ:IZ: 48389). (**C**) Holotype, neuter (MCZ:IZ: 48323). (**D**) Paratype, male (MCZ: IZ: 49389), illustrated and dissected. (**E**) Paratype, juvenile male (MCZ:IZ:49378). Scale 1 mm.

### Material examined

Holotype neuter, with tantulocarid (3.4 mm BL) (MCZ:IZ:48323), RV *Atlantis II*, Cr. 30 St. 131.

Paratypes: neuter (4.1 mm BL, dissected), (MCZ:IZ:149577), the same locality as holotype; male (3.9 mm BL, partly dissected), (MCZ:IZ:49389), RV *Atlantis II*, Cr. 24, St. 115; two mancas-2 (1.8–1.9 mm BL) two mancas-3 (2.4–2.7 mm BL), (MCZ:IZ:49382), RV *Knorr* Cr. 35, St. 340; two mancas (2.0–2.1 mm BL), (MCZ:IZ:49384), RV *Chain*, Cr. 88, St. 210; neuter (3.3 mm BL) (MCZ:IZ:149576), RV *Atlantis II*, Cr. 24, St. 115.

### Additional material

*Paranarthrurella* cf. *moonwalk*, one juvenile male (4.1 mm BL), (MCZ:IZ:49378), RV *Knorr*, Cr. 35. St 340.

### Diagnosis

#### Female

Body short (<6.5 L:W). Pereonite-1 0.4 L:W. Pleonites lateral margin smooth. Pleonites 2 and 3 with hyposphaenium small and large, weakly pointed backward, respectively. Pleotelson rounded and swollen, apex small, pointed downward. Cheliped carpus 1.1 L:W. Pereopod-1 merus with two short and fine spines. Pereopods 4–6 carpus with four slender spines. Pereopods 4–6 propodus dorsodistal spine strongly serrate. Uropod endopod 1.5x exopod.

### Etymology

The species name is given for a dance initiated by Michael Jackson. Noun in apposition.

**Figure 17:**
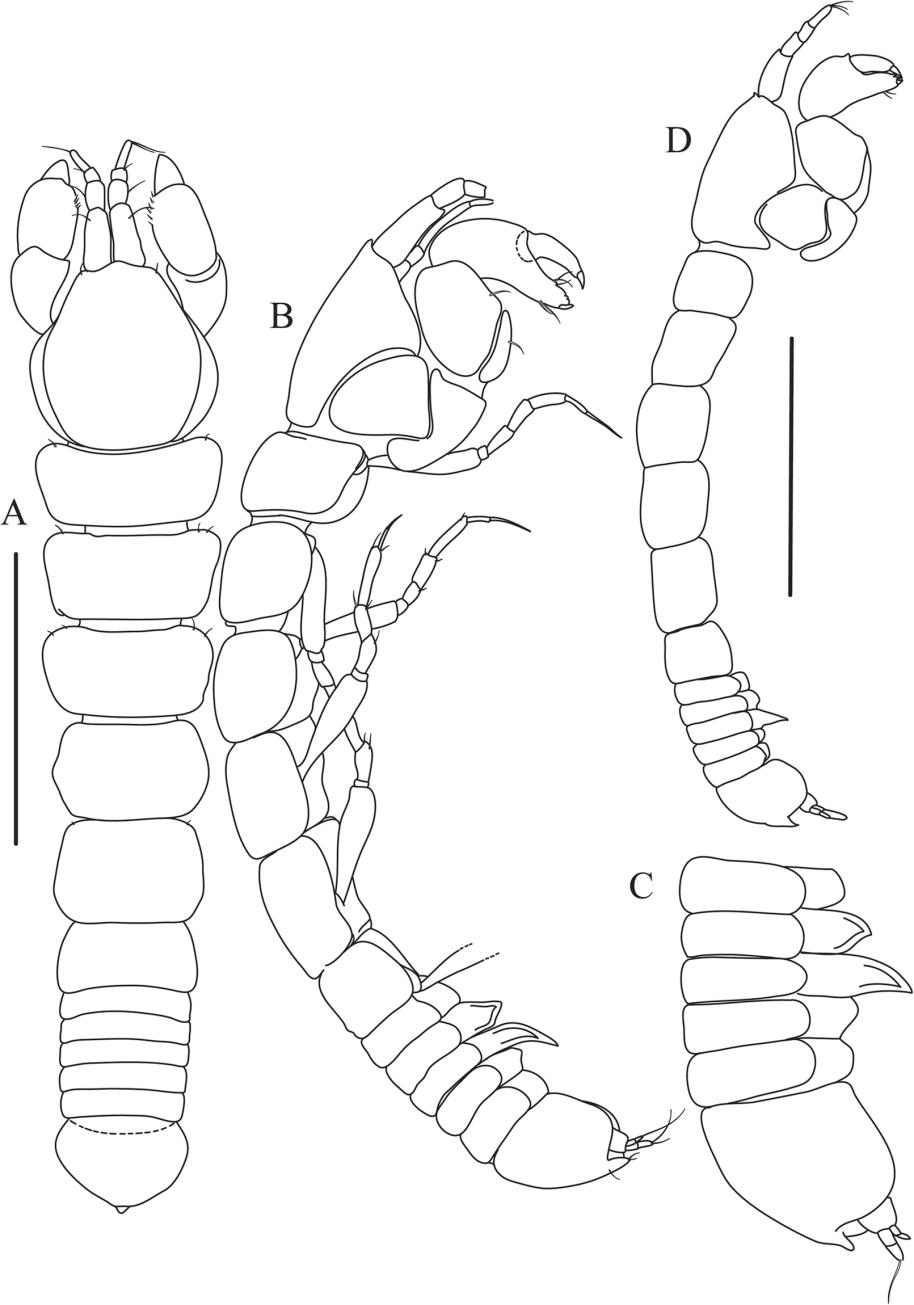
*Paranarthrurella moonwalk* sp. nov., holotype neuter (MCZ:IZ:48323), (**A**) Dorsal, (**B**) Lateral. (**C**) Female pleon lateral. (**D**) paratype, juvenile male, lateral (MCZ:IZ:49378). Scale 1 mm.

**Description of neuter**, length 4.2 mm. Body (Figs 16B–E and 17A,B) robust, 5.6 L:W; cephalothorax 1.2 L:W, 2.4x pereonite-1. Pereonites 1−6: 0.4, 0.5, 0.5, 0.6, 0.7 and 0.45 L:W, respectively; all pereonites wider than long; pereonites 1–2 wider proximally, pereonites 3–5 weakly rounded. Pleon 0.25 of total body length. All pleonites the same size, 0.25 L:W, pleonites 2–3 with hyposphaenium small and large, weakly pointed backward, respectively. Pleotelson 3.0x pleonite-5.

**Figure 18:**
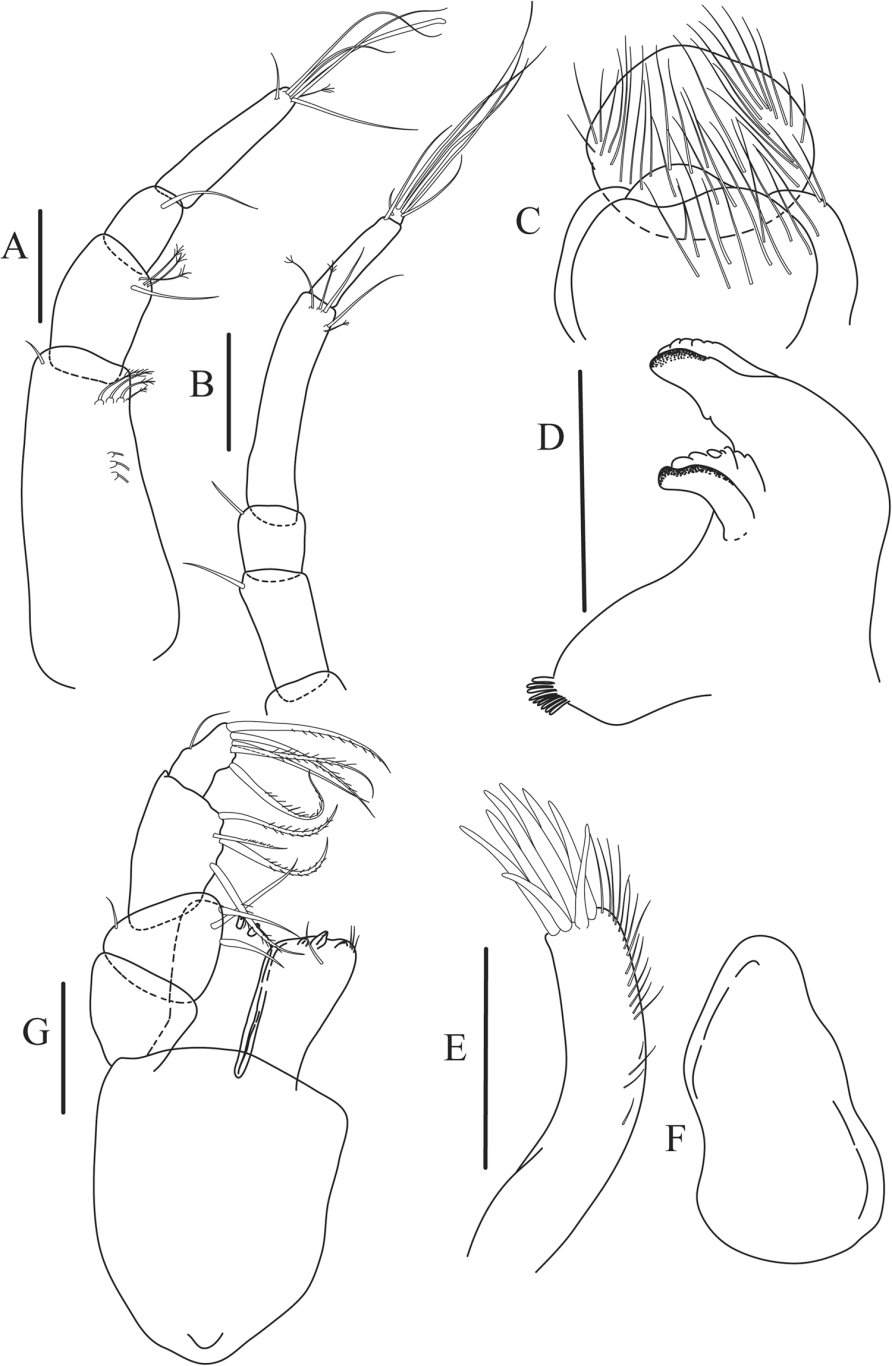
*Paranarthrurella moonwalk* sp. nov., paratype neuter (MCZ:IZ:149577) (**A**) Antennule. (**B**) Antenna. (**C**) Labrum. (**D**) Mandible, left. (**E**) Maxillula. (**F**) Maxilla. (**G**) Maxilliped. Scale 0.1 mm.

Antennule (Fig. 18A) article-1 2.7 L:W, 2.3x article-2, with three middle penicillate setae (broken), four penicillate distal setae and one simple distal seta; article-2 1.7 L:W, 1.7x article-3, with one simple and three penicillate distal setae; article-3 1.4 L:W, 0.5x article-4, with long distal seta; article-4 4.0 L:W; article-5, fully fused with article-4, with four long, one short and one penicillate distal setae, and aesthetasc.

Antenna (Fig. 18B) 0.85 times length of antennule; article-1 fused with body; article-2 2.1 L:W, 1.7x article-3, with distodorsal seta, situated in right angle to axis of the article; article-3 1.2 L:W, 0.3x article-4, with distodorsal seta, situated in right angle to axis of the article; article-4 5.5 L:W, 2.1x article-5, with two simple and three penicillate setae distally; article-5 4.2 L:W, with distal seta; article-6 1.2 L:W, with four long and two short distal setae.

Mouthparts. Labrum (Fig. 18C) large, distally rounded and with relatively dense and long setae. Right mandible not observed. Left mandible (Fig. 18D) incisor narrow with undulated margin; *lacinia mobilis* well developed, shorter than incisor, with undulate margin; molar distally rounded, with nine, weak finger-shape setae. Maxillule endite (Fig. 18E) with nine strong distal spines and numerous setae along outer margin; palp not seen. Maxilla (Fig. 18F) semi-triangular. Labium not observed. Maxilliped (Fig. 18G) basis 1.6 L:W, naked; palp article-1 1.1 L:W, naked; article-2 0.9 L:W, with three inner setae and outer seta; article-3 1.6 L:W, with four inner, sparsely serrate setae; article-4 1.8 L:W, with five distal and subdistal, sparsely setose inner setae and one outer seta; maxilliped endites separated, narrow, 2.2 L:W, with two slender gustatory cusps and seta on distal margin. Epignath not seen.

**Figure 19:**
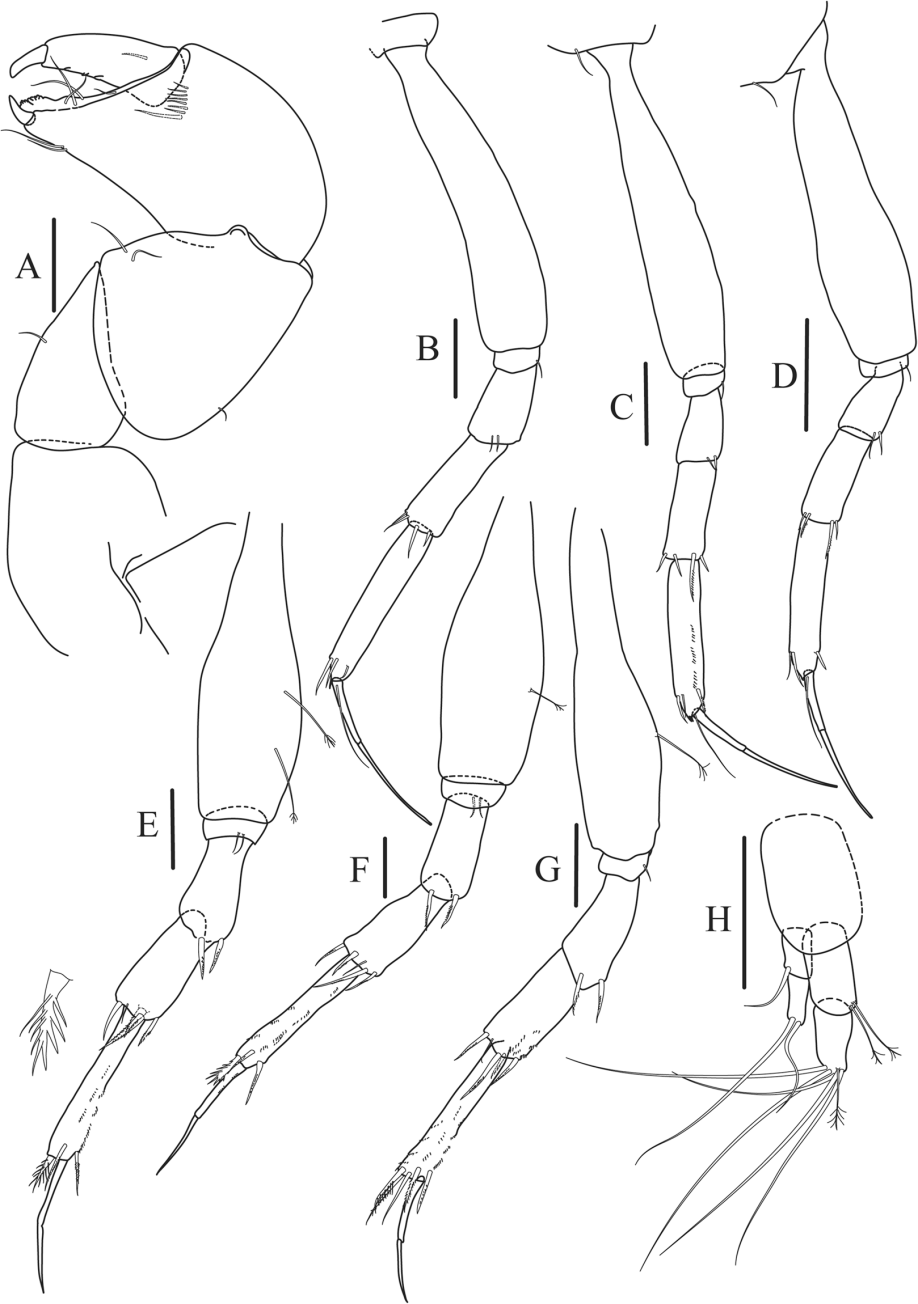
*Paranarthrurella moonwalk* sp. nov., paratype neuter (MCZ:IZ:149577) (**A**) Cheliped. (**B**–**G**) Pereopods 1–6, respectively. (H) Uropod. Scale 0.1 mm.

Cheliped (Fig. 19A) sclerite large, semitriangular; basis 1.9 L:W, subdistal seta not seen; merus wedge-shape, with ventral seta; carpus 1.2 L:W, with two ventral setae, one subdistal dorsal seta, subproximal seta not seen; chela slender, 1.3x carpus, 1.5 L:W; propodus (palm) with one seta near dactylus insertion and eight setae on inner side (one long, seven short); fixed finger as long as wide, with robust distal spine (unguis); incisive margin distally well calcified, with irregular margin, divided by two transversal ridges; three setae on cutting margin and two ventral setae; fixed finger and dactylus unifacial; dactylus almost straight, 3.3 L:W, with three short spines on cutting margin and subproximal seta on inner side.

Pereopod-1 (Fig. 19B) longer than pereopods 2–3; basis 5.3 L:W, naked; ischium with seta; merus 1.6 L:W, 0.8x carpus, with two short and fine distal spines; carpus 2.7 L:W, 0.7x propodus, with one short and three longer distal spines; propodus 5.2 L:W, 2.7x dactylus, with one subdistal ventral seta and two subdistal dorsal spines; dactylus 9.0 L:W, 2.7x unguis, with seta reaching 0.2 of unguis; unguis and dactylus about as long as propodus.

Pereopod-2 (Fig. 19C) coxa with seta; basis 5.4 L:W, naked; ischium with ventral seta; merus 1.8 L:W, 0.8x carpus, with two small (fine and regular) distal spines; carpus 2.6 L:W, 0.6x propodus, with one serrate spine and three spines distally; propodus 4.8 L:W and 2.4x dactylus, with subdistal serrate spine ventrally, two subdistal spines (regular and fined) dorsally; dactylus 9.0 L:W, 0.6x unguis, with seta reaching 0.2 unguis; dactylus and unguis as long as propodus.

Pereopod-3 (Fig. 19D) as pereopod-2.

Pereopod-4 (Fig. 19E) basis 2.9 L:W, with two penicillate ventral setae; ischium with two ventral setae; merus 2.3 L:W, as long as carpus, with two serrate spines; carpus 2.4 L:W, 0.8x propodus, with four distal spines (one longer and serrate), and seta dorsodistally; propodus 8.0 L:W, with two ventrodistal long serrate spines and dorsodistal strongly serrate spine; dactylus 8.0 L:W; unguis 0.9x dactylus; together as long as propodus.

Pereopod-5 (Fig. 19F) as pereopod-4, but basis with one penicillate seta.

Pereopod-6 (Fig. 19G) similar to pereopod-5, but propodus with three dorsodistal serrate spines.

Uropod (Fig. 19H) basal article 1.4 L:W; exopod with two articles, 0.7x endopod, article-1 1.8 L:W, with distal seta, article-2 2.7 L:W, with two distal setae; endopod with two articles, article-1 2.1 L:W, with two penicillate distal setae, article-2 1.6 L:W, with five simple and one penicillate setae distally.

### Distribution

The species is known only from type locality: Gay Head-Bermuda transect (NW Atlantic) from the depth of 2178 m.

### Remarks

*Paranarthrurella moonwalk* sp. nov. is one of two species with a short body habitus and large hyposphaenium on pleonite-3 and a small hyposphaenium on pleonite-2. Another species with likewise distributed hyposphaenia is the Australian *P. corroboree* (see remarks above). Two other species present in the North Atlantic are *P. voeringi* and *P. arctophylax* (see remarks in relevant sections).

***Paranarthrurella polonez*** **Błażewicz and Jóźwiak, sp. nov.**

Figures 20–23

**Figure 20:**
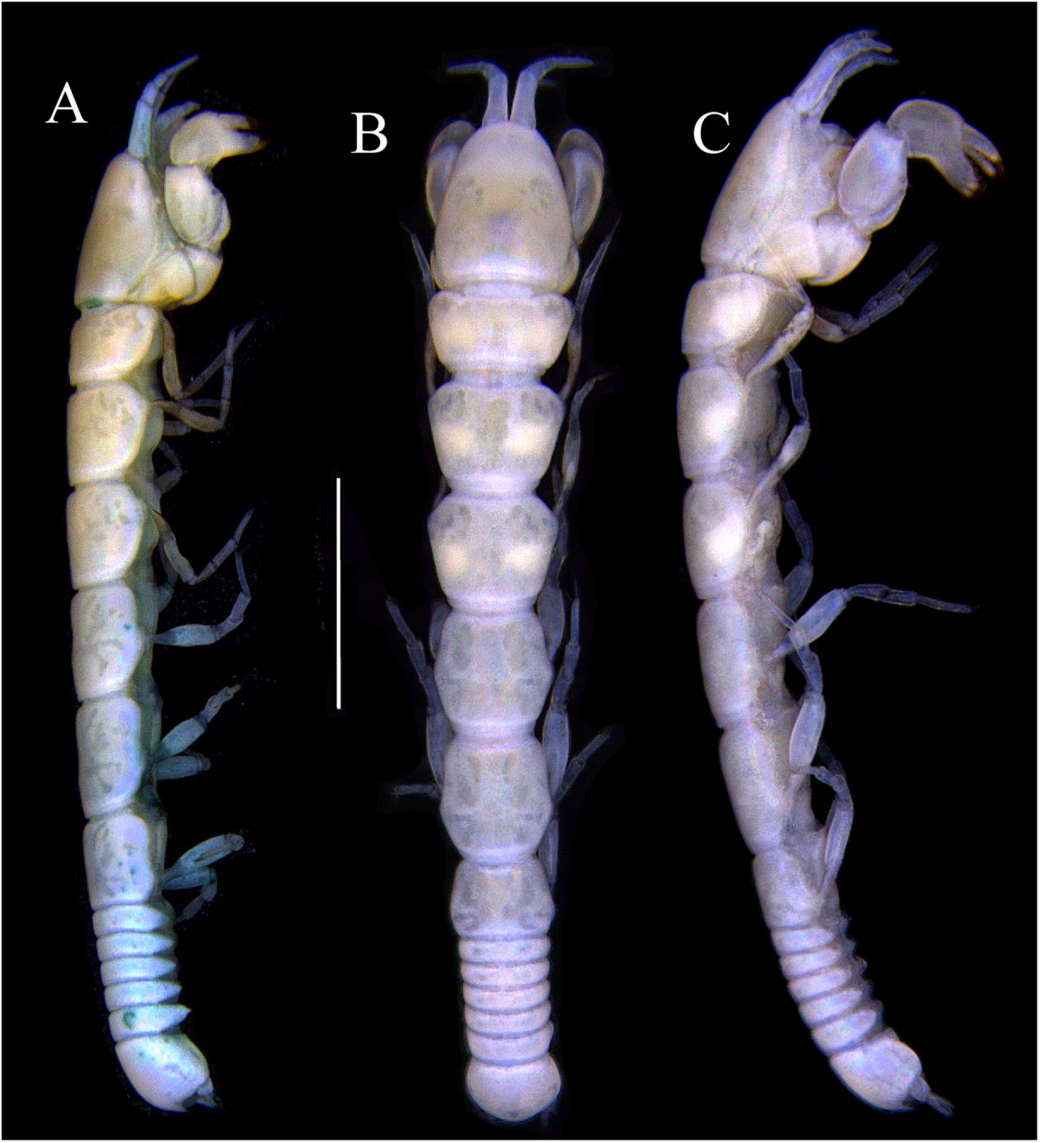
*Paranarthrurella polonez* sp. nov. (**A**) neuter, dorsal (ind. 169, lost). (**B,C**) Holotype neuter, dorsal and lateral (ZMH K-55988). Scale 1 mm.

**Figure 21:**
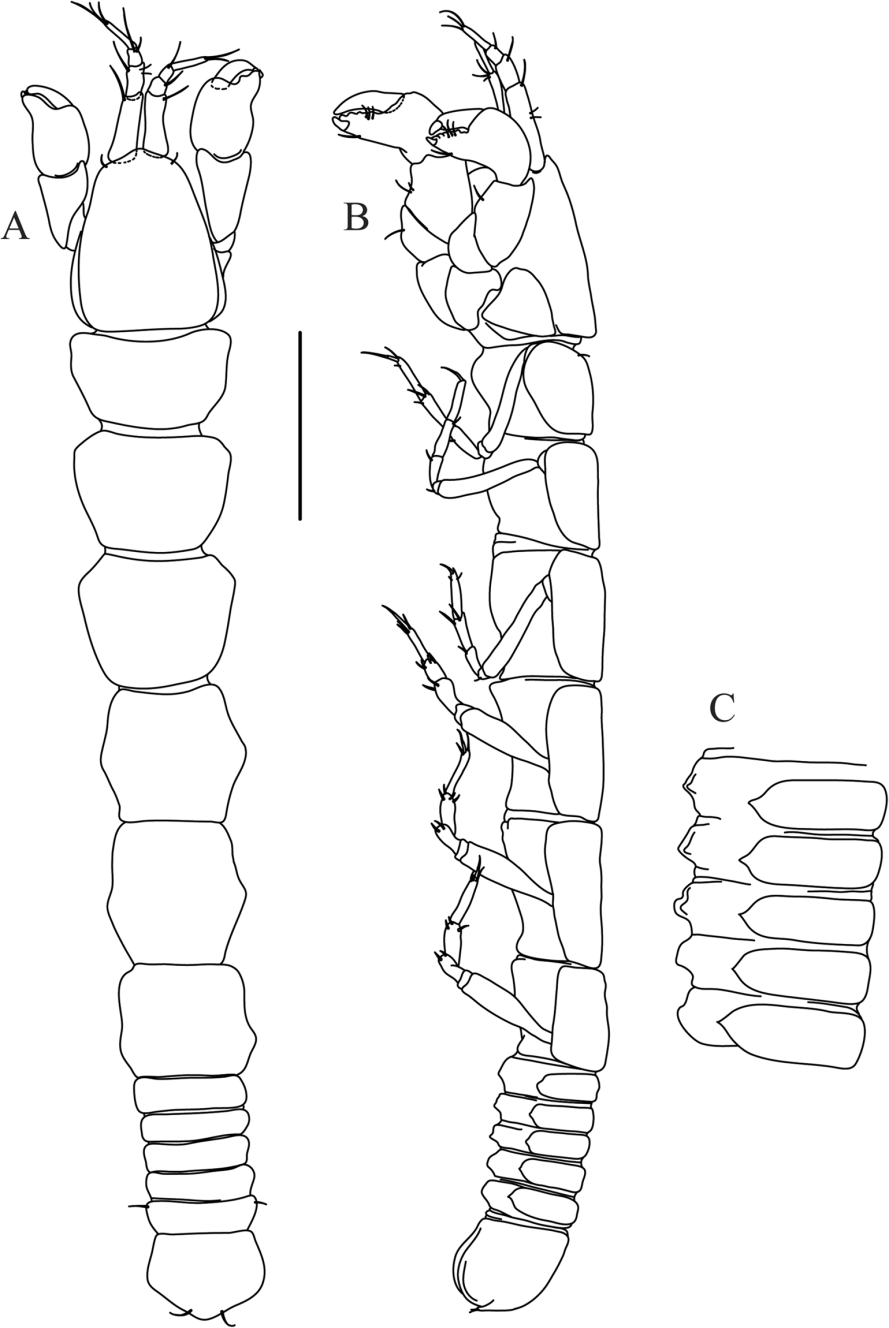
*Paranarthrurella polonez* sp. nov. Neuter (ind. 169, lost) (**A**) Dorsal. (**B**) Lateral. (**C**) pleon lateral. Scale 1 mm.

**Figure 22:**
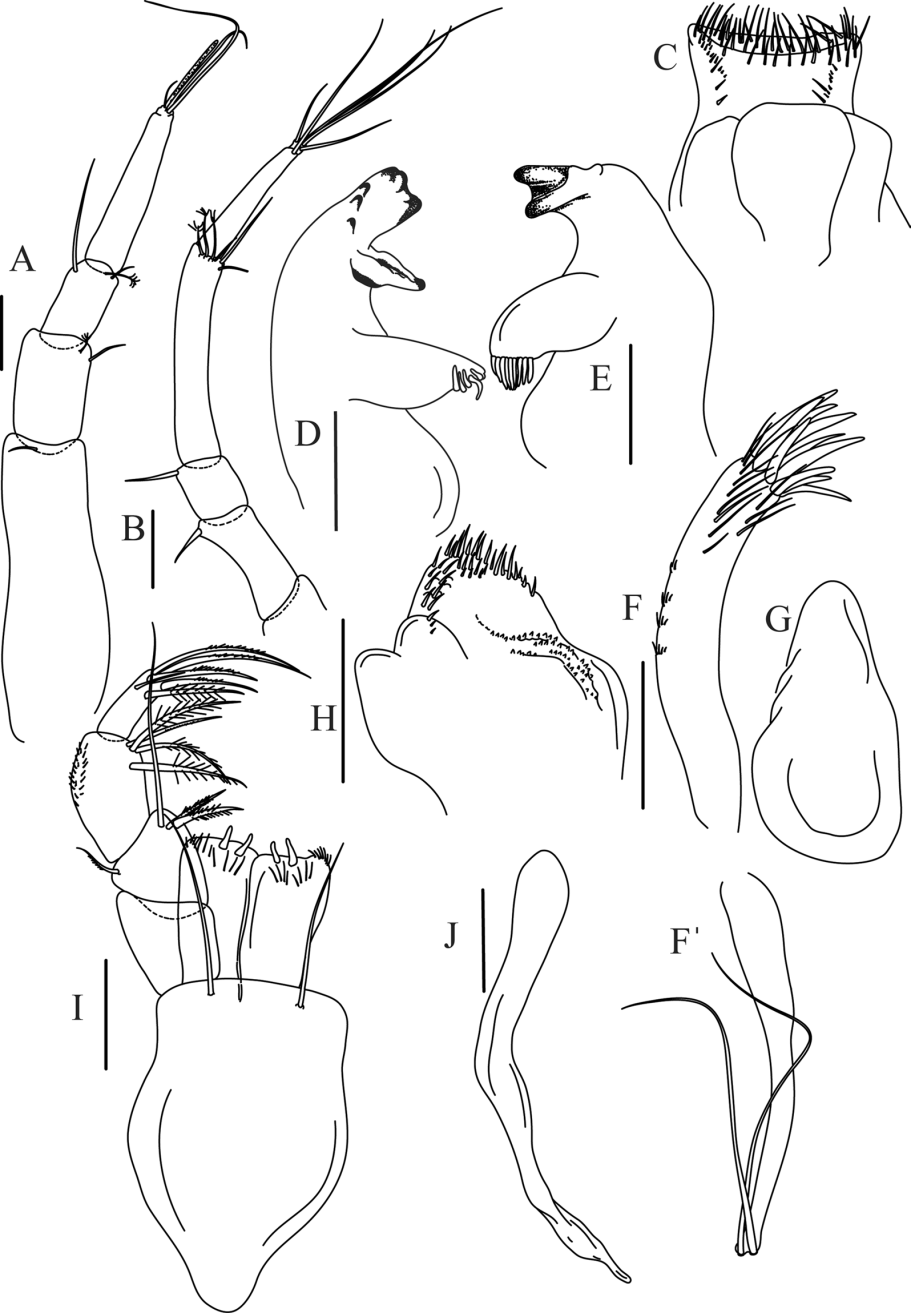
*Paranarthrurella polonez* sp. nov. paratype, neuter (ZMH K-55993) (**A**) Antennule. (**B**) Antenna. (**C**) labrum. (**D**) Mandible, left. (**E**) Mandible right. (**F**) Maxillule, endite; (**F’**) Maxillule, palp. (**G**) Maxilla. (**H**) Labium. **(I**) Maxilliped. (**J)** Epignath. Scale 0.1 mm.

**Figure 23:**
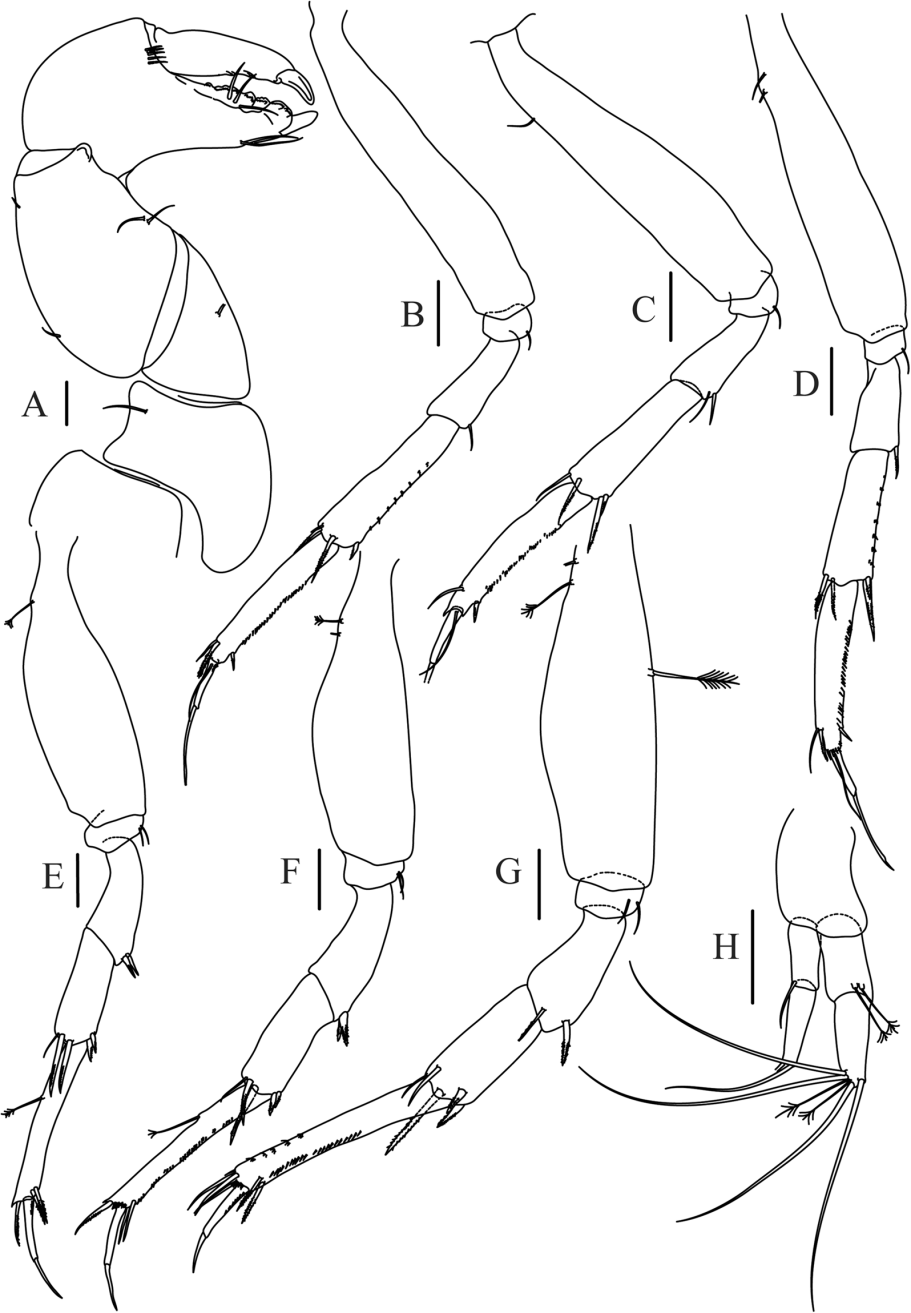
*Paranarthrurella polonez* sp. nov., paratype, neuter (ZMH K-55993) (**A**) Cheliped. (**B**–**G**) Pereopod 1–6, respectively. (**H**) Uropod. Scale 0.1 mm.

### Material examined

Holotype: neuter (4.6 mm BL) (ZMH K-55988), (ind. 279), RV *Sonne* (SO-239), St. 95 (IOM).

Paratypes: manca-2 (3.5 mm BL) (ZMH K-55989), (ind. 280), the same locality as holotype; neuter (length 4.3 mm BL) (ZMH K-55992), (ind. 171), manca-3 (3.6 mm BL) (ZMH K-55990), (ind. 173), one neuter (3.0 mm BL) (ZMH K-55991), (ind. 170), neuter (ind 169, lost), RV *Sonne* (SO-239), St. 81 (IOM); female (4.8 mm BL) (ZMH K-55994), (ind. 298), juvenile male(?) (2.9 mm BL) (ZMH K-55995), (ind. 299), RV *Sonne* (SO-239), St. 99 (IOM); neuter (4.8 mm BL, dissected) (ZMH K-55993). (ind. 266), RV *Sonne* (SO-239), St. 106 (IOM).

### Diagnosis

#### Female

Body long (>8 L:W). Pereonite-1 0.8 L:W. Pleonites lateral margin pointed. Pleonites 2–4 without hyposphaenia. Pleon rounded and swollen, apex small. Cheliped carpus 1.6 L:W. Pereopod-1 merus with fine spine. Pereopods 4–6 carpus with four spines. Pereopods 4–6 propodus dorsodistal spine finely serrate. Uropod endopod 1.1x exopod.

### Etymology

The species name is given for a dance of Polish origin. Noun in apposition.

**Description of neuter**, length 4.3 mm. Body (Fig. 21A,B) slender, 7.7 L:W; cephalothorax 1.3 L:W, 2.3x pereonite-1. Pereonites 1−6: 0.8, 1.2, 1.1, 1.1, 1.2 and 0.6 L:W, respectively; pereonites 2–5 subsquare, pereonites 1–3 wider proximally, pereonites 4–5 wider in midlength. Pleon (Fig. 21C) 0.2 of total body length. All pleonites the same size, 0.3 L:W. Pleotelson 2.5x pleonite-5, rounded; apex small directed downward.

Antennule (Fig. 22A) article-1 3.0 L:W, 2.5x article-2, with small distal seta; article-2 1.6 L:W, 1.8x article-3, with simple and penicillate distal setae; article-3 1.95 L:W, 0.6x article-4, with long, short and penicillate setae distally; article-4 4.75 L:W; article-5 minute, partly fused with article-4, with at least two long and one short distal setae and aesthetasc.

Antenna (Fig. 22B) 0.7 times length of antennule; article-1 fused with body; article-2 2.5 L:W, 1.5x article-3, with distodorsal seta, set at right angle to axis of the article; article-3 1.25 L:W, 0.3x article-4, with distodorsal seta, situated in right angle to axis of the article; article-4 5.8 L:W, 1.5x article-5, with three simple and three penicillate distal setae; article-5 4.1 L:W, with distal seta; article-6 as long as wide, with four long and two short distal setae.

Mouthparts. Labrum (Fig. 22C) large, elongate, distally obtuse and with dense and robust setae. Right mandible (Fig. 22E) incisor upper edge simple; *lacinia mobilis* fused with incisor, prominent, edge simple; molar lobeform, distally weakly rounded, with nine weak, finger-shape setae. Left mandible (Fig. 22D) incisor with three rounded projections subdistally and two distally separated by short gap; *lacinia mobilis* well developed, narrow little longer than incisor; molar with five weak, finger-shape setae. Maxillule endite (Fig. 22F)with ten strong distal spines of various length and numerous setae along inner and outer margin; palp (Fig. 22F’) with two long simple setae. Maxilla (Fig. 22G) semi-triangular, simple. Labium (Fig. 22H) with two lobes; inner lobe with numerous spines on inner margin and with strong setae distally; outer lobe bilobed smaller than inner lobe, with simple margin. Maxilliped (Fig. 22I) basis 1.3 L:W, with long seta overeaching endites; palp article-1 1.1 L:W, naked; article-2 1.2 L:W, with three inner setae (one very long) and outer seta; article-3 1.5 L:W, with four inner setae (three sparsely serrate); article-4 2.7 L:W, with distal and subdistal inner setae (one sparsely setose, four serrate), and outer seta; maxilliped endites separated, narrow, 2.2 L:W, distally with two slender gustatory cusps. Epignath (Fig. 2J) distally narrowed and pointed.

Cheliped (Fig. 23A) sclerite large semitriangular; basis 1.3 L:W, with subdistal dorsal seta; merus wedge-shape, with ventral seta (broken); carpus 1.7 L:W, with two ventral setae, one subdistal and one subproximal dorsal setae; chela slender, 1.2x carpus, 1.7 L:W; propodus (palm) with one seta near dactylus insertion and five setae on inner side; fixed finger with robust distal spine; incisive margin distally well calcified, edge irregular, with two transversal grooves and with small tooth in midlength; three setae on cutting margin and two ventral setae; fixed finger and dactylus unifacial; dactylus almost straight with three short spines on cutting margin, subproximal seta on inner side not seen.

Pereopod-1 (Fig. 23B) longer than pereopods 2–3; basis 5.5 L:W, naked; ischium with seta; merus 2.5 L:W, 0.55x carpus, with ventrodistal fine spine; carpus 4.4 L:W, subequal to propodus, with four distal spines (one short and three long); propodus 6.1 L:W, 3.0x dactylus, with one short ventrodistal spine, two dorsodistal spines (fine and longer serrate); dactylus 0.7x unguis, with seta reaching 0.2 of unguis; unguis and dactylus about 0.7x propodus.

Pereopod-2 (Fig. 23C) basis 5.0 L:W, with subproximal dorsal seta; ischium with ventral seta; merus 2.9 L:W, 0.6x carpus, with two ventrodistal fine and regular spines; carpus 3.75 L:W, 0.9x propodus, with four distal serrate spines; propodus 5.25 L:W and 3.0x dactylus, with subdistal ventral short spine and dorsodistal fine and serrate spine (seta broken), ventral margin with microtrichia; dactylus 7.0 L:W, with seta reaching over proximal part of unguis; unguis broken.

Pereopod-3 (Fig. 23D) similar to pereopod-2, but basis with two subproximal penicillate setae (one broken); unguis almost twice as long as dactylus, together 0.8x propodus.

Pereopod-4 (Fig. 23E) basis 3.1 L:W, with subproximal distal penicillate seta; ischium with two ventral setae; merus 2.6 L:W, as long as carpus, with two ventrodistal serrate spines; carpus 2.9 L:W, 0.6x propodus, with four distal spines and one rod-like seta; propodus 7.3 L:W, with penicillate middorsal seta, two ventrodistal serrate spines and one serrate dorsodistal spine; dactylus 9.6 L:W; unguis 0.7x dactylus; together 0.7x propodus.

Pereopod-5 (Fig. 23F) similar to pereopod-4, but basis with two penicillate setae.

Pereopod-6 (Fig. 23G) similar to pereopod-5, but basis with two subproximal dorsal and one midventral penicillate setae; propodus without dorsodistal penicillate seta and with three dorsodistal serrate spines.

Uropod (Fig. 23H) basal article 1.6 L:W; exopod with two articles, 0.9x endopod, article-1 2.5 L:W, with distal seta, article-2 3.5 L:W, with two distal setae; endopod with two articles, article-1 1.95 L:W, with two penicillate distal setae; article-2 2.65 L:W, with four simple and two penicillate setae distally.

### Distribution

The species occurs in Clarion Clipperton Zone, in Interoceanmetal claim area (IOM), at the depth 4365–4823 m.

### Remarks

*Paranarthrurella polonez* sp. nov. can be distinguished from all other congeners by the pointed lateral margins of the pleonites. It is one of two *Paranarthrurella* species occurring in the Central Pacific in the area associated with manganese nodules. *Paranarthrurella spinimaxillipeda* has smooth margins of the pleonites, but also the exopod uropod clearly shorter than the endopod, while the uropod exopod is only a little shorter than the endopod in *P. polonez*.

***Paranarthrurella rocknroll*** **Błażewicz and Jóźwiak sp. nov.**

**Figure 24:**
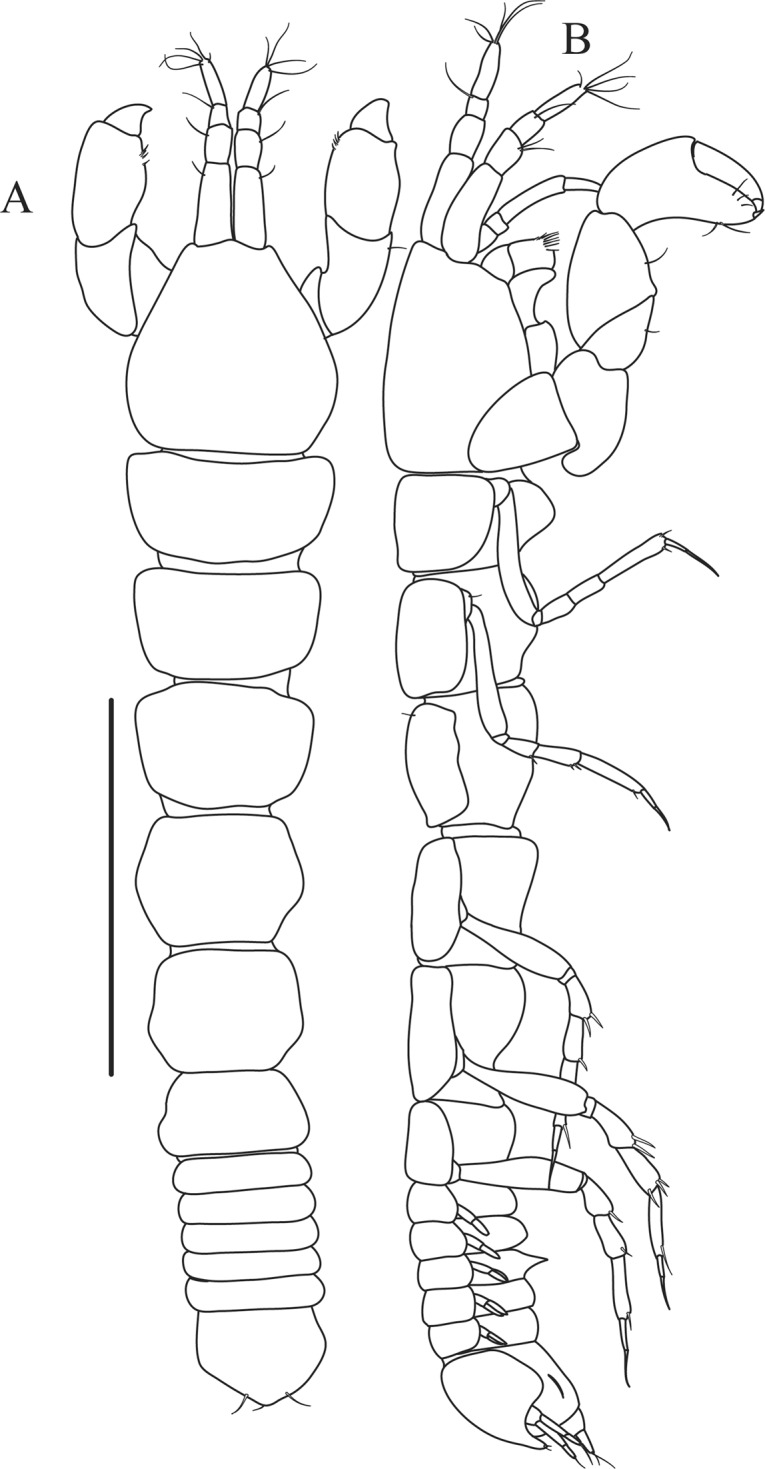
*Paranarthrurella rocknroll* sp. nov. holotype, neuter (MCZ:IZ:48498) (**A**) dorsal; (**B**) lateral. Scale 1 mm.

**Figure 25:**
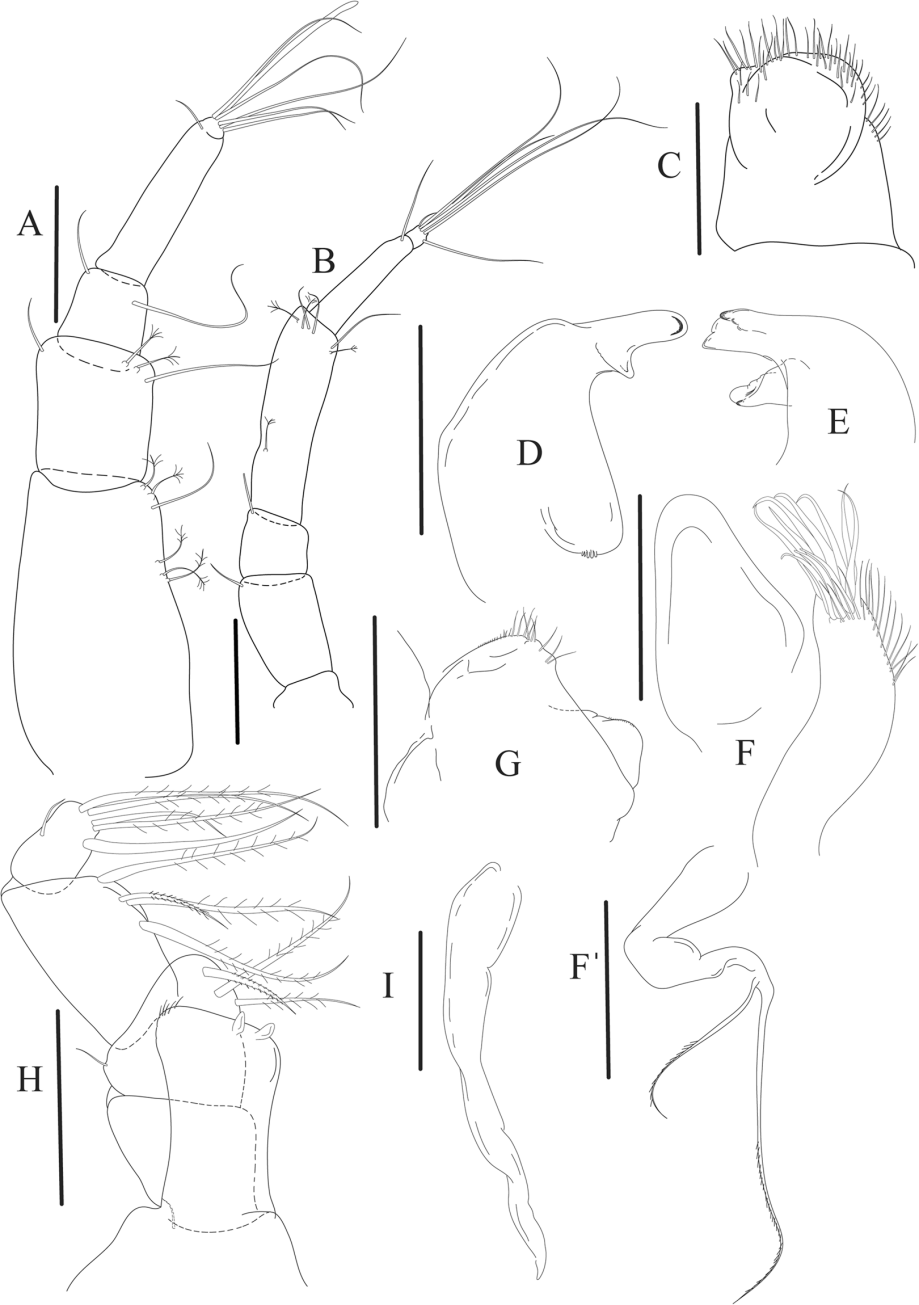
*Paranarthrurella rocknroll* sp. nov., holotype, neuter (MCZ:IZ:48498) **(A**) Antennule. (**B**) Antenna. (**C**) Labrum. (**D**) Mandible, right. **(E**) Mandible, left. **(F)** Maxillula and maxilla. (**F’**) Maxillula, palp. (**H**) Maxilliped. (**I**) Epignath. Scale 0.1 mm.

**Figure 26:**
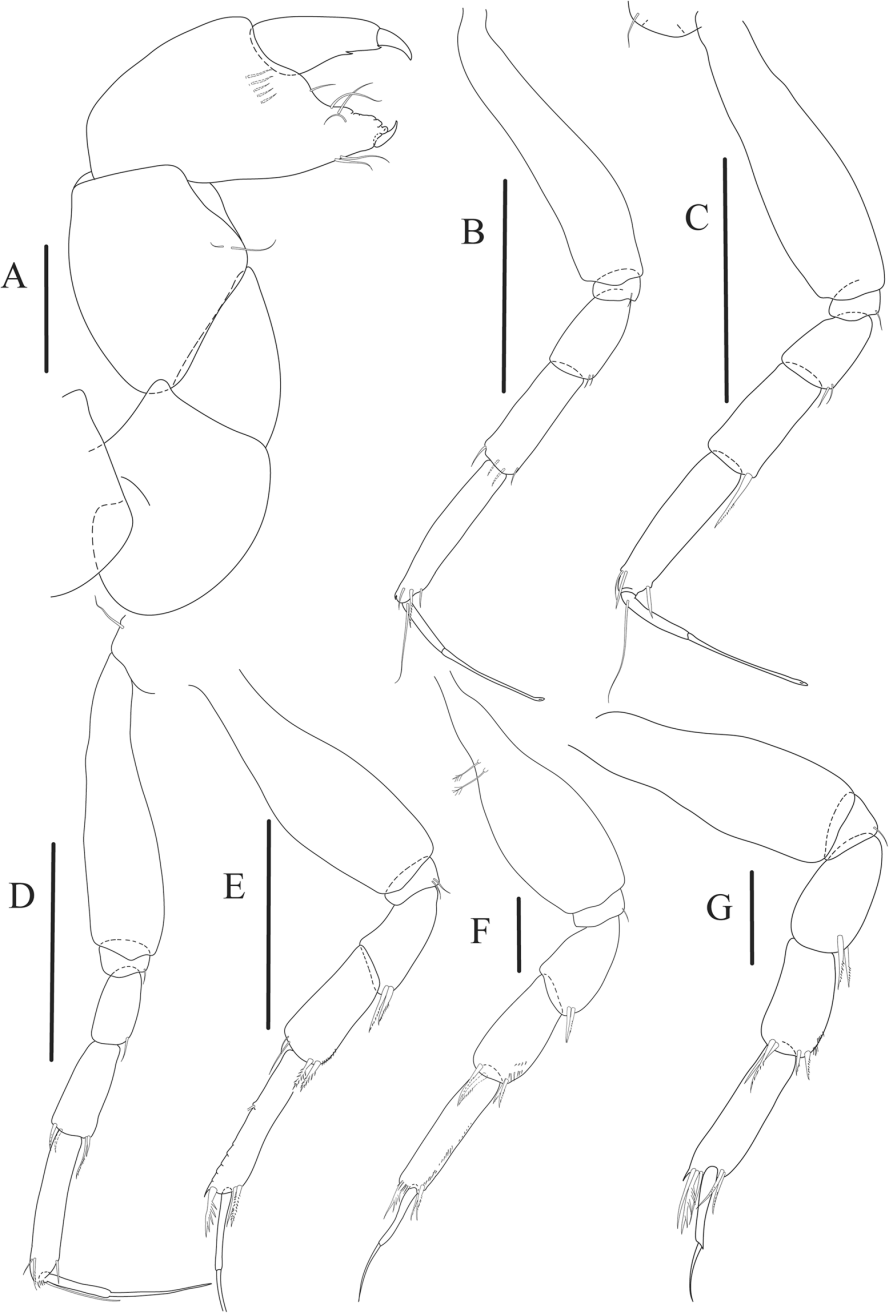
*Paranarthrurella rocknroll* sp. nov., holotype, neuter (MCZ:IZ:48498) (**A)** Cheliped. (**B**–**G**) Pereopod 1–6, respectively. Scale 0.1 mm.

Figures 16 and 24–26

### Material examined

Holotype, neuter, (3.27 mm BL, partly dissected), (MCZ:IZ:48498), RV *Atlantis II*, Cr. 24, St. 120.

### Diagnosis

#### Female

Body short (<6.5 L:W). Pereonite-1 0.5 L:W. Pleonites lateral margin smooth. Pleonite 2 and 4 without hyposphaenium; pleonite-3 with small hyposphaenium. Pleon rounded and swollen, apex small. Cheliped carpus 1.3 L:W. Pereopod-1 merus with two small setae. Pereopods 4–6 carpus with four spines. Pereopods 4–6 propodus dorsodistal spine strongly serrate. Uropod endopod 1.5x exopod.

### Etymology

The species name is given for a genre of dance originated and evolved in the United States of America. Noun in apposition.

**Description of neuter**, length 3.27 mm. Body (Figs 16 and 24A,B) robust, 6.0 L:W; cephalothorax 1.1 L:W, 2.2x pereonite-1. All pereonites wider than long. Pereonites 1−6: 0.5, 0.6, 0.7, 0.7, 0.7 and 0.6 L:W, respectively. All pereonites wider than long; pereonites 1–3 wider proximally, pereonites 4–5 wider in midlength. Pleon 0.25 of total body length. All pleonites the same size, 1.8 W:L. Pleonite-3 with small pointed triangular hyposphaenium. Pleotelson 4.0x pleonite-5.

Antennule (Fig. 25A) article-1 1.9 L:W, 2.1x article-2, with three penicillate setae at midlength, one simple and two penicillate setae distally; article-2 1.3 L:W, 1.6x article-3, with two simple and two penicillate setae distally; article-3 1.3 L:W, 0.6x article-4, with two long setae distally; article-4 4.0 L:W; article-5 almost completely fused with article-4, with five long and one short setae, and with aesthetasc distally.

Antenna (Fig. 25B) 0.8 times length of antennule; article-1 fused with body; article-2 1.5 L:W, 1.5x article-3, with distodorsal seta; article-3 1.1 L:W, 0.3x article-4, with distodorsal seta; article-4 3.6 L:W, 1.7x article-5, with penicillate seta at midlength, three simple and three penicillate setae distally; article-5 4.2 L:W, with distal seta; article-6 minute, with four long and two short distal setae.

Mouthparts. Labrum (Fig. 25C) hood-shape, distally setose. Left mandible (Fig. 25E) incisor distally cut, undulate, *lacinia mobilis* margins undulate; molar process not seen. Right mandible (Fig. 25D) incisor margin simple, distally rounded, *lacinia mobilis* in shape of small protrusion; molar process broad and rounded distally, with row of minute finger-shape spines. Maxillule endite (Fig. 25F) with ten, relatively long and slender spines, distal and dorsodistal margin setulose; palp (Fig. 25F’) with two distal serrate setae. Maxilla (Fig. 25F) triangular, naked. Labium (Fig. 25G) bilobed; inner lobe with numerous, short setae along distal margin; outer lobe, vestigial, simple. Maxilliped (Fig. 25H) basis partly broken during dissection, with minute distal seta; palp article-1 as long as wide, naked; article-2 as long as wide, with three inner setae and one outer seta; article-3 1.4 L:W, with four inner setae; article-4 1.7 L:W, with five serrate setae distally and one simple subdistal seta; maxilliped endites separate, with a pair of slender gustatory cusps, distouter corner setulate. Epignath (Fig. 25I) distally pointed, naked.

Cheliped (Fig. 26A) sclerite large triangular; basis 1.6 L:W; merus ventral margin longer than that of carpus, ventral seta not seen; carpus wider medially, 1.4 L:W, with two ventral setae, dorsal setae not seen; chela 1.3x carpus, propodus (palm) with one longer and four short setae on inner side and seta near dactylus insertion; fixed finger with two simple setae ventrally and three setae on cutting edge, incisive margin well calcified, with uneven blunt teeth; fixed finger and dactylus unifacial; dactylus weakly bent downward, with weak spines on cutting margin.

Pereopod-1 (Fig. 26B) basis 5.0 L:W, naked; ischium with ventral seta; merus 2.0 L:W, 0.8x carpus, with two weak ventrodistal spines; carpus 2.9 L:W, 0.8x propodus, with four fine spines distally; propodus 4.7 L:W, 2.5x dactylus, with small ventral spine and two unequal fine dorsal spines distally; dactylus 0.6x unguis, with proximal seta; unguis and dactylus about 1.1x propodus.

Pereopod-2 (Fig. 26C) coxa with seta; basis 3.6 L:W, naked; ischium with ventral seta; merus 1.2 L:W, with two ventrodistal spines (slender and robust); carpus 2.3 L:W, 0.7x propodus, with long and short ventrodistal spine; propodus 3.8 L:W and 2.3x dactylus, with fine and strong subdistal dorsal spines and ventrodistal spine; dactylus 0.6x unguis, with one proximal seta; dactylus and unguis 1.2x propodus.

Pereopod-3 (Fig. 26D) as pereopod-2, but only one spine on merus, and four distal spines on carpus observed (three short one longer).

Pereopod-4 (Fig. 26E) basis3.0 L:W; ischium with two ventral setae; merus 2.0 L:W, 0.9x carpus, with two ventrodistal serrate spines; carpus 2.3 L:W, 0.7x propodus, with two simple, fine dorsodistal spines and two ventrodistal serrate spines; propodus 7.0 L:W, with one penicillate dorsal seta (broken), two ventrodistal serrate spines and strongly serrate dorsodistal spine; unguis broken.

Pereopod-5 (Fig. 26F) similar to pereopod-4, but basis with two penicillate dorsal setae, dactylus 1.1x unguis; dactylus and unguis 0.9x propodus.

Pereopod-6 (Fig. 26G) similar to pereopod-5, but basis naked, propodus with two ventrodistal spines and three dorsodistal spines (one strongly serrate).

Uropod (not illustrated) basal article about 1.5 L:W; exopod with two articles, about 0.5x endopod, article-1 about 2.2 L:W, article-2 about 2.5 L:W, with at least two distal setae; endopod with two articles, article-1 about 2.0 L:W; article-2 about 2.5 L:W, with four simple distal setae.

### Distribution

Species known only from the type locality, Gay Head-Bermuda transect, between 5018 and 5023 m depth.

### Remarks

*Paranarthrurella rocknroll* sp. nov. is the second species with a short body and hyposphaenium present only on pleonite-3. The hyposphaenium is small and pointed downward distinguishing it from *P. arctophylax* occurring in the North Atlantic; it has hyposphaenium large and weakly pointed backward^80^.

***Paranarthrurella samba*** **Błażewicz and Jóźwiak sp. nov.**

Figures 27–32

**Figure 27:**
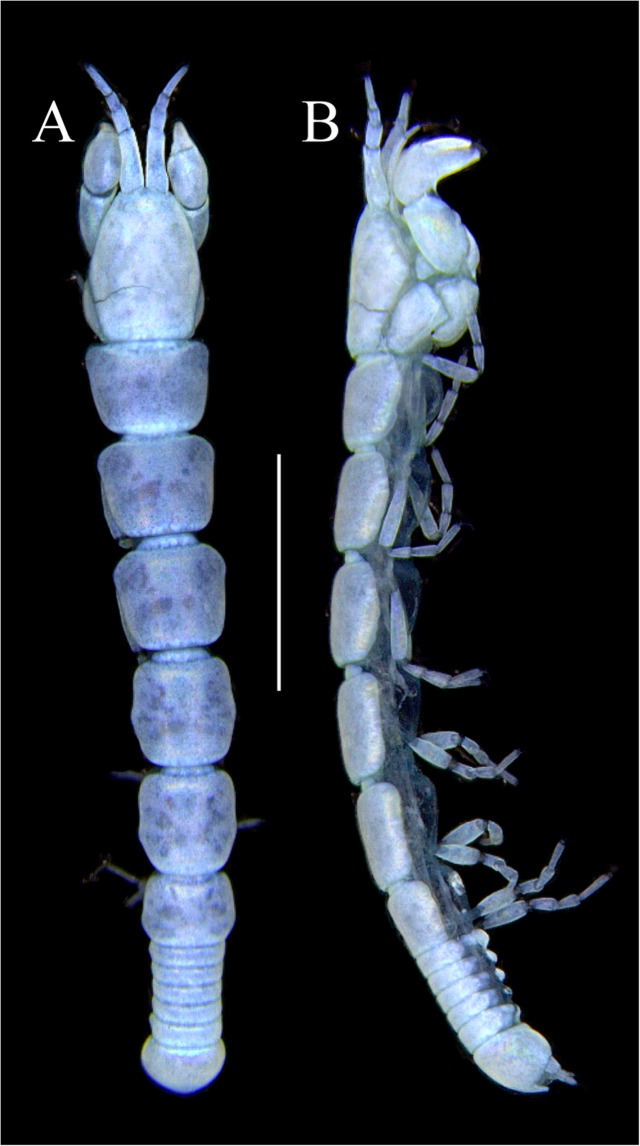
*Paranarthrurella samba* sp. nov. **(A,B**) holotype, neuter (MCZ:IZ:49400). Scale 1 mm.

**Figure 28:**
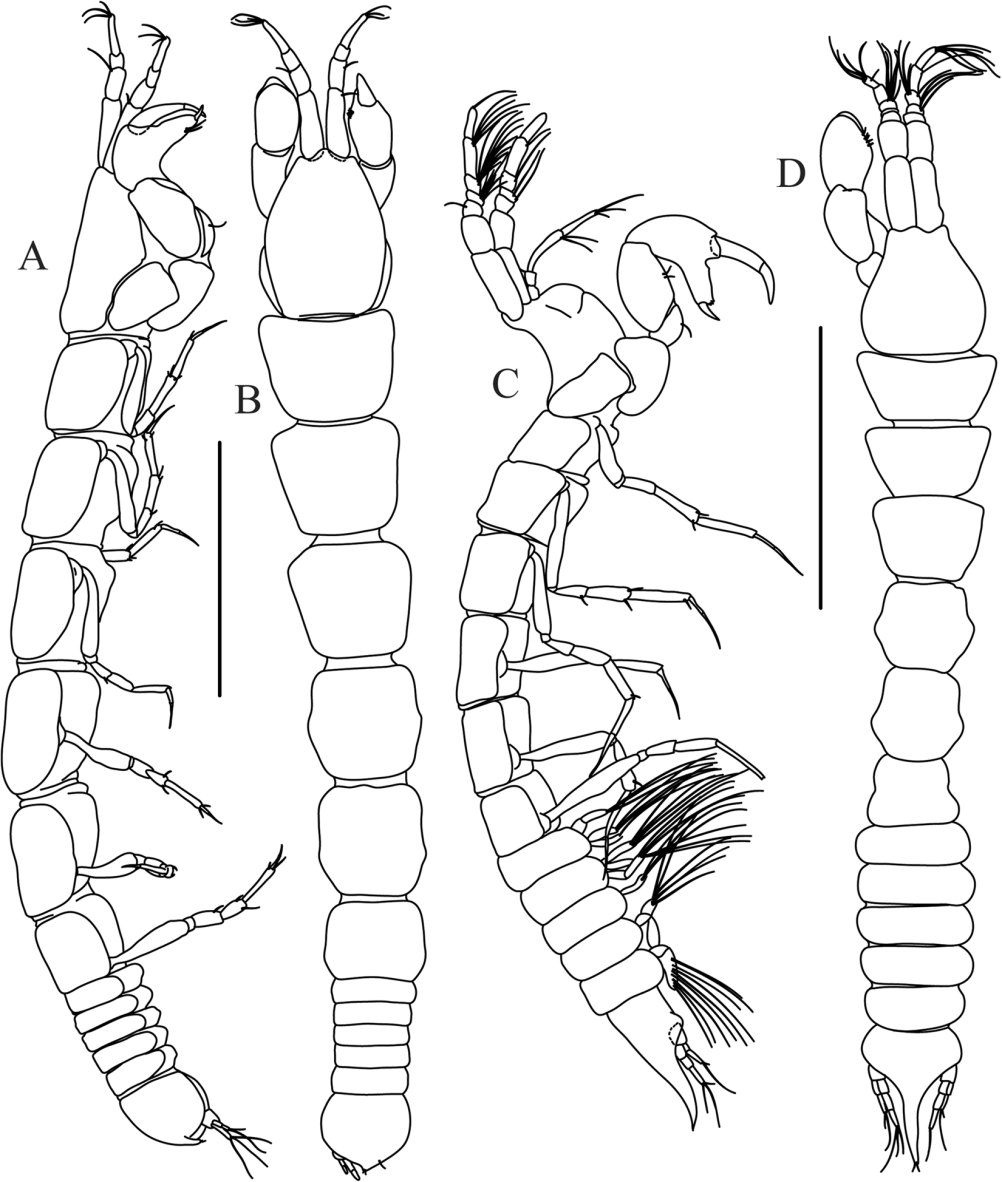
*Paranarthrurella samba* sp. nov. holotype, neuter (MCZ:IZ:49400) (**A**) lateral and (**B**) dorsal. Male (MCZ:IZ:149578) (**C**) lateral and (**D**) dorsal, respectively. Scale 1 mm.

**Figure 29:**
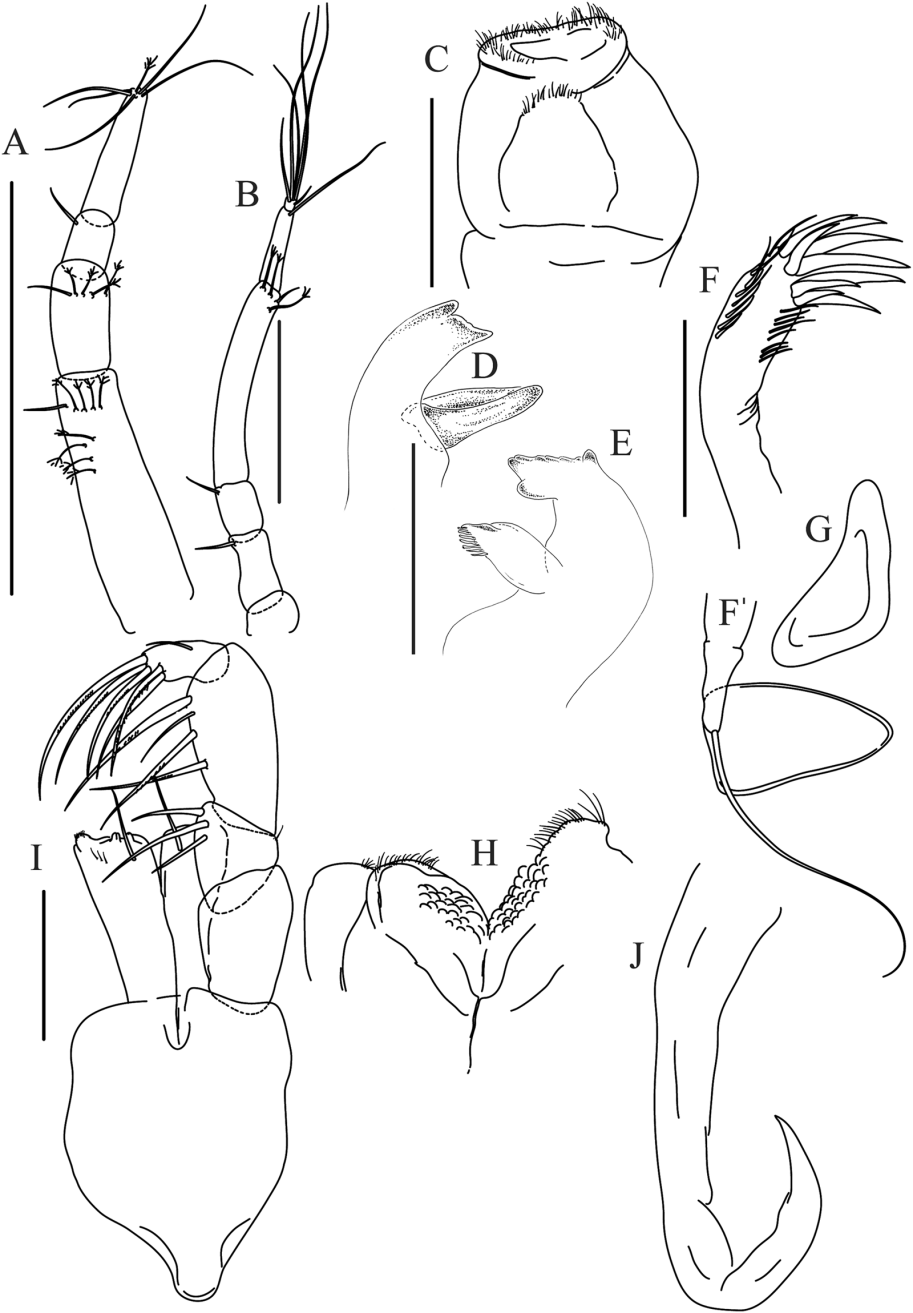
*Paranarthrurella samba* sp. nov., paratype, neuter (MCZ:IZ:149579) (**A**) Antennule. (**B**) Antenna. (**C**) Labrum. (**D)** Mandible, left. (**E**) Mandible, right. (**F**) Maxillule. (**F’**) Maxillule endite. (**G**) Maxilla. **(H**) Labium. (**I**) Maxilliped. (**J**) Epignath. Scale 0.1 mm.

**Figure 30:**
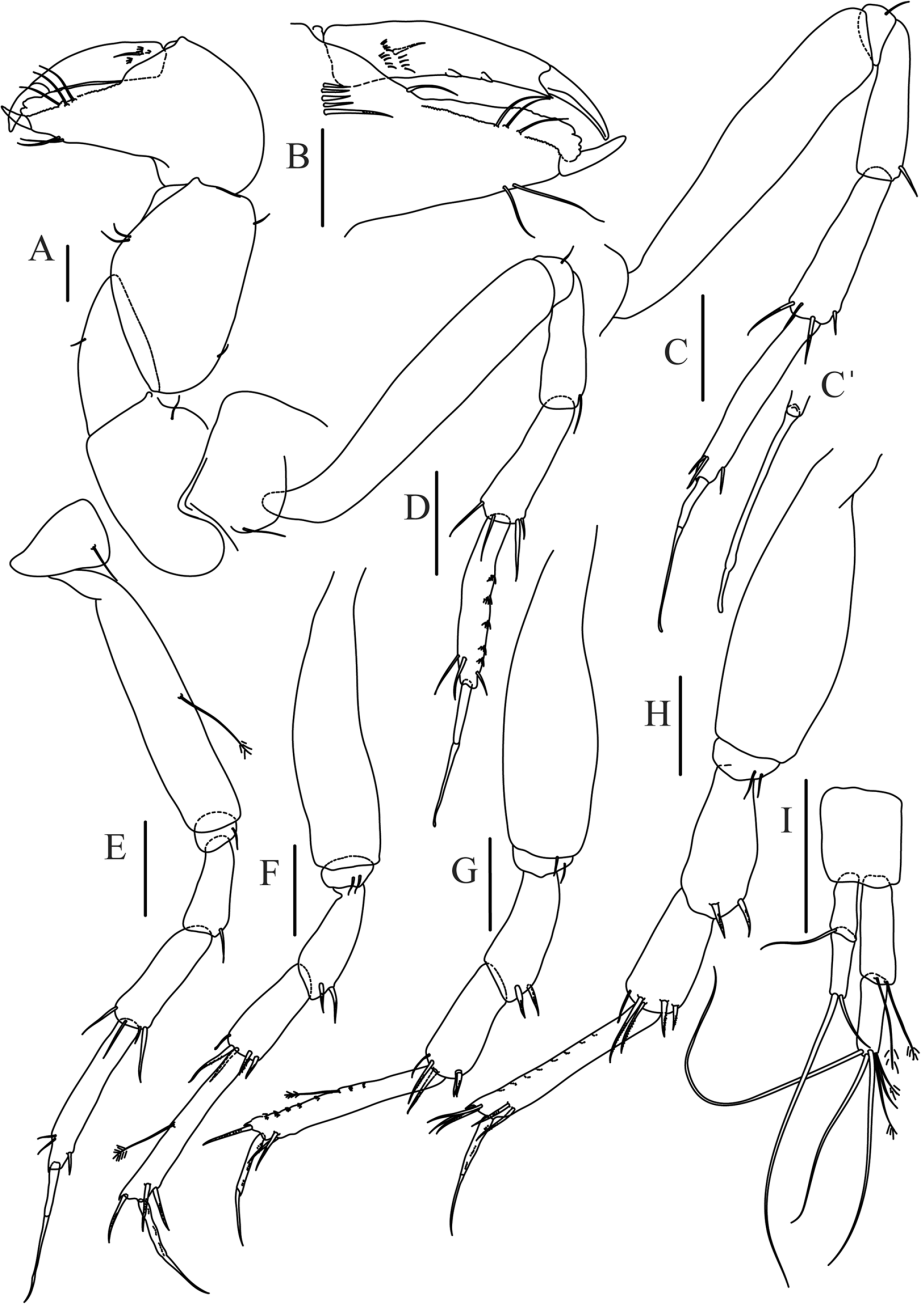
*Paranarthrurella samba* sp. nov., paratype, neuter (MCZ:IZ:149579) (**A**) Cheliped. (**B**) Chela inner side. (**C**–**H**) Pereopod 1–6, respectively. (**I)** Uropod. Scale 0.1 mm.

**Figure 31:**
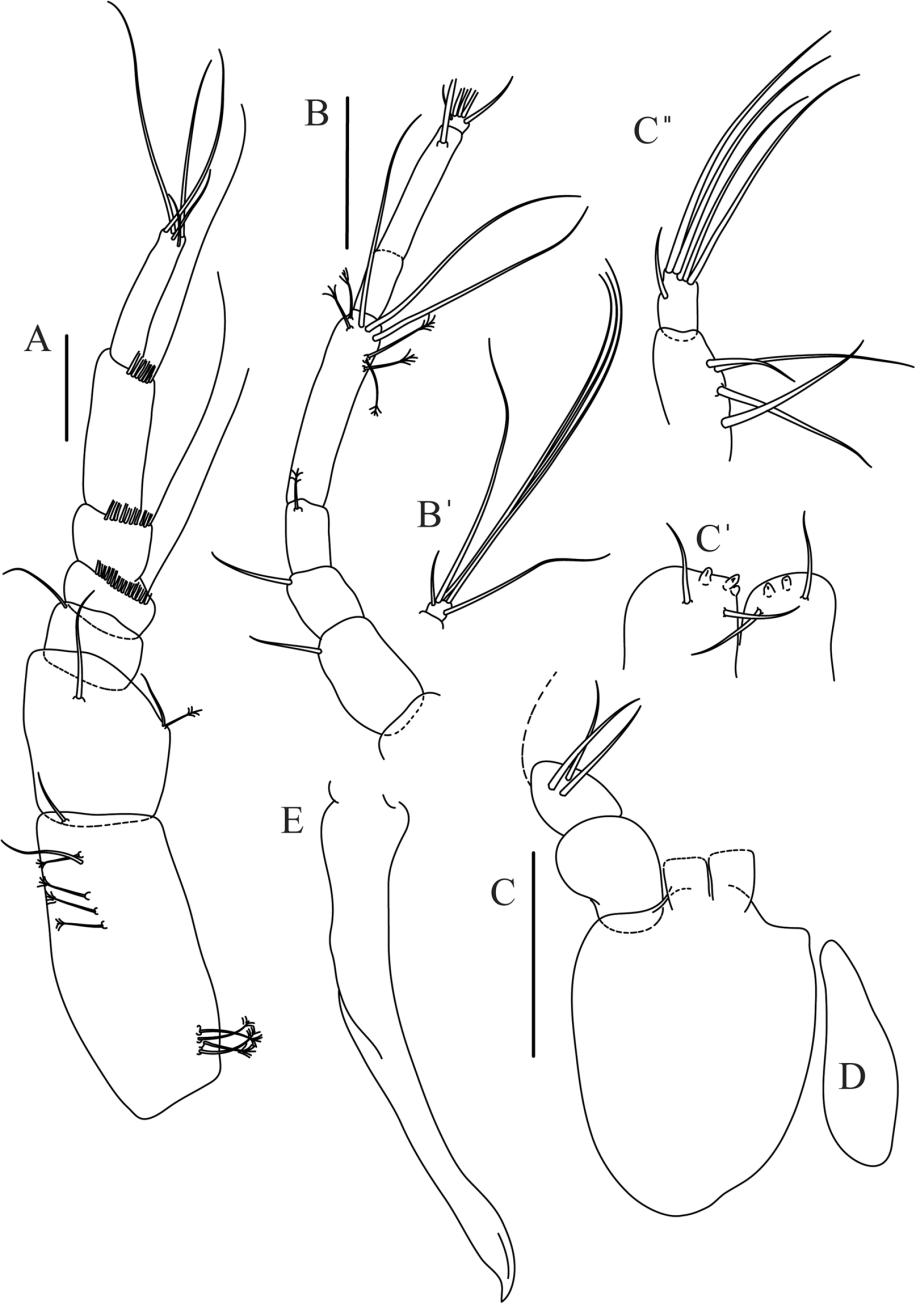
*Paranarthrurella samba* sp. nov., paratype male (MCZ:IZ:149578) (**A**) Antennule. (**B**) Antenna. (**C**) Maxilliped. (**C’**) Maxilliped, endite distally. (**C”**) Maxilliped palp distal part. (**D**) Maxilla. (**E**) Epignath. Scale 0.1 mm.

**Figure 32:**
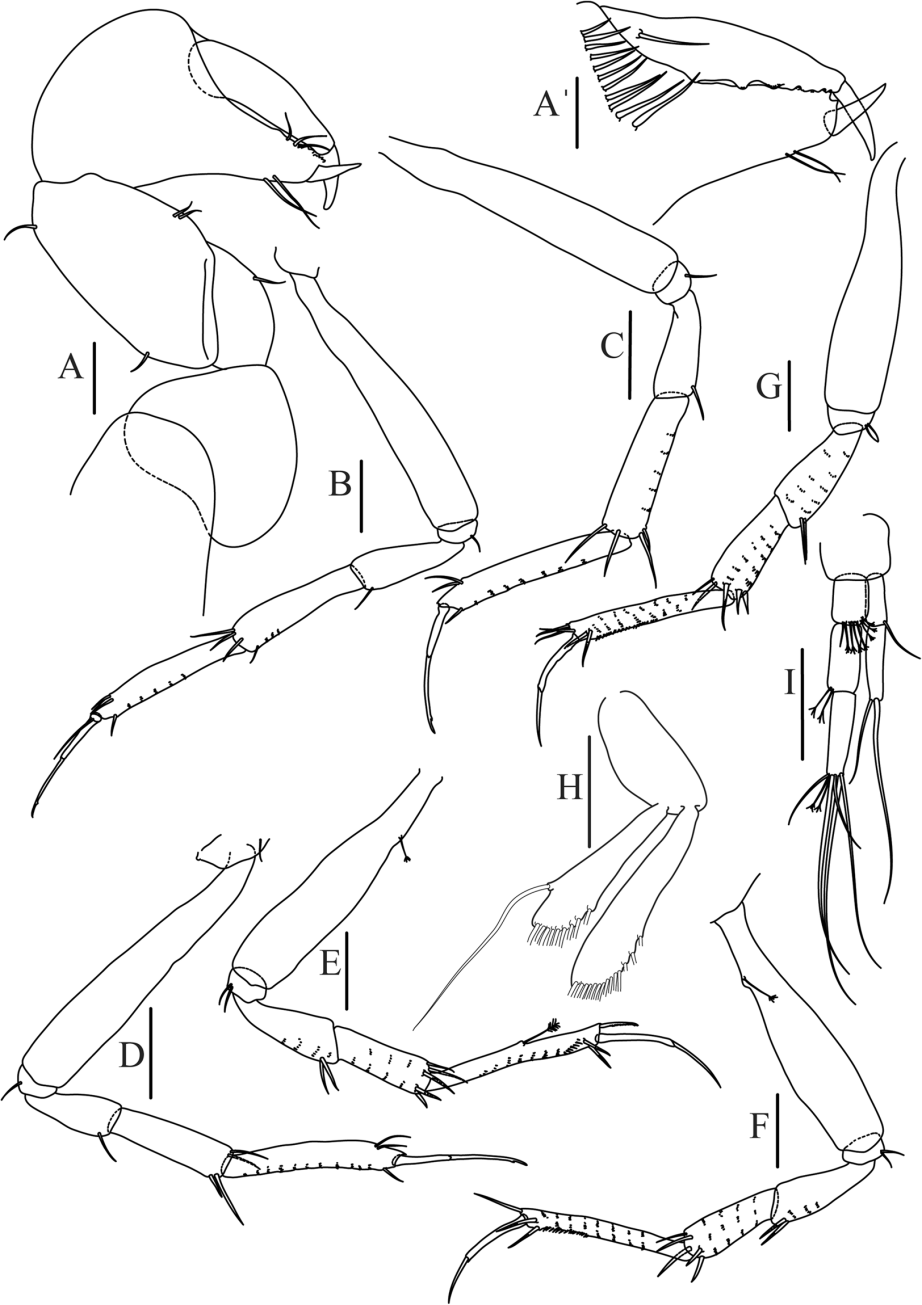
*Paranarthrurella samba* sp. nov., paratype male (MCZ:IZ:149578) (**A**) Cheliped. (**A’**) Chela, inner side. (**B**–**G**) Pereopod 1–6, respectively. (**H**) Pleopod. (**I**) Uropod. Scale 0.1 mm.

### Material examined

Holotype: neuter (4.5 mm BL), (MCZ:IZ:49400), RV *Atlantis II*, Cr. 31, St. 156.

#### Paratypes

Male, partly dissected, 3.4 mm BL (MCZ:IZ:149578); neuter, dissected (MCZ:IZ:149579); three juvenile males, 2.7–3.3 mm BL, neuter, 3.6 mm BL, two mancas-2, 2.0–2.6 mm BL, five mancas-2, 1.4–2.5 mm BL (MCZ:IZ:149580); two females, 3.5–4.1 mm BL, male, 5.0 mm BL, two mancas 2.5–2.5 mm BL (MCZ:IZ:149581), the same locality as holotype.

### Diagnosis

#### Female

Body long (>8.0 L:W). Pereonite-1 0.8 as long as wide. Pleonites lateral margin smooth. Pleonites 2–4 without hyposphaenium. Pleon round, apex small pointed downward. Cheliped carpus 1.6 L:W. Pereopod-1 merus with one fine spine. Pereopods 4–6 carpus with four spines. Pereopods 4–6 propodus dorsodistal spine finely serrate. Uropod endopod 1.4x exopod.

### Etymology

The species that was found off Brazilian coast is called after a samba – Brazilian musical genre and dance style. Noun in apposition.

**Description of neuter**, length 4.5 mm. Body (Fig. 28A,B) slender, nine L:W; cephalothorax 1.4 L:W, 1.7x pereonite-1. Pereonites 1−6: 0.8, 0.9, 1.0, 1.0, 1.1 and 0.75 L:W, respectively; pereonites 2–5 subsquare, pereonites 1–3 wider proximally, pereonite slightly wider in midlenght. Pleon 0.2 of total body length. All pleonites the same size, 0.3 L:W. Pleotelson 3.0x pleonite-5.

Antennule (Fig. 29A) article-1 3.6 L:W, 2.2x article-2, with eight penicillate and one simple setae subdistally; article-2 1.9 L:W, 1.5x article-3, with one simple and four penicillate setae subdistally; article-3 1.7 L:W, 0.5x article-4, with distal seta; article-4 4.3 L:W; article-5 vestigial semifused with article-4, with five simple and one penicillate setae distally; aesthetasc not observed.

Antenna (Fig. 29B) 0.7 times length of antennule; article-1 fused with body; article-2 2.3 L:W, 1.3x article-3, with distodorsal seta, situated in right angle to axis of the article; article-3 1.7 L:W, 0.3x article-4, with distodorsal seta; article-4 7.3 L:W, 2.4x article-5, with one simple and three penicillate setae distally; article-5 1.9 L:W, with long distal seta; article-6 as long as wide, with four long and two short distal setae.

Mouthparts. Labrum (Fig. 29C) large, distally obtuse and densely setose. Right mandible (Fig. 29E) incisor upper edge weakly undulate, with blunt proximal process, *lacinia mobilis* relatively small and rounded; molar lobeform, distally acuminate, with six to seven weak, finger-shape setae. Left mandible (Fig. 29D) incisor narrower than in right mandible, distally obtuse and weakly undulate, with distal and proximal spines; *lacinia mobilis* well developed, little longer than incisor, edge simple. Maxillule endite (Fig. 29F) with nine strong distal spines of various length and numerous setae along inner and outer margin; palp (Fig. 29F’) with two long simple setae. Maxilla (Fig. 29G) semitriangular. Labium (Fig. 29H) with two lobes; inner lobe with numerous crenulations at inner margin and densely setose distally; outer lobe little smaller than inner lobe, margin simple. Maxilliped (Fig. 29I) basis 1.3 L:W, naked; palp article-1 1.6 L:W, naked; article-2 1.2 L:W, with three inner and one outer setae; article-3 2.1 L:W, with four inner setae (three long, one short); article-4 2.25 L:W, with five distal and subdistal inner setae; one outer seta; maxilliped endites separated, narrow, 3.0 L:W, distally with one seta and two slender gustatory cusps. Epignath (Fig. 29J) distally narrowed and pointed.

Cheliped (Fig. 30A,B) sclerite large semitriangular; basis 1.25 L:W, with one subdistal dorsal seta; merus wedge-shape, with ventral seta; carpus little wider medially, 1.7 L:W, with two ventral setae, one subdistal and one subproximal dorsal setae; chela slender, 1.2x carpus, 2.25 L:W; propodus (palm) with one seta near dactylus insertion and five setae on inner side (one long, four short); fixed finger with robust distal spine (unguis), with three inner setae and two ventral setae, cutting margin weakly, irregularly undulate; fixed finger and dactylus unifacial; dactylus 6.5 L:W, almost straight, with two spines on cutting margin and subproximal seta on inner side.

Pereopod-1 (Fig. 30C,C’) longer than pereopods 2–3; coxa with seta; basis 4.75 L:W; ischium with seta; merus 3.65 L:W, 0.9x carpus, with ventrodistal fine spine; carpus 4.1 L:W, 0.85x propodus, with four distal spines (two long fine, one short fine, one robust); propodus 7.0 L:W, 3.5x dactylus, with two distodorsal spines (fine and robust) and one subdistal ventral spine; dactylus 6.4 L:W, 0.5x unguis; unguis and dactylus about 0.9x propodus.

Pereopod-2 (Fig. 30D) coxa with seta; basis 5.1 L:W; ischium with ventral seta; merus 3.3 L:W, 0.8x carpus, with ventrodistal fine spine; carpus 4.1 L:W, 0.95x propodus with four fine distal spines (three long and one short); propodus 5.6 L:W and 2.7x dactylus, with subdistal ventral spine and two subdistal dorsal spines (robust and fine), ventral margin with microtrichia; dactylus 7.0 L:W, 0.7x unguis; dactylus and unguis 0.9x propodus.

Pereopod-3 (Fig. 30E) similar to pereopod-2, but basis with midventral long penicillate seta.

Pereopod-4 (Fig. 30F) basis 4.3 L:W, naked; ischium with two ventral setae; merus 2.7 L:W, as long as carpus, with two ventrodistal serrate spines; carpus 2.7 L:W, 0.6x propodus, with four distal serrate spines and rod-like seta dorsodistally; propodus 7.7 L:W, with penicillate middorsal seta, two ventrodistal serrate spines and one serrate dorsodistal spine; dactylus 8.0 L:W; unguis just shorter than dactylus.

Pereopod-5 (Fig. 30G) similar to pereopod-4, but propodus without dorsodistal penicillate seta.

Pereopod-6 (Fig. 30H) similar to pereopod-5, but propodus with three dorsodistal spines.

Uropod (Fig. 30I) basal article 1.1 L:W; exopod with two articles, 0.7x endopod, article-1 2.5 L:W, with distal seta, article-2 3.7 L:W, with one long and one short distal setae; endopod with two articles, article-1 3.1 L:W, with two penicillate distal setae; article-2 3.5 L:W, with three long one short and two penicillate distal setae.

**Description of adult male**, length 3.4 mm. Body (Fig. 28C,D) slender, 8.5 L:W; cephalothorax as long as wide, 2.5x pereonite-1. Pereonites 1−6: 0.5, 0.6, 0.9, 0.8, 1.1 and 0.8 L:W, respectively; pereonites 1−3 wider proximally, pereonites 4 and 5 wider medially, pereonite-6 wider distally. Pleon 0.3 of total body length. All pleonites the same size, 0.4 L:W, with rounded lateral margins, without hyposphaenium. Pleotelson 1.2x pleonite-5, distal spine 2.0x pleotelson proximal part.

Antennule (Fig. 31A) article-1 2.1 L:W, 1.75x article-2, with four penicillate and two simple subdistal or distal setae, and four penicillate proximal setae; article-2 1.2 L:W, 2.4x article-3, with two simple and one penicillate setae subdistally; article-3 0.7 L:W, 1.5x article-4, with distal seta; article-4 0.5 L:W, with numerous aesthetascs arranged in transversal row; article-5 as long as wide, with numerous long aesthetascs arranged in transversal row; article-6 2.7 L:W, with a few aesthetasc distally; article-7 4.6 L:W, with five setae distally.

Antenna (Fig. 31B) 0.6 times length of antennule; article-1 fused with body; article-2 1.7 L:W, 1.8x article-3, with distodorsal seta; article-3 L:W, as long as article-4, with distodorsal seta; article-4 1.6 L:W, 0.3x article-5, with penicillate seta distally; article-5 4.7 L:W, 1.2x article-6, with three simple and five penicillate setae distally; article-6 7.0 L:W, naked with suture at 1/3 of the length and distal seta; article-7 as long as wide, with five long and one short distal setae (Fig. 31B’).

Mouthparts. Labrum hood-shape, naked (not illustrated); mandible, maxillule endite, and labium reduced to small plates (not illustrated). Maxilla (Fig. 31D) semiround. Maxilliped (Fig. 31C) basis 1.3 L:W, naked; palp article-1 1.4 L:W, naked; article-2 as long as wide, with three inner setae; article-3 2.0 L:W, with four inner setae (three long, one short); article-4 1.8 L:W, with four distal or subdistal inner setae and one outer seta; maxilliped endites (Fig. 31C’) separate, short and narrow, distally with two setae and two slender gustatory cusps. Epignath (Fig. 31E) distally narrowed and pointed.

Cheliped (Fig. 32A,A’) sclerite large; basis 1.5 L:W, naked; merus wedge-shape, with ventral seta; carpus little wider medially, 1.7 L:W, with two ventral setae, one subdistal and one subproximal dorsal setae; chela slender, 1.2x carpus, 1.6 L:W; propodus (palm) with eleven setae on inner side; fixed finger with irregular inner margin; with three inner setae and two ventral setae; dactylus 4.7 L:W, almost straight, with at least two calcified spines on inner margin and subproximal seta on inner side.

Pereopod-1 (Fig. 32B) basis 6.8 L:W, naked; ischium with ventral seta; merus 3.0 L:W, 0.75x carpus, with ventrodistal fine spine; carpus 4.4 L:W, 0.9x propodus, with four distal spines; propodus 6.0 L:W, 3.0x dactylus, with two subdistal spines dorsally and one subdistal ventral spine; dactylus 0.8x unguis, with seta reaching end of dactylus; unguis and dactylus about 0.8x propodus.

Pereopods 2 and 3 (Fig. 32C,D) as pereopod-1.

Pereopod-4 (Fig. 32E) basis 5.1 L:W, with dorsoproximal penicillate seta; ischium with two ventral setae; merus 2.5 L:W, as long as carpus, with two ventrodistal spines; carpus 3.1 L:W, 0.6x propodus, with four distal spines and dorsodistal seta; propodus 9.5 L:W, with penicillate middorsal seta, two ventrodistal serrate spines and one serrate dorsodistal spine; dactylus 10.0 L:W; unguis 1.1x dactylus, 0.7x propodus.

Pereopod-5 (Fig. 32F) similar to pereopod-4, but propodus without dorsodistal penicillate seta.

Pereopod-6 (Fig. 32G) similar to pereopod-4, but basis without penicillate setae; propodus with three dorsodistal spines.

Pleopods (Fig. 32H) basal article 2.1 L:W, exopod 0.9x endopod, 5.5 L:W, with nine distal and ventrodistal and one dorsal long serrate setae; endopod 5.5 L:W, with 14 distal and ventrodistal serrate setae.

Uropod (Fig. 32I) basal article 0.9 L:W; exopod with two articles, 0.6x endopod, article-1 2.6 L:W, with distal seta, article-2 5.0 L:W, with two distal setae; endopod with three articles, article-1 1.5 L:W, with eight penicillate distal setae; article-2 2.1 L:W, with two penicillate distal setae; article-3 4.3 L:W, with five simple and two penicillate distal setae.

### Distribution

*Paranarthrurella samba* sp. nov. is known so far only from the type locality off Brazilian coast, at depth of 3459 m.

### Remarks

*Paranarthrurella samba* is one of five *Paranarthrurella* species with an elongate body habitus and swollen pleotelson with its apex directed downward. In contrast to *P. polonez* has smooth lateral margins on the pleonites. The species can be distinguished from *P. spinimaxillipeda* by the length of pereonite-1 that is 1.7 times as long as wide (clearly shorter in *P. spinimaxillipeda*). Finally, the new species is distinguished from *P. dissimilis* by lack of strongly serrate dorsodistal spine in pereopods 4–6 propodus (present in *P. dissimilis*, see Lang 1972, p. 234).

*P. samba* has clearly less robust appendages (cheliped, pereopods) than *P. kizomba*, and the merus of pereopod-1 is subequal to the carpus (merus shorter than carpus in pereopod-1 of *P. kizomba*). Moreover *P. samba* has slender setae on antenna articles 2–3 (short and robust in *P. kizomba*) and *P. kizomba* has a long seta on maxilliped palp article-2, which is not observed in *P. samba*.

***Paranarthrurella spinimaxillipeda*** (**Larsen and Araújo-Silva, 2014)**

*Cheliasetosatanais spinimaxillipeda:* Larsen and Araújo-Silva, 2014: 970–973, Figs 1–4 ^18^.

### Diagnosis

Body long (>8 L:W). Pereonite-1 0.5 L:W. Pleonites lateral margin smooth (not pointed). Pleonites 2–4 without hyposphaenia. Pleon rounded and swollen, apex small. Cheliped carpus 1.4 L:W. Pereopod-1 merus with one spine. Pereopods 4–6 carpus with four spines. Pereopods 4–6 propodus dorsodistal spine finely serrate. Uropod endopod 1.4x exopod.

### Remarks

From six species of *Panarthrurella* which have long body, *P. spinimaxillipeda* can be distinguished by: rounded pleotelson and small and pointed downward apex (large and pinted backward in *P. caudata*); smooth lateral margins of pleonites (pointed in *P. polonez*); by presence of weakly serrated spines in propodus of pereopods 4–6 (strongly serrated in *P. dissimilis*) and by short pereonite-1 that is 0.6 W/L (0.8 W/L in *P. samba*) and presence of a long seta on the basis of the maxilliped (no setae in *P. samba*). For differences between *P. spinimaxillipedes* and *P. kizomba*, see remarks above (*P. kizomba*).

### Distribution

The species is known from the Central Pacific, in the Manganese Nodule Province between 4259–4261 m depth.

***Paranarthrurella tango*** **Błażewicz and Jóźwiak sp. nov.**

Figures 33–38

**Figure 33:**
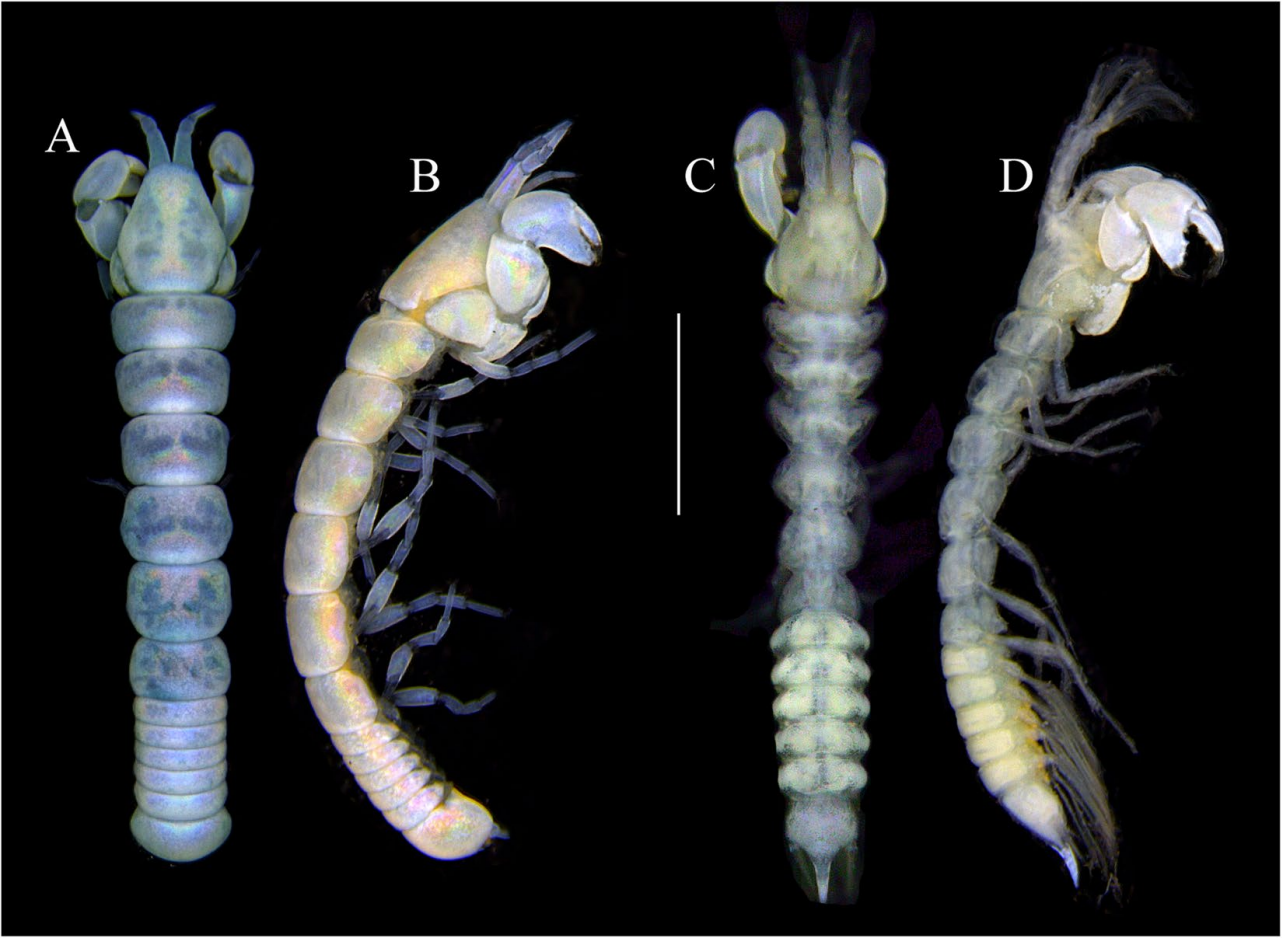
*Paranarthrurella tango* sp. nov. Holotype, neuter (MCZ:IZ:48363) (**A**) dorsal and (**B**) lateral. Paratype male (MCZ:IZ:149570) (**C**) dorsal and (**D**) lateral, respectively. Scale 1 mm.

**Figure 34:**
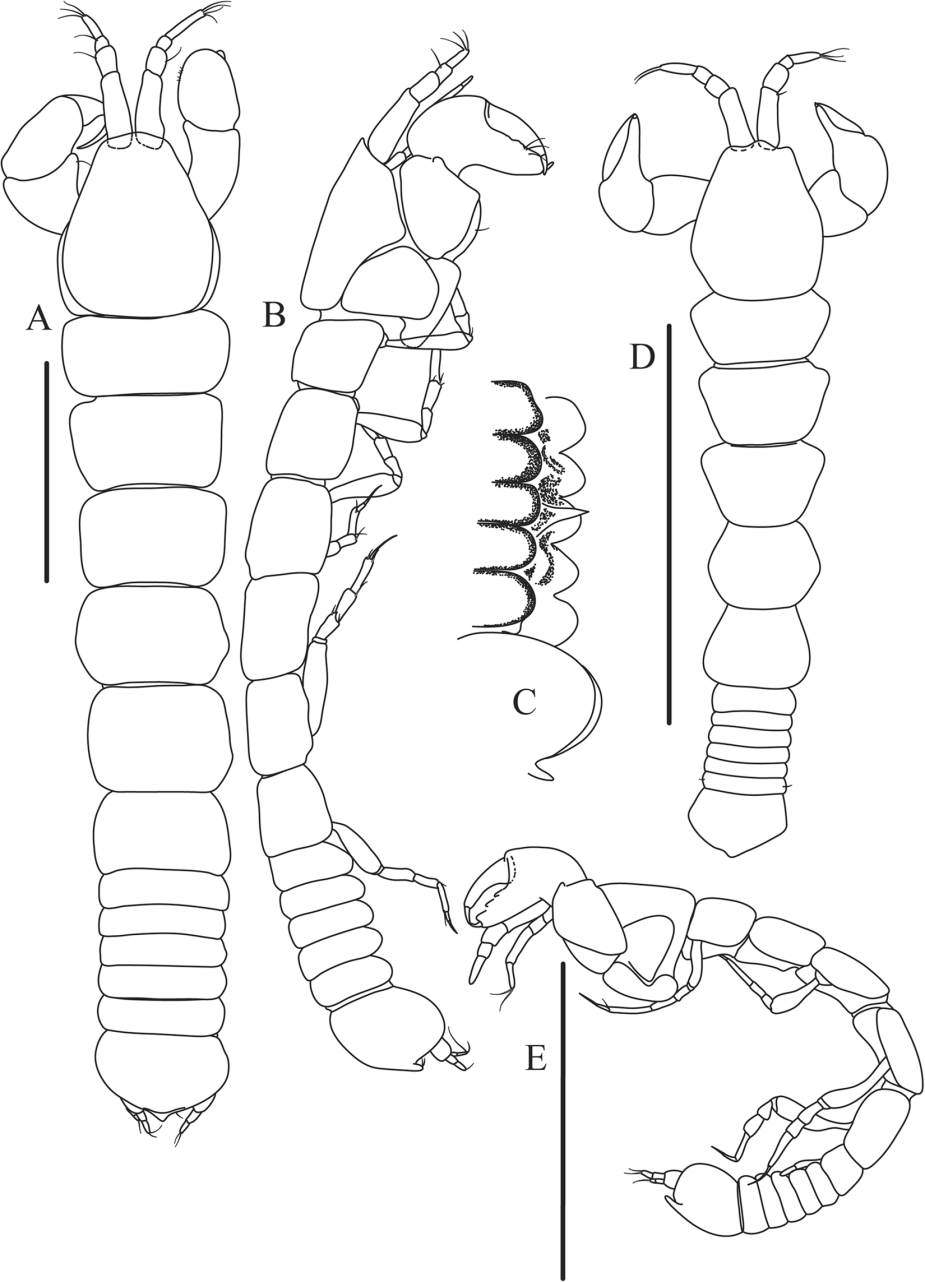
*Paranarthrurella tango* sp. nov. Holotype neuter (MCZ:IZ:48363). (**A**) Dorsal and (**B**) lateral. (**C**) Pleon, lateral. (**D**) Manca-2 dorsal (**E**) manca-3 lateral, respectively (MCZ:IZ:149571). Scale 1 mm.

**Figure 35:**
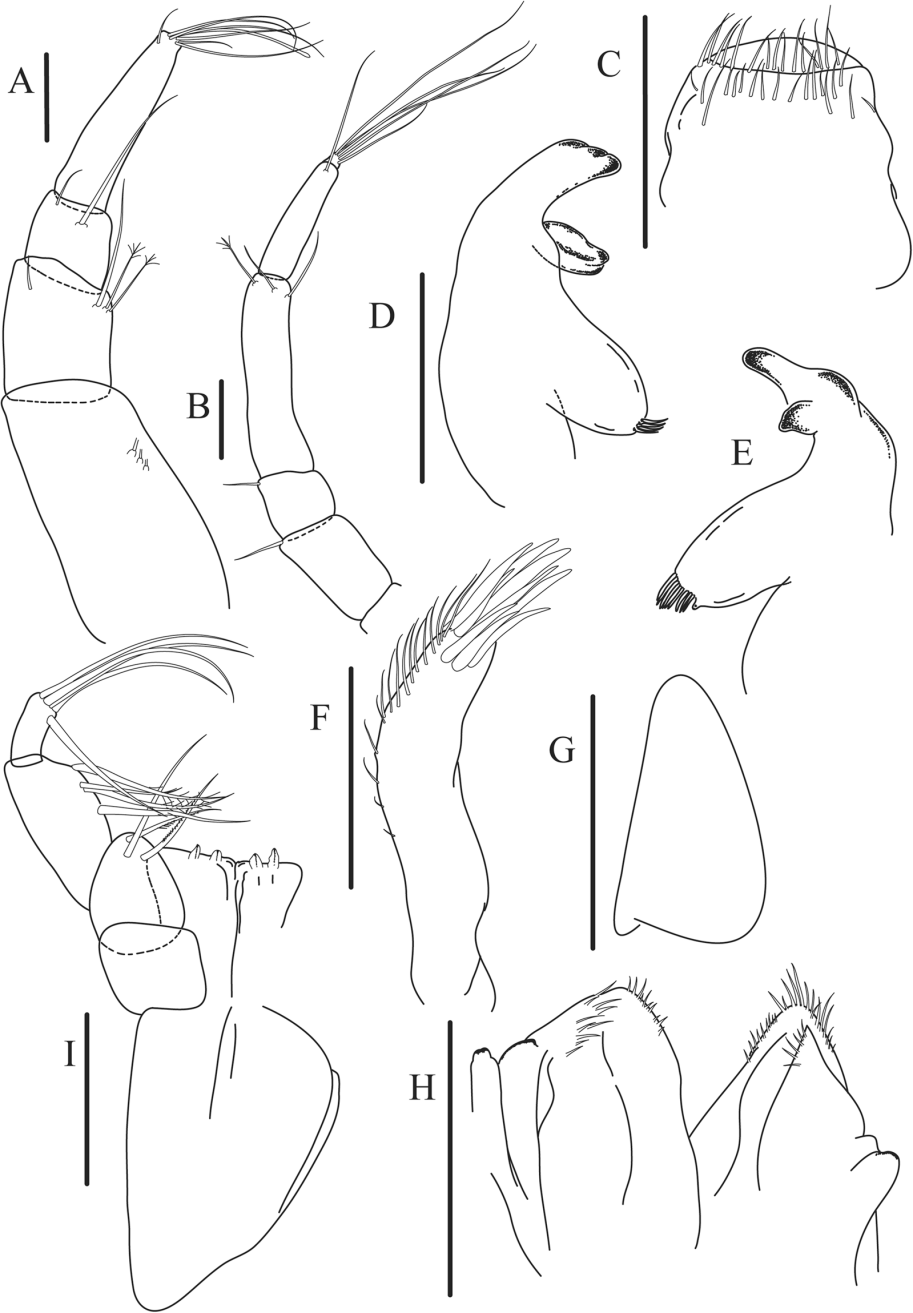
*Paranarthrurella tango* sp. nov. Paratype, neuter (MCZ:IZ:149548) (**A**) Antennule. (**B**) Antenna. (**C**) Labrum. (**D**) Mandible, left. (**E**) Mandible, right. (**F**) Maxillula. (**G**) Maxilla. (**H**) Labium. (**I**) Maxilliped. Scale 0.1 mm.

**Figure 36:**
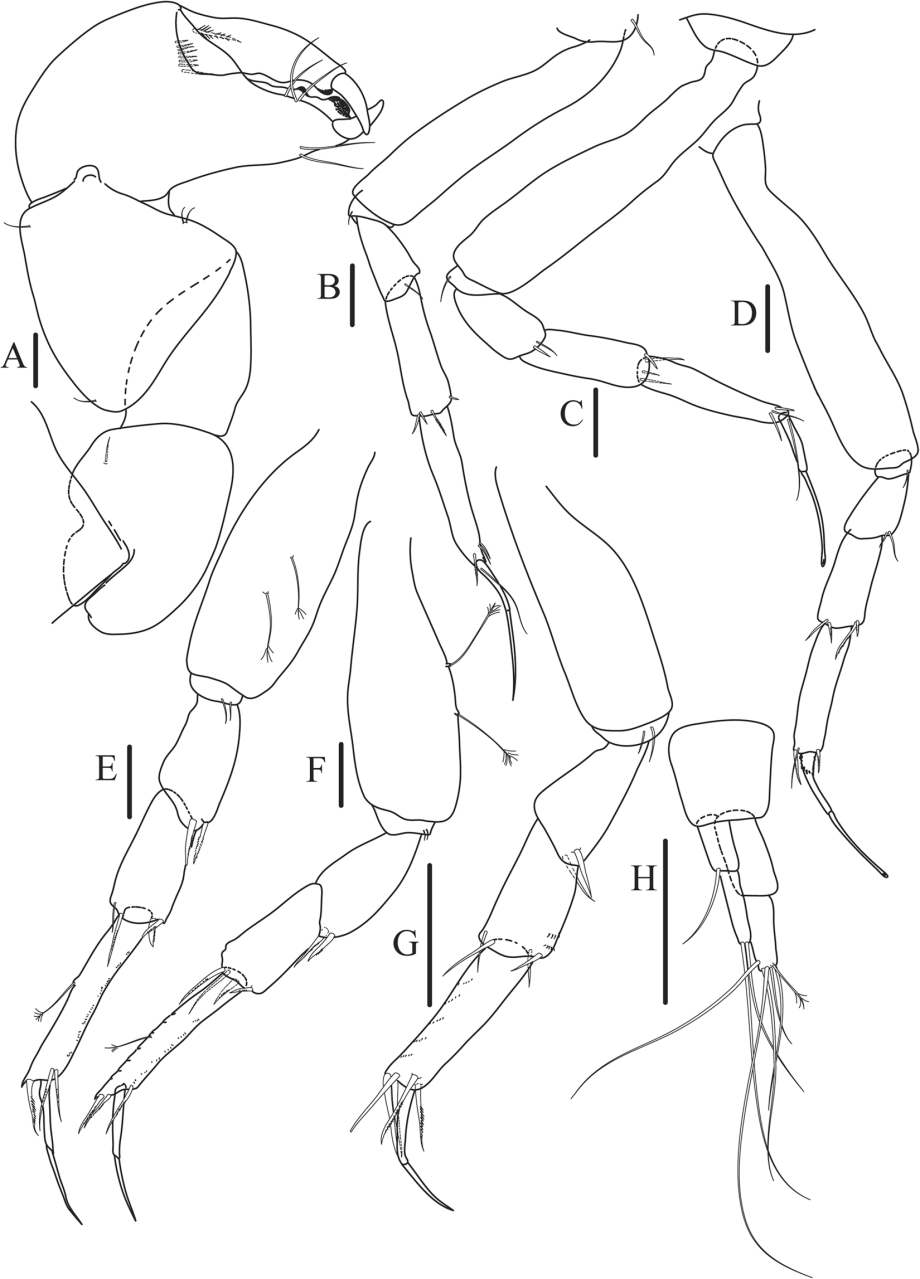
*Paranarthrurella tango* sp. nov. Paratype, neuter (MCZ:IZ:149548) (**A**) Cheliped. (**B**–**G**) Pereopod 1–6, respectively. (**H**) Uropod. Scale 0.1 mm.

**Figure 37:**
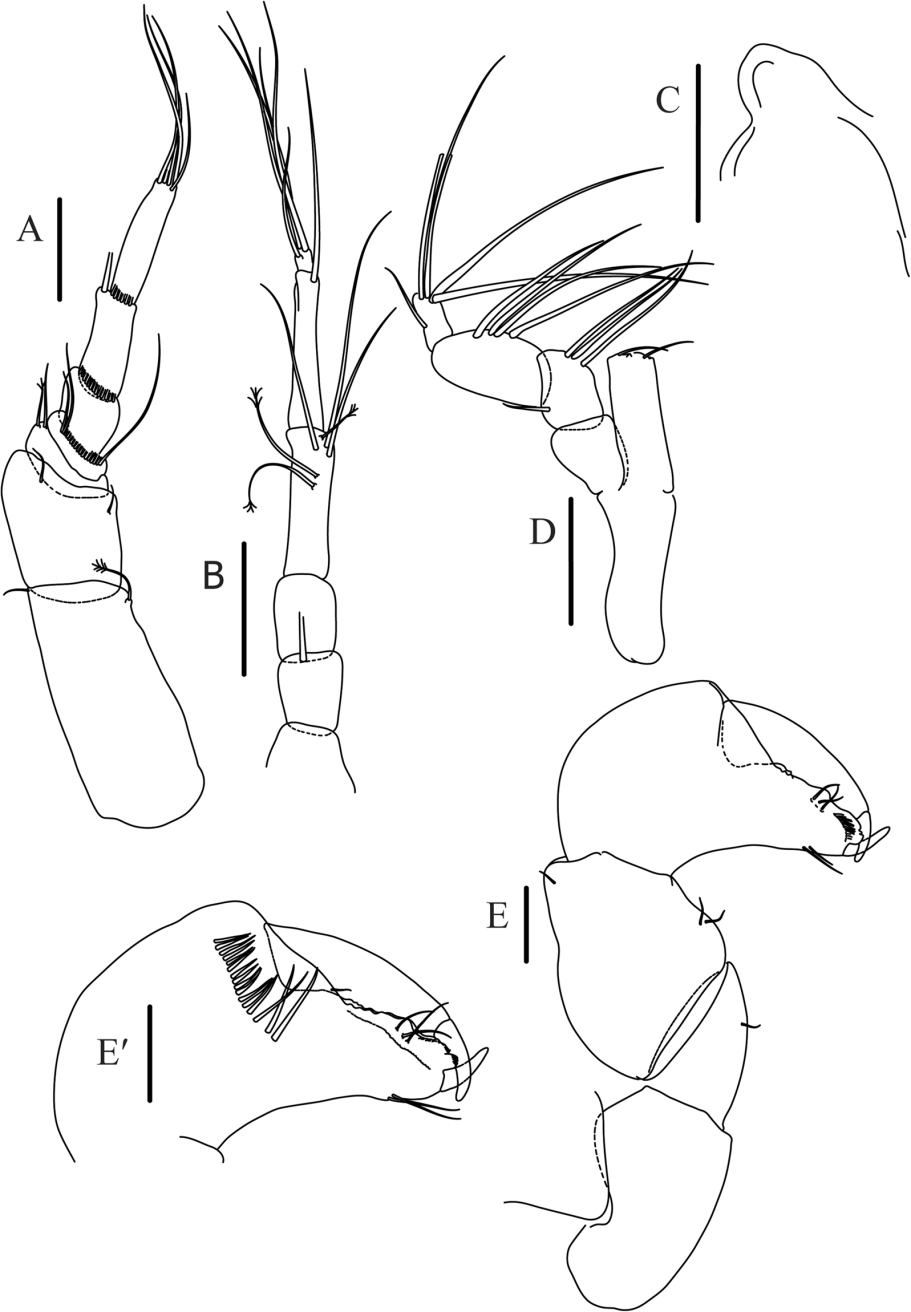
*Paranarthrurella tango* sp. nov. Paratype, male (MCZ:IZ:149572) (**A**) Antennule. **(B**) Antenna. (**C**) Labrum. (**D**) Maxilliped. (**E**) Cheliped. (**E’**) Chela, inner side details. cale 0.1 mm.

**Figure 38:**
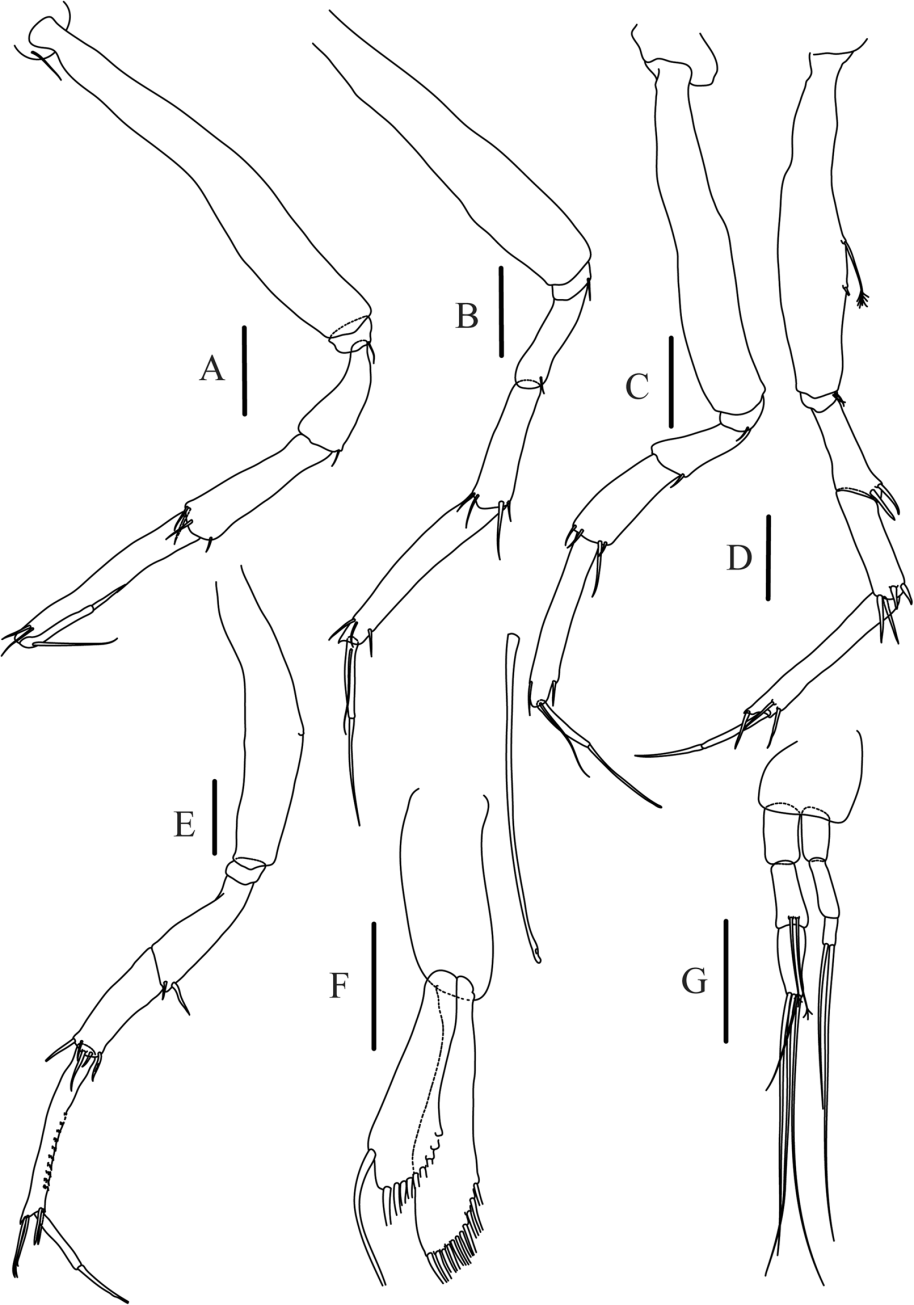
*Paranarthrurella tango* sp. nov., paratype, male (MCZ:IZ:149572) (**A**–**E**) Pereopod 1–4 and 6, respectively. (**F**) Pleopod. (**G**) Uropod. Scale 0.1 mm.

### Material examined

Holotype, neuter (4.8 mm BL), (MCZ:IZ:48363), RV *Atlantis II*, Cr. 60, St. 262.

Paratypes: two juvenile males (3.4–3.8 mm BL), (MCZ:IZ:149569); male (2.8 mm BL, partly dissected), (MCZ:IZ:149570); four mancas-2 (1.7–2.3 mm BL), two mancas-3 (1.8–2.1 mm BL), (MCZ:IZ:149571); male (dissected), (MCZ:IZ:149572), two females (dissected), (MCZ:IZ:149548 and MCZ:IZ:149585), the same locality as holotype; two neuters, three mancas-2, one male(?) (MCZ:IZ:48362), RV *Atlantis II*, Cr. 60, St. 245.

### Diagnosis

Female: Body short (<6.5 L:W). Pereonite-1 0.5 L:W. Pleonite 2 and 4 without hyposphaenium; pleonite-3 with small, pointed hyposphaenium. Cheliped carpus 1.2 L:W. Pereopod-1 merus with one fine spine. Pereopods 4–6 carpus with four fine spines. Pereopods 4–6 propodus dorsodistal spine/spines finely serrate. Uropod endopod 1.2x exopod.

### Etymology

The species name referring to the Argentinian tango, a social dance originating in the suburbs of Buenos Aires and Montevideo. Noun in apposition.

**Description of neuter**, length 4.5 mm. Body (Figs 33A,B and 34A,B) short, 5.9 L:W; cephalothorax 1.3 L:W, 2.4x pereonite-1. Pereonites 1−6: 0.5, 0.6, 0.7, 0.6, 0.7 and 0.6 L:W, respectively; all pereonites wider than long, margins weakly rounded. Pleon 0.25 of total body length. All pleonites the same size, 0.25 L:W. Pleotelson 2.5x pleonite-5, rounded, with small and pointed downward apex.

Antennule (Fig. 35A) article-1 2.1 L:W, 2.5x article-2, with three middle penicillate setae (broken); article-2 1.3 L:W, 2.0x article-3, with two simple and two penicillate distal setae; article-3 0.9 L:W, 0.4x article-4, with long and short distal setae; article-4 3.6 L:W, with five long and two short setae, and with aesthetasc, distally.

Antenna (Fig. 35B) 0.85 times length of antennule; article-1 fused with body; article-2 1.7 L:W, 1.8x article-3, with distal seta, situated in right angle to axis of the article; article-3 0.9 L:W, 0.3x article-4, with distal seta, situated in right angle to axis of the article; article-4 4.75 L:W, 1.6x article-5, with two simple and one penicillate setae distally; article-5 4.0 L:W, with distal seta; article-6 as long as wide, with four long and one short distal setae.

Mouthparts. Labrum (Fig. 35C) large elongate; distally obtuse and with sparsely distributed robust setae. Right mandible (Fig. 35E) incisor distally extended, with smooth edge; *lacinia mobilis* fused with incisor, prominent, pointed, with simple edge; molar lobeform, distally weakly rounded, with eleven weak, finger-shape setae. Left mandible (Fig. 35D) incisor narrow, distally with two perpendicular grooves; *lacinia mobilis* well developed; molar with four small distal setae. Maxillule endite (Fig. 35F) with nine strong distal spines of various length distally; numerous setae along outer margin; palp not observed. Maxilla (Fig. 35G) semitriangular. Labium (Fig. 35I) with two lobes; inner lobe with spines on distoinner margin and with strong setae distally; outer lobe bilobed, smaller than inner lobe. Maxilliped (Fig. 35H) basis 1.6 L:W, naked; palp article-1 1.1 L:W, naked; article-2 1.1 L:W, with three serrate inner setae; article-3 2.0 L:W, with four inner sparsely serrate setae; article-4 2.3 L:W, with five distal and subdistal inner setae; no outer seta observed; maxilliped endites separate, narrow, 2.25 L:W, distally with two slender gustatory cusps; no distal seta observed. Epignath not seen.

Cheliped (Fig. 36A) sclerite large semitriangular; basis 1.9 L:W, with one subdistal dorsal seta; merus wedge-shape, with ventral seta (broken); carpus 1.2 L:W, with two ventral setae, one subdistal and one subproximal dorsal setae; chela slender, 1.2x carpus, 1.7 L:W; propodus (palm) seta near dactylus insertion not seen, eight setae on inner side (one long, seven short); fixed finger with robust distal spine (unguis), with middle (smaller) and distal (bigger) chitinized teeth on cutting margin and two ventral setae; cutting margin distally well calcified, with three setae; fixed finger and dactylus unifacial; dactylus almost straight, with three spines on cutting margin and subproximal serrate seta on inner side.

Pereopod-1 (Fig. 36B) longer than pereopods 2–3; coxa with seta; basis 4.75 L:W, naked; ischium with seta; merus 1.7 L:W, 0.7x carpus with distal fine spine; carpus 2.6 L:W, 0.7x propodus, with four short distal spines; propodus 4.7 L:W, 2.7x dactylus, with one subdistal ventral serrate spine and two short subdistal dorsal spines (one fine); dactylus 6.4 L:W, 0.6x unguis, with seta reaching 0.3 of unguis; unguis and dactylus about 0.9x propodus.

Pereopod-2 (Fig. 36C) basis 4.5 L:W, naked; ischium with ventral seta; merus 1.7 L:W, 0.75x carpus, with two spines (one fine) distally; carpus 2.6 L:W, 0.75x propodus, with four distal spines; propodus 4.7 L:W and 2.7x dactylus, with subdistal ventral spine and two subdistal spines (one fine) dorsally; dactylus 6.4 L:W, 0.6x unguis, with proximal seta.

Pereopod-3 (Fig. 36D) similar to pereopod-2, but dactylus proximal seta not seen.

Pereopod-4 (Fig. 36E) basis 2.5 L:W, with two penicillate setae on ventral margin; ischium with two ventral setae; merus 1.7 L:W, 0.9x carpus, with two ventrodistal serrate spines; carpus 2.0 L:W, 0.7x propodus, with three serrate spines and fine spine distally; propodus 7.3 L:W, with penicillate middorsal seta, two ventrodistal long serrate spines and one serrate dorsodistal spine; dactylus 8.7 L:W; unguis 0.8x dactylus; together 0.8x propodus.

Pereopod-5 (Fig. 36F) as pereopod-4.

Pereopod-6 (Fig. 36G) similar to pereopod-5, but basis naked; propodus without dorsodistal penicillate seta and with three dorsodistal serrate spines.

Uropod (Fig. 36H) basal article 1.1 L:W; exopod with two articles, 0.8x endopod, article-1 1.8 L:W, with distal seta, article-2 5.0 L:W, with two distal setae; endopod with two articles, article-1 1.9 L:W; article-2 2.8 L:W, with five simple and one penicillate setae distally.

**Description of adult male**, length 3.4 mm. Body (Fig. 33C,D) slender, 8.5 L:W; cephalothorax as long as wide, 2.5x pereonite-1. Pereonites 1−6: 0.5, 0.6, 7.9, 0.9, 1.1 and 0.8 L:W, respectively; pereonites 1–3 wider proximally, pereonites 4 and 5 wider medially, pereonite-6 wider distally. Pleon 0.15 of total body length. All pleonites the same size, 0.4 L:W, with rounded lateral margins, without hyposphaenium. Pleotelson 3.2x pleonite-5, apex 2.0x pleotelson proximal part.

Antennule (Fig. 37A) article-1 2.3 L:W, 2.1x article-2, with one penicillate and one simple distal setae; article-2 1.2 L:W, 3.9x article-3, with two short distal setae; article-3 0.4 L:W, 1.8x article-4, with two setae distally; article-4 0.2 L:W, 0.3x article-5, with numerous long aesthetascs arranged in transversal row; article-5 0.8 L:W, 0.5x article-6, with numerous long aesthetascs arranged in transversal row; article-6 2.4 L:W, 0.8x article-7, with few aesthetascs distally, article 7 4.0 L:W, with five setae distally.

Antenna (Fig. 37B) 0.6 times of antennule length; article-1 fused with body; article-2 1.4 L:W, 0.9x article-3, with distal seta (broken); article-3 1.4 L:W, 0.6x article-4, no seta observed; article-4 3.6 L:W, 0.9x article-5, with three simple and three penicillate distal and subdistal setae; article-5 7.0 L:W, with simple distal seta; article-6 as long as wide, with five distal setae.

Mouthparts. Labrum (Fig. 37C) hood-shape, naked; mandible, maxillule endite, maxilla and labium reduced to small plates (not illustrated). Maxilliped (Fig. 37D); palp article-1 1.2 L:W, naked; article-2 1.3 L:W, with three inner and one outer setae; article-3 1.7 L:W, with four long inner setae; article-4 2.9 L:W, with five distal and subdistal inner setae and outer seta; maxilliped endites separated, narrow, distally with two setae and at least one gustatory cusp. Epignath not seen.

Cheliped (Fig. 37E) sclerite semitriangular; basis 2.1 L:W; merus wedge-shape, with small ventral seta; carpus little wider medially, 1.5 L:W, with two ventral setae, one dorsodistal seta, dorsoproximal seta not seen; chela slender, 0.7x carpus, 1.6 L:W; propodus (palm) with 16 setae on inner side (Fig. 37E’); fixed finger with calcified inner margin distally, divided by transversal grooves, with three setae on cutting edge and two ventral setae; dactylus 5.3 L:W, unguis bent downward.

Pereopod-1 (Fig. 38A) coxa with seta; basis 7.6 L:W, naked; ischium with seta; merus 2.6 L:W, 0.7x carpus, with ventrodistal small spine; carpus 4.2 L:W, 0.8x propodus, with four fine distal spines (one small); propodus 7.9 L:W, 2.5x dactylus, with two unequal, fine dorsodistal spines (ventral spine not observed); dactylus 9.0 L:W, with proximal seta; unguis about half as long as dactylus.

Pereopod-2 (Fig. 38B) similar to pereopod-1, but propodus 5.9 L:W, with subdistal ventral spine distally; dactylus 7.8 L:W, 0.6x unguis; dactylus and unguis subequal propodus.

Pereopod-3 (Fig. 38C) similar to pereopod-2, but propodus with one subdistal spine dorsally.

Pereopod-4 (Fig. 38D) basis 5.6 L:W, with two dorsoproximal penicillate seta; ischium with two ventral setae; merus 3.1 L:W, as long as carpus, with two ventrodistal spines; carpus 3.7 L:W, 0.7x propodus, with four distal spines; propodus 8.3 L:W, with two ventrodistal spines and one dorsodistal spine; dactylus 10.6 L:W; unguis 1.1x dactylus, combined 0.8x propodus.

Pereopod-5 (not illustrated) as pereopod-4.

Pereopod-6 (Fig. 38E) similar to pereopod-4, but propodus with three dorsodistal spines.

Pleopods (Fig. 38F) basal article 2.5 L:W, exopod 0.8x endopod, 5.2 L:W, with nine distal and ventrodistal plumose setae and dorsal long plumose seta; endopod 5.5 L:W, with eleven distal and ventrodistal plumose setae.

Uropod (Fig. 38G) basal article 0.9 L:W; exopod with three articles, 0.8x endopod, article-1 2.1 L:W, naked, article-2 3.5 L:W, naked; article-3 2.4 L:W, with two distal setae; endopod with three articles, article-1 1.8 L:W, naked; article-2 2.5 L:W, with two penicillate distal setae; article-3 2.9 L:W, with three long and one short distal setae.

### Distribution

The species is known only from the type locality located off the Argentinian coast at the depth 2440–2707 m.

### Remarks

*Paranarthrurella tango* sp. nov. is the third species with short body and hyposphaenium only on pleonite-3; this hyposphaenium is small, distinguishing it from *P. arctophylax* (see remarks page 15). Another species with a small hyposphaenium on pleonite-3 is *P. rocknroll*. Those two species can be distinguished by character of the distal seta on the propodus of pereopods 4–6 that is strongly serrate in *P. rocknroll* and serrated in *P. tango*.

***Paranarthrurella voeringi*** **(G.O. Sars, 1877)**

*Tanais voringi*: G.O. Sars, 1877: 2, 347–370^6^.

*Cryptocope voringii:* G.O. Sars, 1882: 7, 50–51^7^; G.O. Sars, 1885: 6, 74–78, pl.VII^61^; Hansen, 1913: 3, 109–110, pl. X^8^.

*Tanais voeringi:* Sieg, 1980: 537, 11–12^82^.

*Leptognathia voeringi:* Sieg, 1983: 356^72^; Sieg 1986: 170^73^.

*Biarticulata voeringi:* Larsen and Shimomura, 2007: 19^13^.

*Paranarthrurella voeringi:* Jóźwiak *et al*., 2009: 59 [partially]^16^.

*Paranarthrurella voeringi:* Błażewicz-Paszkowycz and Bamber, 2011: 31^83^.

### Diagnosis

Body short (<6.5 L:W). Pereonite-1 0.4 L:W. Pleonite 2–4 with distinct, directed backward hyposphaenium (hypospahaenium on pleonite-4 smaller than on preonites 2–3). Cheliped carpus 1.2 L:W. Pereopod-1 merus with two spines. Pereopods 4–6 carpus with four spines. Pereopods 4–6 propodus dorsodistal spine finely serrate. Uropod endopod 1.2x exopod.

### Distribution

**Figure 39:**
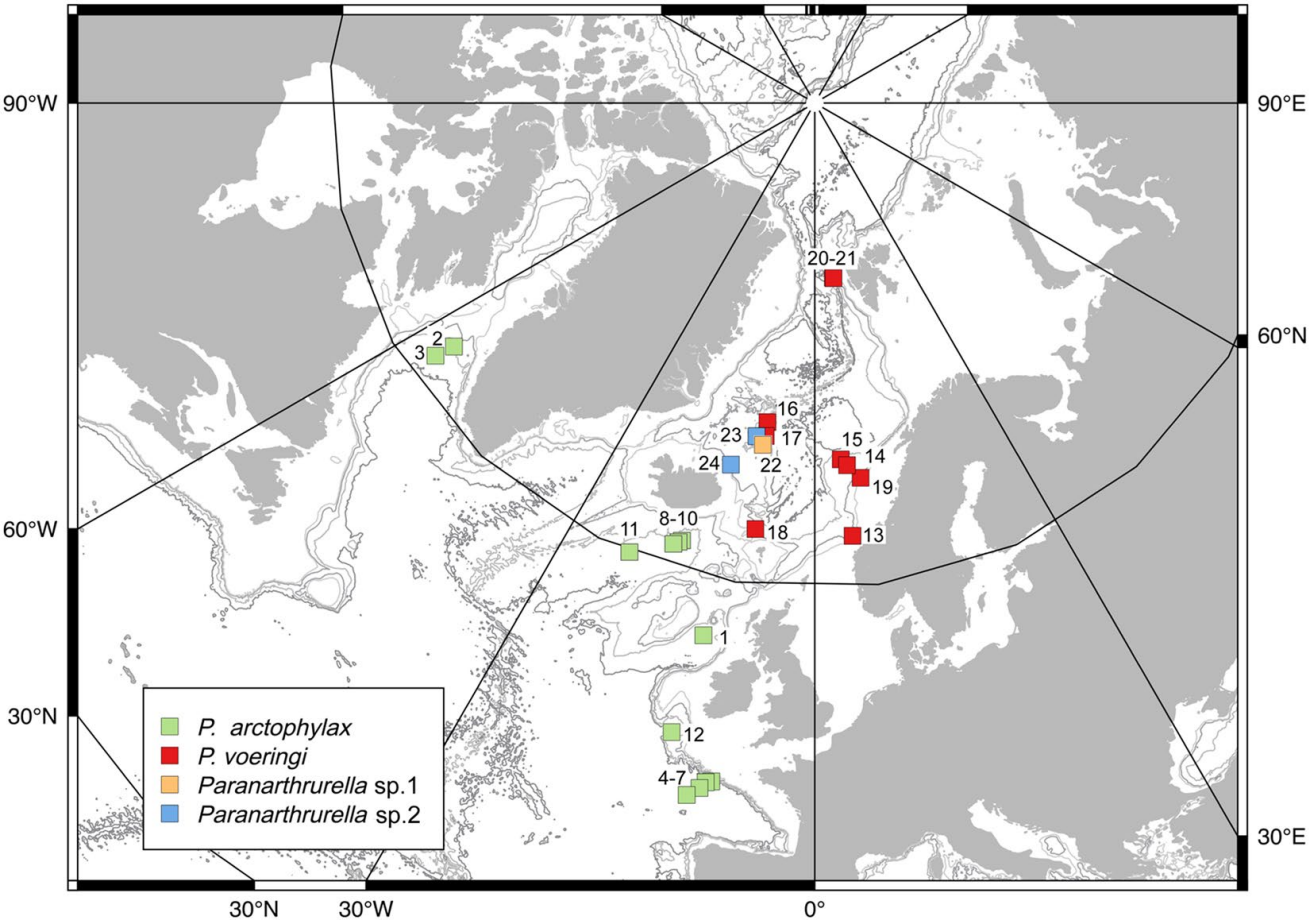
Distribution of the septentrional *Paranarthrurella* species (*P. arctophylax, P. voeringi, Paranarthrurella* sp.1 and *Paranarthrurella* sp.2) in the North Atlantic, based on the literature data (see Table 5) and current study.

The species was primarily described by G.O. Sars in 1877 from off Storeggen Bank at 763 m depth the 29th June 1876 (St 31)^6^. On a second cruise carried out in 1877, a few specimens were obtained at 2 other localities (St 124, 248), off coast of Helgeland from 640 and 1423 m depth, respectively. Latter the species was recorded by Hansen^8^ in the North Atlantic during the Ingolf expedition, northwestern Faeroes (St 138) and southern Jan Mayen (St 116, 117), in the depth range from 678.5 to 1835 m. Recently, the species was rediscovered in Arctic waters at the observatory HAUSGARTEN, in the eastern Fram Strait at two stations at the depth 1273–1300 m, and in the neighbourhood of the type locality at 742 m depth^16^. Its distribution depth ranges between 640–1835 m (Table 5, Fig. 39).

### Remarks

*P voeringi* is the only species that has hyposphaenia on pleonites 2 to 4.

***Paranarthrurella*** **sp.1**

### Material examined

Three individuals: ind. ITan159 (ZMH K-55996), ind. ITan163 (ZMH K-55997); ind ITan164 (ZMH K-55998), RV *Meteor*, IceAGE1, St. 1155.

### Remarks

See remarks under *Paranarthrurella* sp.2 (below).

***Paranarthrurella*** **sp.2**

### Material examined

Three individuals: ind. ITan 158 (ZMH K-55999), ind. ITan160 (ZMH K-56000), ind. ITan161 (ZMH K-56001) RV *Meteor*, IceAGE1, St. 1155; two individuals: ITan170 (ZMH K-56002), ind ITan162 (ZMH K-56003), RV *Meteor*, IceAGE1, St. 1191.

### Remarks

*Paranarthrurella* sp.1 and *Paranarthrurella* sp.2 are genetically discriminated species, but both are morphologically identical with *P. voeringi*. The latter species was considered cold-water taxon^8^ recorded in relatively shallower waters (<2000 m) than the congener – *P. arctophylax*, which although known from the depth (>2000 m), was considered a “warm” water species (Table 5, Fig. 39). In the material studied by us, all of the individuals had distinctive hyposphaenia on pereonites from 2–4, thus morphologically are similar to *P. voeringi* and both occur at the depths typical for *P. voeringi* (St 1155 and 1191, depth 1577–1578 and 2177–2174 m, respectively; Table 5). Generally, we might assume that one of our two genetically distinguished species is *P. voeringi*. However, the decision which of them would truly represent the latter requires further molecular investigations which will include individuals of *P. voeringi* from a type locality.

**Identification key** **to females of** ***Paranarthrurella*** **species**

(modified after Jóźwiak *et al*.^16^; distribution data according to current study and: Sars G.O.^6,61^; Norman and Stebbing^12^; Hansen^8^; Kudinova-Pasternak^2^; Lang^9^; Holdich and Bird, 1985^81^; Jóźwiak *et al*.^5^; Błażewicz-Paszkowycz and Bamber^83^; Larsen and Araújo-Silva^18^).

- Pleotelson rectangular, apex large directed backward. Pereopods 4–6 carpus with three spines. . . . . . . . . . . . . . . . . . . . . . . . . . . . . . . . . . . . . . . . . . . . . . . . . . . . . . . . . . . . . . . *P. caudata* (Kudinova-Pasternak, 1965)

Bougainville Trench; 7947–8006 m

- Pleotelson round, apex small pointed downward. Pereopods 4–6 carpus with four spines . . . . . . . . . . . . . . . . .2

- Body short (<6 L:W) . . . . . . . . . . . . . . . . . . . . . . . . . . . . . . . . . . . . . . . . . . . . . . . . . . . . . . . . . . . . . . . . . . . . . . . . . . . . . . 3

- Body long (>8 L:W) . . . . . . . . . . . . . . . . . . . . . . . . . . . . . . . . . . . . . . . . . . . . . . . . . . . . . . . . . . . . . . . . . . . . . . . . . . . . . . . 8

- Hyposphaenium-3 well developed . . . . . . . . . . . . . . . . . . . . . . . . . . . . . . . . . . . . . . . . . . . . . . . . . . . . . . . . . . . . . . . . . . 4

- Hyposphaenium-3 small . . . . . . . . . . . . . . . . . . . . . . . . . . . . . . . . . . . . . . . . . . . . . . . . . . . . . . . . . . . . . . . . . . . . . . . . . . . 7

- Hyposphaenium-4 present . . . . . . . . . . . . . . . . . . . . . . . . . . . . . . . . . . . . . . . . . . . . . . . . . . *P. voeringi* (G.O. Sars, 1877)

NE Atlantic (Norwegian Sea) and Arctic; 640–1835 m

- Hyposphaenium-4 absent . . . . . . . . . . . . . . . . . . . . . . . . . . . . . . . . . . . . . . . . . . . . . . . . . . . . . . . . . . . . . . . . . . . . . . . . . . 5

- Hyposphaenium-2 small . . . . . . . . . . . . . . . . . . . . . . . . . . . . . . . . . . . . . . . . . . . . . . . . . . . . . . . . . . . . . . . . . . . . . . . . . . . 6

- Hyposphaenium-2 absent . . . . . . . . . . . . . . . . . . . . . . . . . . . . . . . . . . . . .*P. arctophylax* (Norman and Stebbing, 1886) N Atlantic (David Strait, Iceland and Rockall basins, Bay of Biscay); 1970–4720 m

- Cheliped carpus slender (1.4 L:W) . . . . . . . . . . . . . . . . . . . . . . . . . . . . . . . . . . . . . . . . . . . . . . . . . *P. corroboree* sp. nov.

SE Australia (Bass Strait); 1450–1975 m

- Cheliped carpus robust (1.1 L:W) . . . . . . . . . . . . . . . . . . .. . . . . . . . . . . . . . . . . . . . . . . . . . . . . . . .*P. moonwalk* sp. nov.

NW Atlantic (Gay Head–Bermuda transect); 2178 m

- Pereopods 4–6 propodus distodorsal spine strongly serrate . . . . . . . . . . . . . . . . . . . . . . . . . . . . . *P. rocknroll* sp. nov.

NW Atlantic (Gay Head–Bermuda transect) 2440–2707 m

- Pereopods 4–6 propodus distodorsal spine finely serrate . . . . . . . . . . . . . . . . . . . . . . . . . . . . . . . . . . *P. tango* sp. nov.

SW Atlantic (Argentine Basin); 2440–2707 m

- Pleonites lateral margin pointed . . . . . . . . . . . . . . . . . . . . . . . . . . . . . . . . . . . . . . . . . . . . . . . . . . . . .*P. polonez* sp. nov.

NE Pacific (Clarion Clipperton Zone); 4365–4823 m

- Pleonites lateral margin smooth . . . . . . . . . . . . . . . . . . . . . . . . . . . . . . . . . . . . . . . . . . . . . . . . . . . . . . . . . . . . . . . . . . . . . 9

- Pereopods 4–6 with distodorsal spine strongly serrate . . . . . . . . . . . . . . . . . . . . . . . . . . . *P. dissimilis* (Lang,1972)

NW Atlantic (Sargasso Sea); 6000 m

- Pereopods 4–6 with distodorsal spine weakly serrate . . . . . . . . . . . . . . . . . . .. . . . . . . . . . . . . . . . . . . . . . . . . . . . . . . .10

- Pereonite 1 long (almost as long as wide). Pereonites 1–3 merus with robust and fine spines . . . . . . . . . . . . . . . . . . . . . . . . . . . . . . . . . . . . . . . . . . . . . . . . . . . . . . . . . . . . . . . . . . . . . . . . . . . . . . . . . . . . . . . . . . . . . . . . . . . . . . . . . . . . . .. . . . 11

- Pereonite 1 short (clearly shorter than wide). Pereonites 1–3 merus with fine spines only . . . . . . . . . . . . . . . . . . . . . . . . . . . . . . . . . . . . . . . . . . . . . . . . . . . . . . . . . . . . . . . . . . . . . . . . .*P. spinimaxillipeda* (Larsen and Araújo-Silva, 2014)

NE Pacific (Clarion Clipperton Zone); 4259–4261 m

- Pereopods 1–3 merus with robust spines, maxilliped palp article-3 with simple long seta . . . . . . . . . . . . . . . . . . . . . . . . . . . . . . . . . . . . . . . . . . . . . . . . . . . . . . . . . . . . . . . . . . . . . . . . . . . . . . . . . . . . . . . . . . . . . . . . . . . . . *P. kizomba* sp. nov.

N Atlantic (Vema Fracture Zone); 5127–5137 m

- Pereopods 1–3 merus fine spine, maxilliped palp article-3 with short setae only . . . . . . . . . . . . . . . . . . . . . . . . . . . . . . . . . . . . . . . . . . . . . . . . . . . . . . . . . . . . . . . . . . . . . . . . . . . . . . . . . . . . . . . . . . . . . . . . . . . . . . . . . . . . . . . . *P. samba* sp. nov.

SW Atlantic (N Brazilian Basin); 3459 m

### Genus *Armatognathia* Kudinova-Pasternak, 1987

*Armatognathia* Kudinova-Pasternak 1987: 28, 30–31, 36^17^; Gutu and Sieg 1999: 384^84^; Larsen and Wilson, 2002: 217, 218, Table 1 ^15^; Larsen 2005: 242^85^; Bird and Larsen 2009: Table 5 ^48^; Jóźwiak, Stępień and Błażewicz-Paszkowycz 2009: 56, 58^16^; Larsen, Gutu and Sieg 2015: 304^77^.

*Armathognathia* [sic] Larsen and Wilson, 2002: Table 1 ^15^.

*Arthrura* Kudinova-Pasternak 1973, in part: 162–164^86^; Sieg 1986: 168^73^; Bird and Holdich 1989: 138^19^; Jóźwiak and Błażewicz-Paszkowycz 2011: 47^87^.

Type species: *Armatognathia birsteini* Kudinova-Pasternak, 1987

Species included: *Armatognathia birsteini* Kudinova-Pasternak, 1987; *A. milonga* sp. nov.; *A. shiinoi* (Kudinova-Pasternak, 1973); *A. swing* sp. nov.

### Diagnosis

Female. Body elongate (8–10 L:W). Antenna article-2 with dorsodistal spine. Molar with spines. Maxilliped endites with round gustatory cusps. Cheliped basis posterior part long (dorsal concave in midlength). Cheliped carpus short (1.0–1.1 L:W). Chela robust (usually <1.3 L:W). Pleopods present. Uropod endopod article-1 longer than article-2.

***Armatognathia birsteini*** **Kudinova-Pasternak, 1987**

### Diagnosis

Carapace long, 2.0 L:W. Pereonite-1 0.7 L:W, pereonites 2–5 longer than wide. Antenna article-4 2.4x article-5. Mandible molar with one strong and two weak setae. Pereopod-1 merus with one spine, carpus with short spines only. Pereopod-6 carpus with four spines.

### Remarks

From all *Armatognathia* species *A. birsteini* has the most elongate carapace that is 2.0 L:W. Also it is distinguished from *A shiinoi* by absence of a long spine on the pereopod-1 carpus present in *A. shiinoi*^17^.

### Distribution

The species is known from the type locality in the Madagascar Basin of the Indian Ocean from 3923 to 4365 m depth.

***Armatognathia milonga*** **Błażewicz and Jóźwiak sp. nov.**

Figures 40 and 41

**Figure 40:**
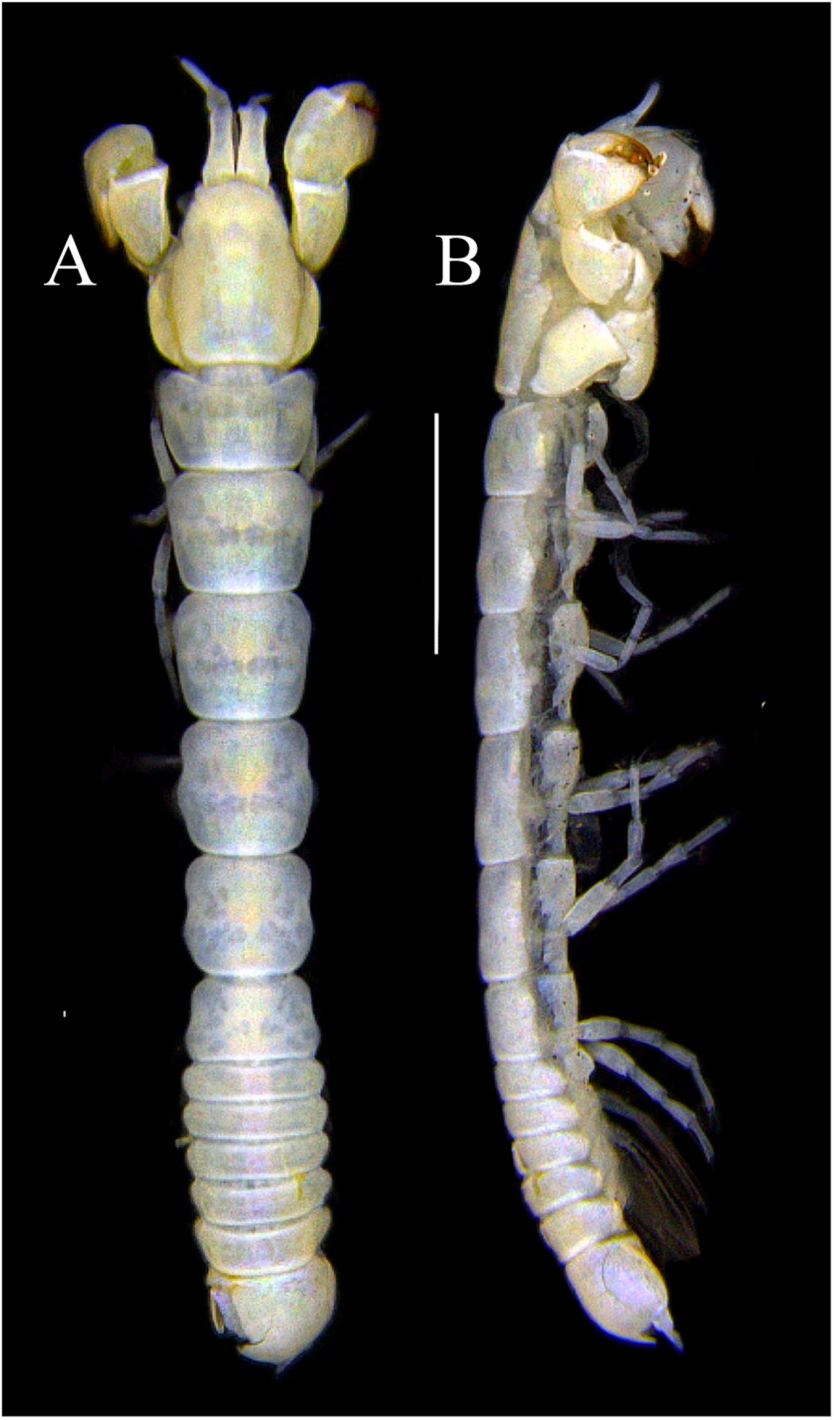
*Armatognathia milonga* sp. nov., holotype, ovigerous female (ZMH K-56004) (**A,B**) dorsal and lateral view, respectively. Scale 1mm.

**Figure 41:**
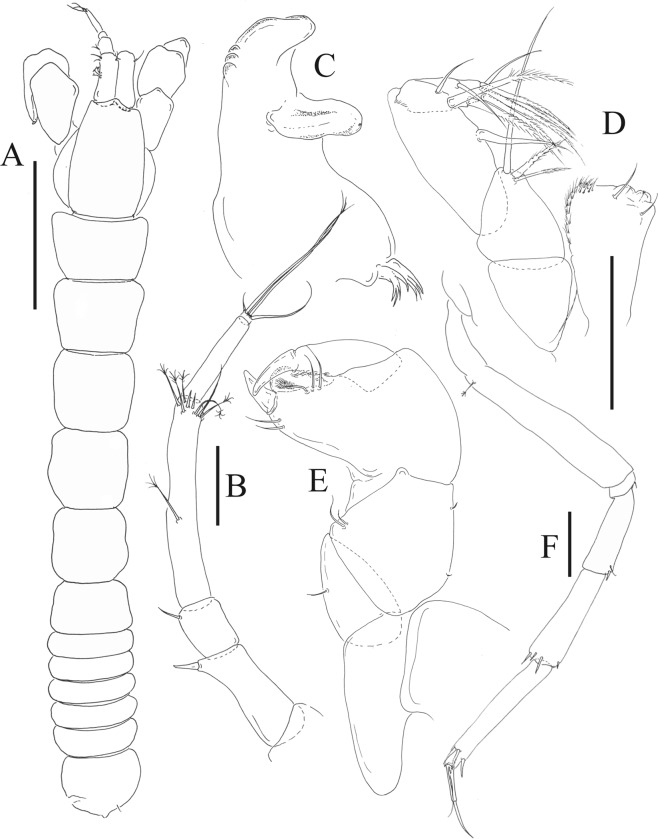
*Armatognathia milonga* sp. nov., holotype (ZMH K-56004) (**A**) Female, dorsal view. (**B**) Antenna. (**C**) Mandible left. (**D**) Maxilliped. (**E)** Cheliped. (**F**) Pereopod-1. Scale 0.1 mm.

### Material examined

Holotype, ovigerous female (4.6 mm BL), (ZMH K-56004), RV *Meteor*, DIVA3, St. 533.

### Diagnosis

#### Female

Carapace long, 1.7 L:W. Pereonite-1 0.7 L:W, pereonites 2–5 subsquare; pereonites 1–3 weakly wider proximally; pereonites 3–5 lateral almost parallel. Antenna article-4 2.1x article-5. Mandible molar with two serrate spines. Pereopod-1 merus with small spine and seta. Pereopods 4–6 carpus with four spines.

### Etymology

The name is after milonga, the music and dance originated in Argentina. Noun in apposition.

**Description of neuter**, length 3.6 mm. Body (Fig. 41A) slender 9.1 L:W; cephalothorax 1.7 L:W, 2.0x pereonite-1. Pereonites 1−6: 0.7, 0.9, 1.0, 1.0, 1.0 and 0.7 L:W, respectively. Pleon 0.25 of total body length. All pleonites the same size, 0.3 L:W. Pleotelson 2.5x pleonite-5. Pleotelson rounded, apex small, directed downward.

Antenna (Fig. 41B) article-1 fused with body; article-2 3.0 L:W, 1.7x article-3, with distal spine; article-3 1.4 L:W, 0.25x article-4, with distal seta; article-4 6.9 L:W, 2.0x article-5, with three short, one long simple, five penicillate setae distally and penicillate midlength seta; article-5 4.0 L:W, naked; article-6 as long as wide, with three long and two short distal setae.

Mouthparts. Left mandible (Fig. 41C) incisor distally blunt, margin weakly crenulate; *lacinia mobilis* large, with simple edge; molar distally rounded, with two distally multifurcate spines. Maxilliped (Fig. 41D) basis not observed; palp article-1 0.9 L:W, naked; article-2 1.1 L:W, with three serrate inner setae (one very long); article-3 1.9 L:W, with three inner setae (fourth seta not seen); article-4 2.5 L:W, with five distal and subdistal inner, sparsely serrate setae and outer seta; maxilliped endites separate, 2.2 L:W, distally with two round gustatory cusps and two distal setae.

Cheliped (Fig. 41E) sclerite large semitriangular; basis 2.8 L:W, subdistal dorsal seta not seen; merus wedge-shape, with ventral seta; carpus 1.2 L:W, with two ventral setae, one subproximal and one subdistal setae dorsally; chela robust 1.3x carpus, 1.5 L:W; propodus (palm) seta near dactylus insertion not seen; fixed finger with robust distal spine, with three setae on cutting margin and two ventral setae; cutting margin robust and well calcified; fixed finger and dactylus unifacial; dactylus with spines on cutting margin, subproximal seta on inner side not seen, robust unguis bent downward.

Pereopod-1 (Fig. 41F) basis 7.8 L:W, with subproximal penicillate seta on dorsal margin; ischium with seta; merus 3.2 L:W, 0.9x carpus, with small spine and seta; carpus 3.9 L:W, 0.8x propodus, with four small distal spines; propodus 7.0 L:W, 2.8x dactylus, with one ventrodistal and two dorsodistal serrate spines; dactylus 9.0 L:W, 1.1x unguis, proximal seta reaching 0.3 unguis length; unguis and dactylus about 0.6x propodus.

### Distribution

The species is known only from the type locality in the Argentine Basin at 4601‒4607 m depth.

### Remarks

*Armatognathia milonga* sp. nov. can be distinguished from all congeners by the presence of a small spine and seta on pereopod-1 merus, where all other species have one (often robust) spine only.

***Armatognathia shiinoi*** **(Kudinova-Pasternak, 1973)**

*Arthrura shiinoi:* Kudinova-Pasternak, 1973: 162‒164, Fig. 13 ^86^.

### Diagnosis

Carapace long, 1.5 L:W. Pereonite-1 0.8 L:W, pereonites 2–5 subrectangular, pereonites 1–3 weakly wider proximally; pereonites 4–5 wider in midlength. Antenna article-4 3.5 x article-5. Mandible molar with four spines. Pereopod-1 merus with robust spine, carpus with long distodorsal spine. Pereopods 4–6 carpus with four spines.

### Remarks

Presence of a long distodorsal spine on the pereopod-1 carpus is a unique character that distinguishes *Armatognathia shiinoi* from all other member of the genus.

### Distribution

The species is known only from the Gulf of Alaska at 3450‒3460 m depth.

***Armatognathia swing*** **Błażewicz and Jóźwiak sp. nov.**

Figures 42–48

**Figure 42:**
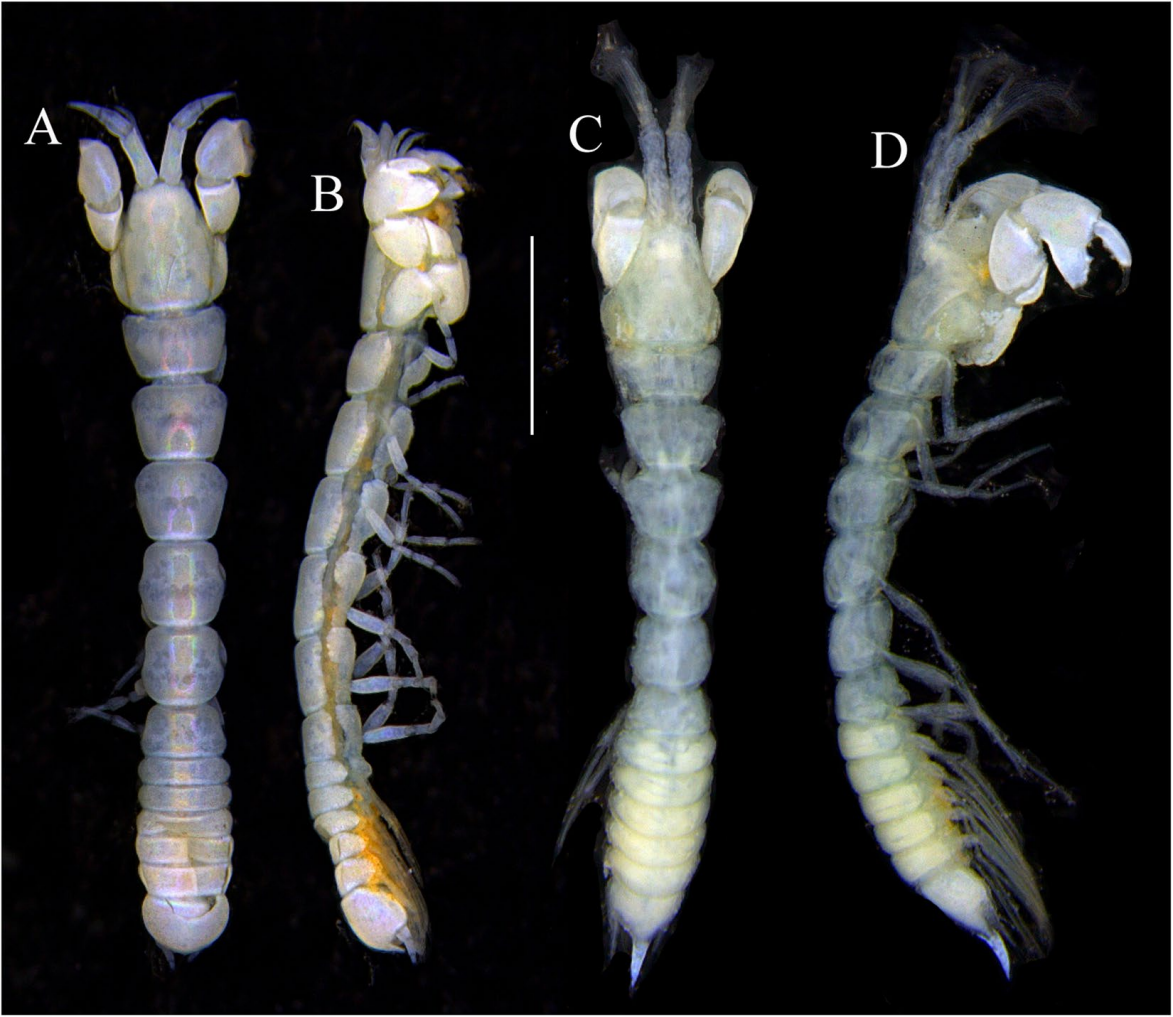
*Armatognathia swing* sp. nov. Holotype, female (MCZ:IZ:48509). (**A**) dorsal, (**B**) lateral, respectively Paratype male (MCZ:IZ:149573) (**C**) dorsal, (**D**) lateral. Scale 1 mm.

**Figure 43:**
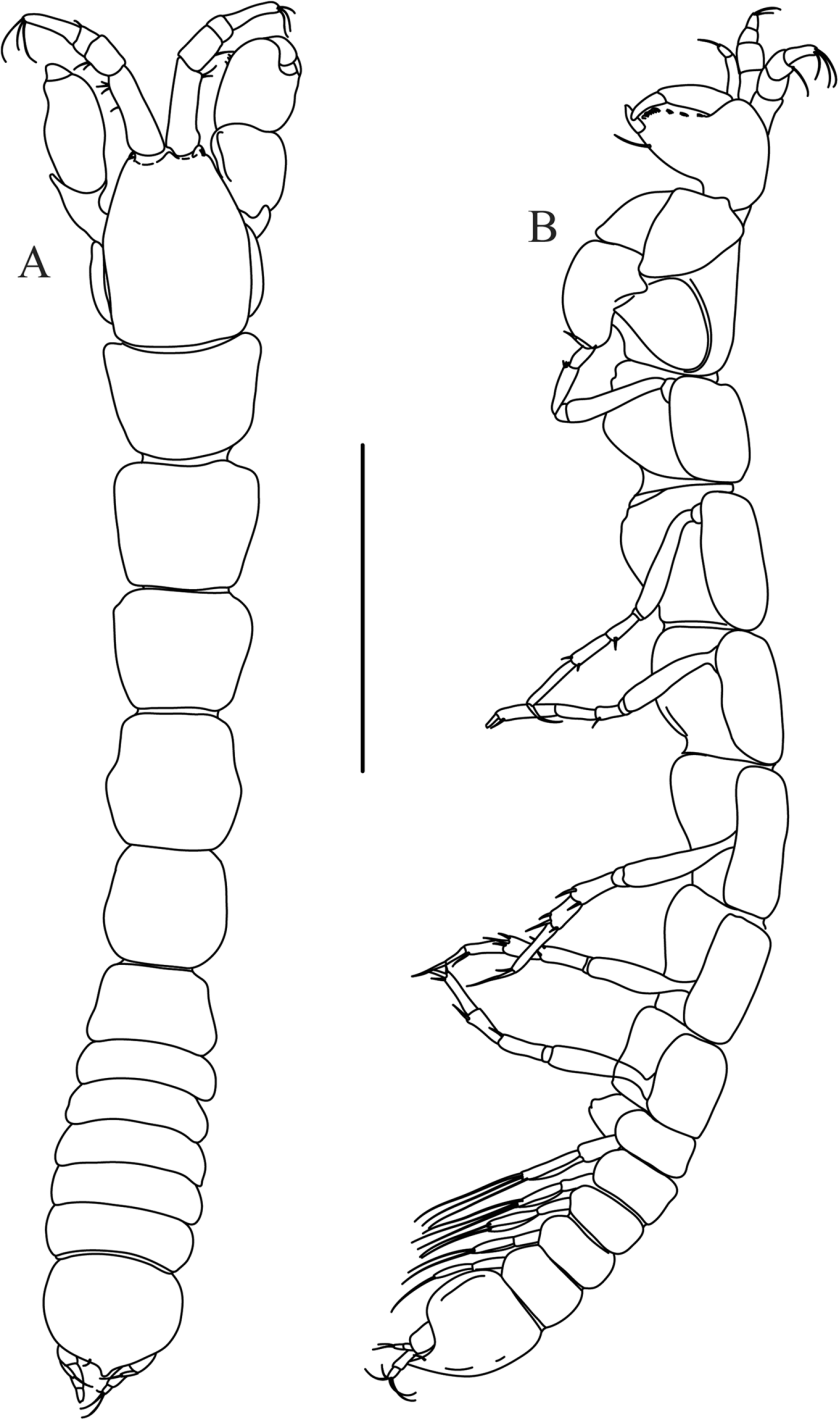
*Armatognathia swing* sp. nov., Holotype, female (MCZ:IZ:48509) (**A**) Dorsal. (**B**) Lateral. Scale 1 mm.

**Figure 44:**
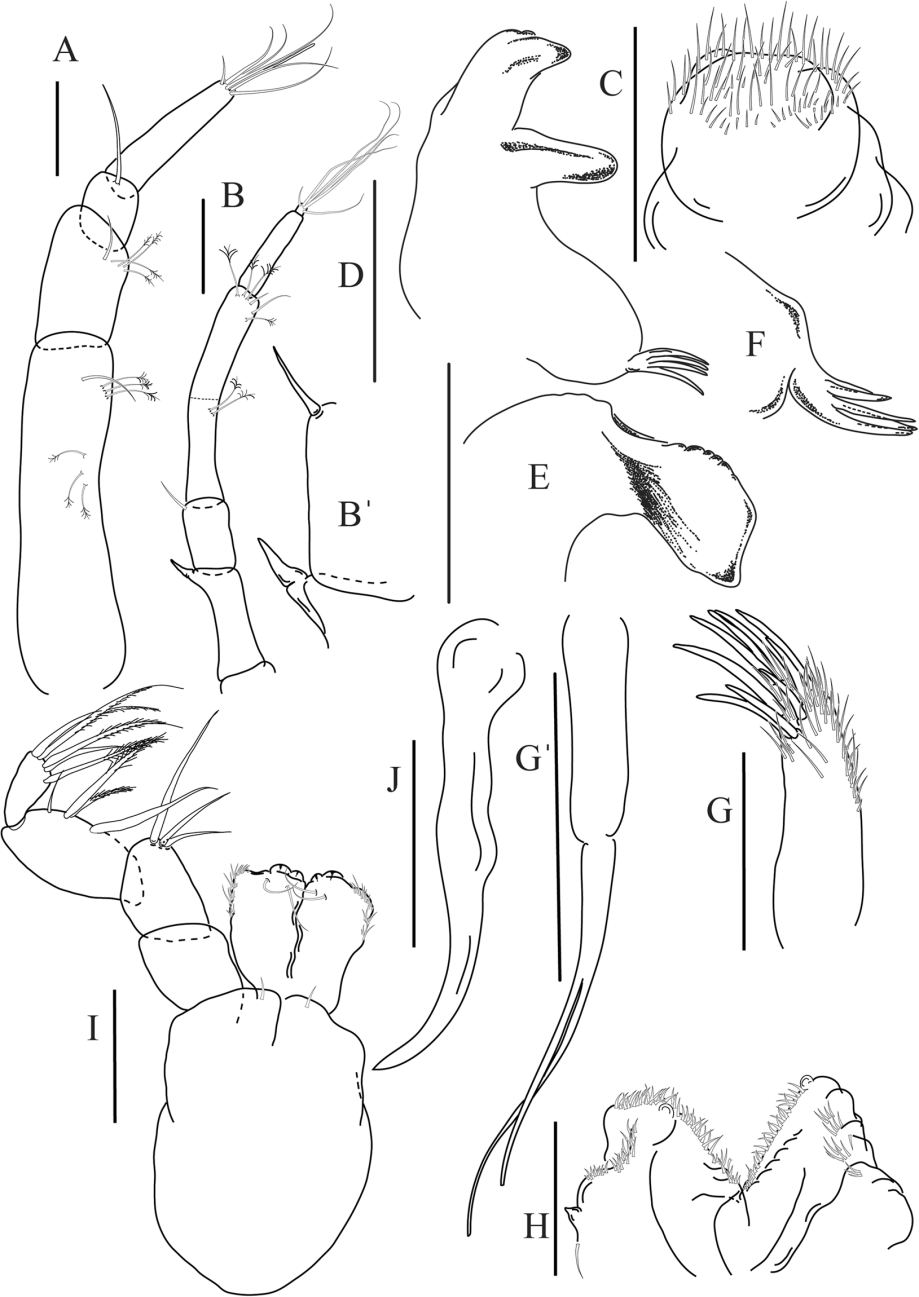
*Armatognathia swing* sp. nov., paratype, female (MCZ:IZ:149575) (**A**) Antennule. (**B**) Antenna. (**B’**) Antennule article 2–3 details. (**C**) Labrum. (**D**) Mandible, left. (**E**) Mandible right, incisor. (**F**) Mandible right molar, details. (**G**) Maxillule, endite. (**G’**) Maxillule palp. (**H**) Labium. (**I**) Maxilliped. (**J**) Epignath. Scale 0.1 mm.

**Figure 45:**
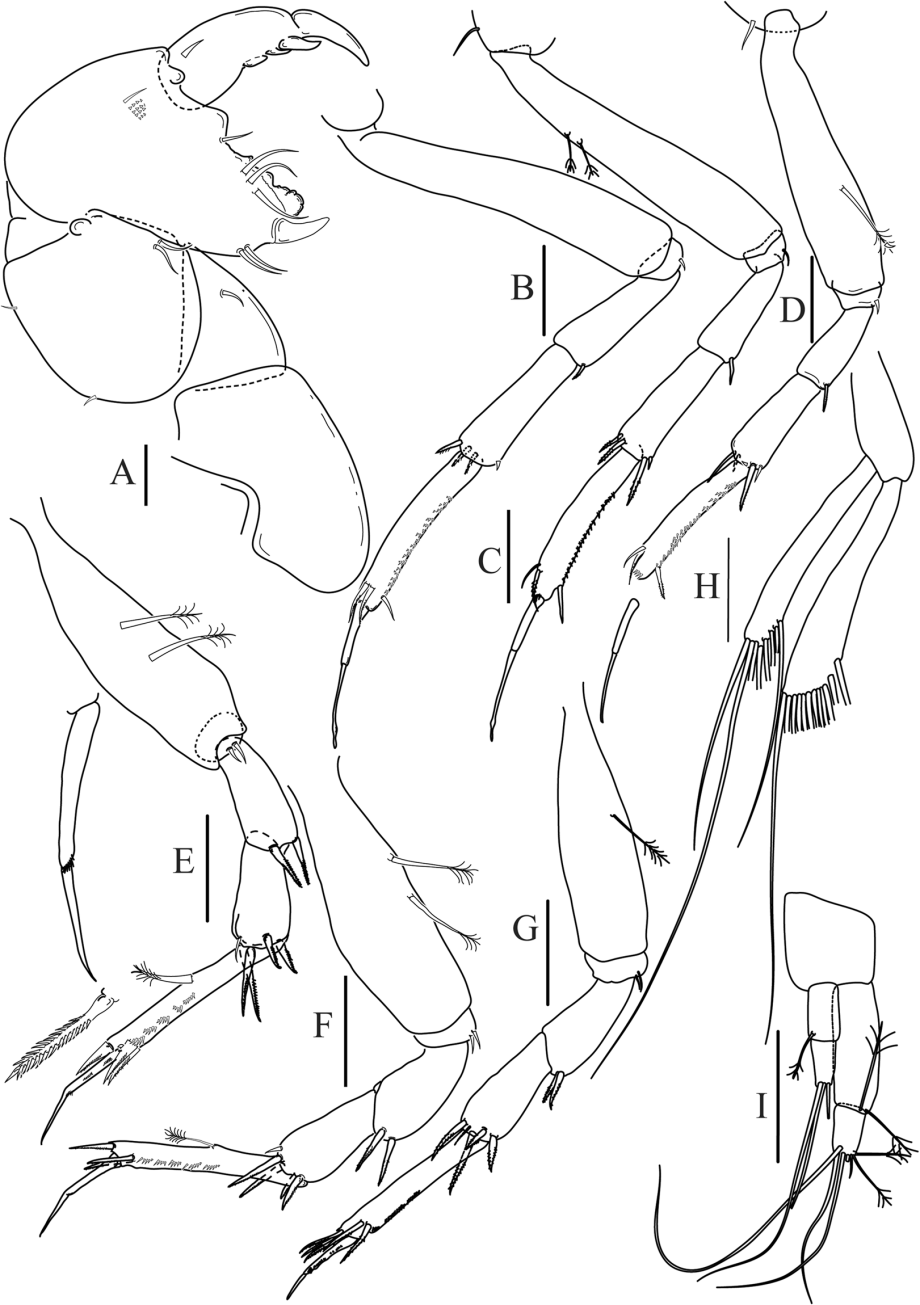
*Armatognathia swing* sp. nov. Paratype, female (MCZ:IZ:149575) (**A**) Cheliped. (**B**–**G**) Pereopod 1–6, respectively. (**H**) Pleopod. (**I**) Uropod. Scale 0.1 mm.

**Figure 46:**
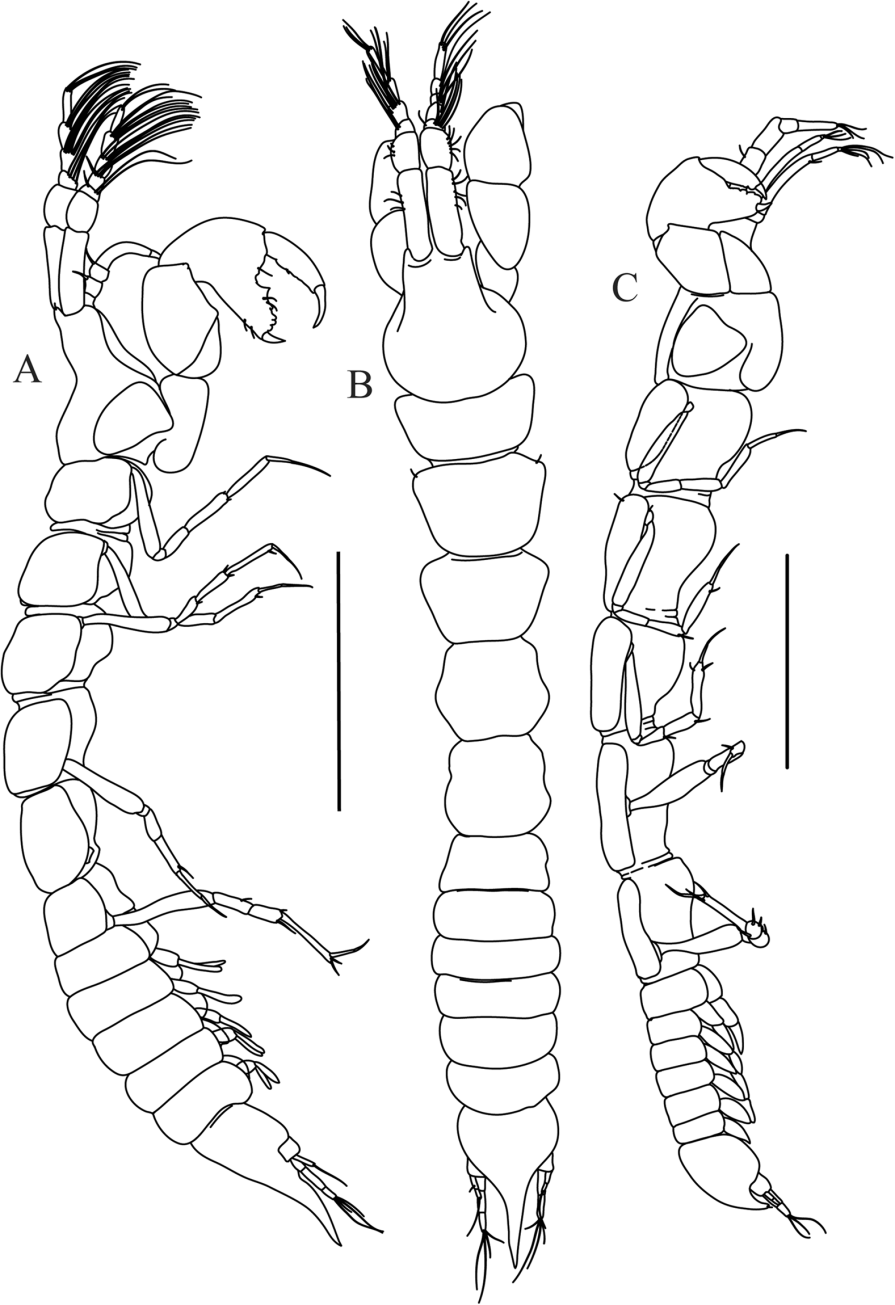
*Armatognathia swing* sp. nov. Paratype, male (MCZ:IZ:149573) (**A**) lateral, (**B**) dorsal. Paratype manca-3 (MCZ:IZ:48541) (**C**) lateral. Scale 1 mm.

**Figure 47:**
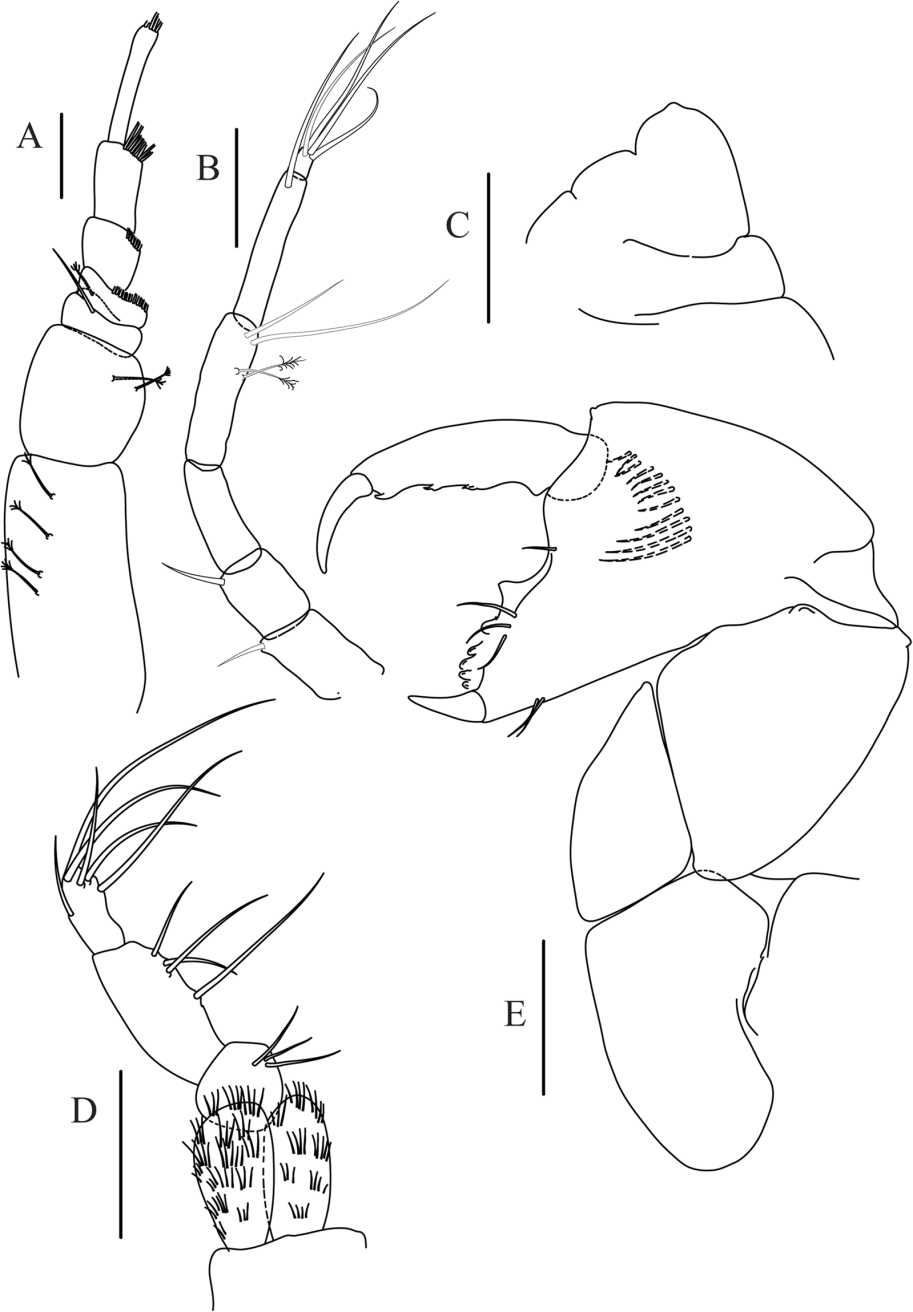
*Armatognathia swing* sp. nov., paratype, male (MCZ:IZ:149575) (**A**) Antennule. (**B**) Antenna. (**C**) Labrum. (**D**) Maxilliped. (**E**) Cheliped. Scale 0.1 mm.

**Figure 48:**
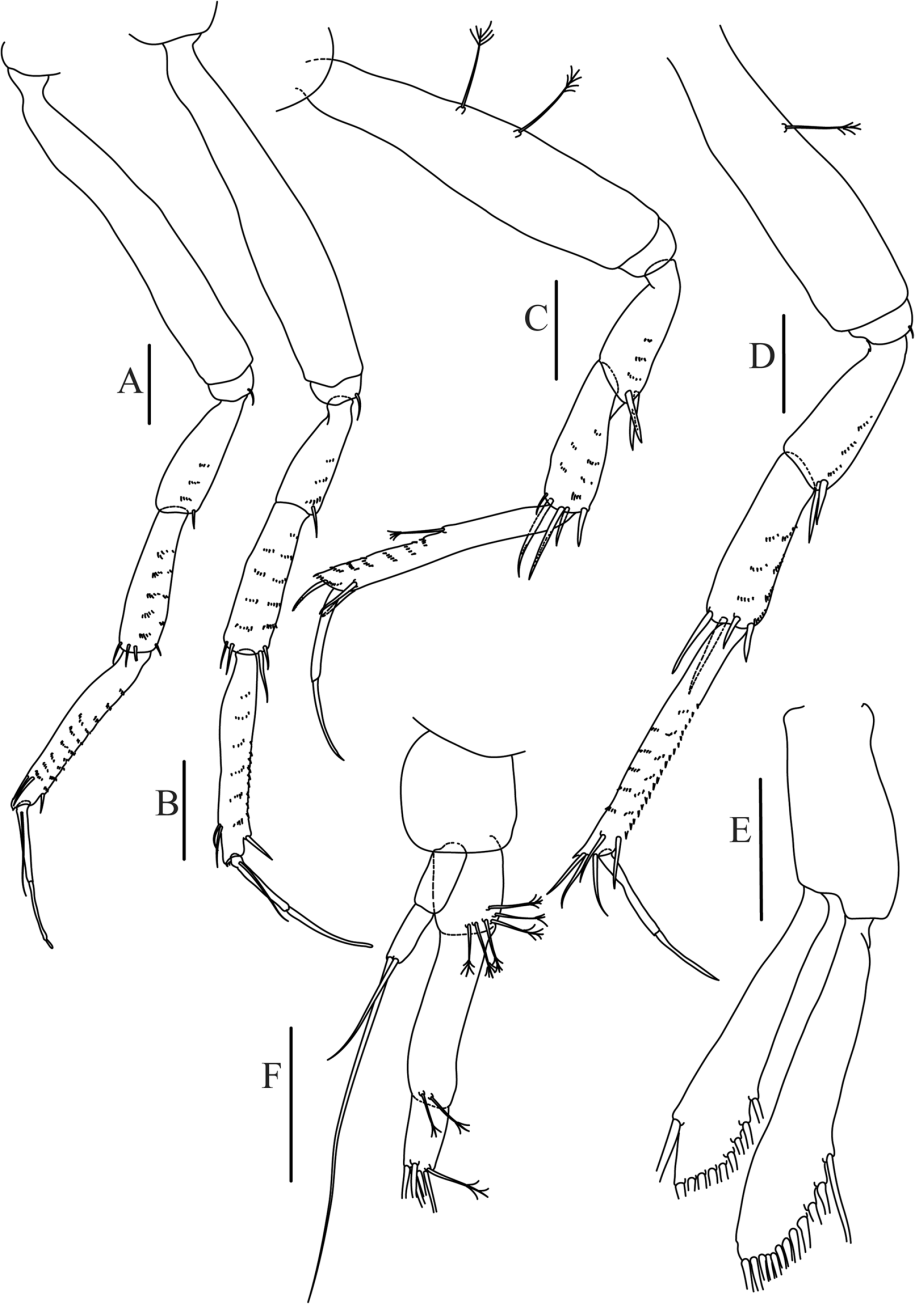
*Armatognathia swing* sp. nov., paratype, male (MCZ:IZ:149575) (**A**–**D**) Pereopod 1, 2, 4 and 6, respectively. (**E**) Pleopod. (**F**) Uropod. Scale 0.1 mm.

### Material examined

Holotype, neuter (4.0 mm BL), (MCZ:IZ:48509) Gay Head-Bermuda transect, RV *Atlantis II*, Cr. 24, St. 122.

### Paratypes

male (4.0 mm BL) (MCZ:IZ:149573); four neuters (3.5–3.63 mm BL), two mancas-2 (2.3–3.5 mm BL), (MCZ:IZ:149574) (Fig. 42C); female (dissected), (MCZ:IZ:149575), male (dissected), (MCZ:IZ:149583), the same locality as holotype; manca-3 (2.8 mm BL) (MCZ:IZ:48515), RV *Chain*, Cr. 58, St. 100; manca-3 (3.0 mm BL), (MCZ:IZ:48541), RV *Atlantis II*, Cr 24. St. 121.

### Diagnosis

#### Female

Carapace short, 1.3 L:W. Pereonite-1 0.7 L:W, pereonites 2–5 subrectangular, pereonites 1–3 weakly wider proximally; pereonites 4–5 margins rounded. Antenna article-4 2.5x article-5. Mandible molar with one process divided into four or five spines. Pereopod-1 merus with one robust spine; carpus with short spines only. Pereopods 4–6 carpus with four spines.

### Etymology

The species collected off the coast of the North America is named after a form of popular music developed in the United States. Noun in apposition.

**Description of neuter**, length 3.6 mm. Body (Fig. 43A,B) elongate, 9.7 L:W; cephalothorax 1.4 L:W, 1.8x pereonite-1. Pereonites 1−6: 0.7, 0.9, 0.9, 1.0, 1.0 and 0.6 L:W, respectively. Pleon 0.25 of total body length. All pleonites the same size, 0.3 L:W. Pleotelson 1.9x pleonite-5. Pleotelson rounded, apex small, directed downward.

Antennule (Fig. 44A) article-1 4.4 L:W, 2.5x article-2, with three middle penicillate setae and one simple and three penicillate distal setae; article-2 1.8 L:W, 2.0x article-3, with one simple and four penicillate setae arranged in transverse row distally; article-3 0.9 L:W, 0.5x article-4, with long distal seta; article-4 4.3 L:W; article-5 semifused, with article-4, with five long and one short setae, and one aesthetasc distally.

Antenna (Fig. 44B) 0.7 times length of antennule; article-1 fused with body; article-2 3.2 L:W, 1.3x article-3, with dorsodistal spine (Fig. 40B’); article-3 1.7 L:W, 0.4x article-4, with dorsodistal seta; article-4 7.9 L:W, 2.4x article-5, with two penicillate midlength setae and two simple and four penicillate setae distally; article-5 4.0 L:W, naked; article-6 as long as wide, with four long and two short distal setae.

Mouthparts. Labrum (Fig. 44C) distally obtuse with relatively sparse and robust distal setae. Right mandible (Fig. 44E) incisor distally blunt and simple, margin crenulate; molar not observed. Left mandible (Fig. 44D) incisor distally simple; *lacinia mobilis* well developed, with simple edge; molar distally rounded, with five distal spines. Maxillule endite (Fig. 44G) with nine strong distal spines of various length distally and numerous setae along outer margin; palp (Fig. 44G’) with two setae (broken). Maxilla not observed. Labium (Fig. 44H) with two lobes; inner lobe with spines on inner and distal margins; outer lobe smaller than inner lobe, with robust setae on the margin and pointed projection on lateral margin. Maxilliped (Fig. 44I) basis 1.4 L:W, with short distal seta; palp article-1 0.9 L:W, naked; article-2 1.1 L:W, with three serrate inner setae, outer seta not seen; article-3 1.9 L:W, with four inner setae (two robust sparsely serrate, two short serrate); article-4 2.5 L:W, with five distal and subdistal inner, sparsely serrate setae, outer seta not seen; maxilliped endites separate, 2.2 L:W, distally with two round gustatory cusps and two distal setae. Epignath (Fig. 41J) elongate, slender, distally pointed.

Cheliped (Fig. 45A) sclerite large semitriangular; basis 2.6 L:W, subdistal dorsal seta not seen; merus wedge-shape, with ventral seta; carpus 1.2 L:W, with two ventral setae, one subproximal and one subdistal setae dorsally; chela robust 1.4x carpus, 1.6 L:W; propodus (palm) with one seta near dactylus insertion and five setae on inner side (one robust, four short); fixed finger with robust distal spine (unguis) and two ventral setae; cutting margin with triangular tooth proximally, distally well calcified, with three unequal teeth and with three setae; dactylus and fixed finger unifacial; dactylus bent downward, with three robust teeth on cutting margin and subproximal seta on inner side; unguis robust.

Pereopod-1 (Fig. 45B) longer than pereopods 2–3; coxa present, no seta observed; basis 6.5 L:W, naked; ischium with seta; merus 3.2 L:W, 0.9x carpus, with serrate distal spine; carpus 3.7 L:W, 0.8x propodus, with three serrate and one simple distal spines; propodus 6.4 L:W, 2.7x dactylus, with one ventrodistal and two dorsodistal serrate spine; dactylus 9.0 L:W, 0.7x unguis, proximal seta not seen; unguis and dactylus about 0.8x propodus.

Pereopod-2 (Fig. 45C) coxa with one seta; basis 5.4 L:W, with two dorsal penicillate setae; ischium with ventral seta; merus 2.7 L:W, 0.8x carpus, with distal spine; carpus 3.5 L:W, 0.7x propodus, with four serrate distal spines (three long, one short); propodus 6.2 L:W and 3.0x dactylus, with subdistal ventral serrate spine, fine and serrate spine dorsodistally, numerous microtrichia ventrally; dactylus 7.1 L:W, 0.7x unguis; dactylus and unguis together 0.8x propodus.

Pereopod-3 (Fig. 45D) similar as pereopod-2, but basis with one penicillate seta.

Pereopod-4 (Fig. 45E) basis 2.9 L:W, with two penicillate setae on ventral margin; ischium with two ventral setae; merus 1.7 L:W, as long as carpus, with two ventrodistal serrate spines; carpus 2.0 L:W, 0.7x propodus, with four serrate spines and one seta distally; propodus 8.0 L:W, with two ventrodistal serrate spines and one strongly serrate dorsodistal spine; dactylus 12.5 L:W; unguis 0.9x dactylus; together 0.85x propodus.

Pereopod-5 (Fig. 45F) as pereopod-4.

Pereopod-6 (Fig. 45G) similar to pereopod-5, but propodus with three spines dorsodistally.

Uropod (Fig. 45I) basal article 1.4 L:W; exopod with two articles, 0.6x endopod, article-1 1.8 L:W, with distal penicillate seta, article-2 2.6 L:W, with two distal setae; endopod with two articles, article-1 2.0 L:W, 2.5x article-1,with two penicillate distal setae; article-2 1.6 L:W, with five simple and two penicillate setae distally.

**Description of adult male**, length 3.4 mm. Body (Fig. 46A,B) slender, 8.5 L:W; cephalothorax as long as wide, 2.5x pereonite-1. Pereonites 1−6: 0.5, 0.8, 0.7, 0.8, 0.8 and 0.5 L:W, respectively; pereonites 1–3 wider proximally, pereonites 4 and 5 wider medially, pereonite-6 wider distally; pleonites lateral side rounded, without hyposphaenium. Pleon 0,2 of total body length. All pleonites the same size, 0.4 L:W, with rounded lateral margins. Pleotelson 3.2x pleonite-5, apex 2.0x pleotelson (proximal part).

Antennule (Fig. 47A) article-1 2.3 L:W, 2.1x article-2, with four distal and subdistal penicillate setae; article-2 1.2 L:W, 3.9x article-3, with two penicillate setae distally; article-3 0.4 L:W, as long as article-4, with two distal setae; article-4 0.2 L:W, 0.3x article-5, with numerous aesthetascs arranged in transversal row; article-5 0.8 L:W, 0.5x article-6, with numerous long aesthetascs arranged in transversal row; article-6 2.4 L:W. 0.8x article-7, with a few aesthetascs distally; article 7 4.0 L:W, with five setae distally.

Antenna (Fig. 47B) 0.6 times length of antennule; article-1 fused with body; article-2 1.4 L:W, 0.9x article-3, with distal seta; article-3 1.4 L:W, 0.6x article-4, with distal seta; article-4 3.6 L:W, 0.9x article-5 naked; article-5 7.0 L:W, with two penicillate and two simple setae distally; article-6 6.0 L:W, with distal seta; article-7 as long as wide, with at least four distal setae.

Mouthparts. Labrum (Fig. 47C) reduced to naked process, with transversal ridges. Mandible, maxillule, maxilla and labium reduced to small plates (not illustrated). Maxilliped (Fig. 47D) palp article-1 1.2 L:W, naked; article-2 1.3 L:W, with three inner setae; article-3 1.7 L:W, with four long inner setae; article-4 2.9 L:W, with five distal and subdistal inner setae; one outer seta; maxilliped endites separated, narrow with numerous microtrichia. Epignath not seen.

Cheliped (Fig. 47E) basis 2.1 L:W; merus wedge-shape, ventral seta not seen; carpus little wider medially, 1.5 L:W, setae not seen; chela 1.2x carpus, 1.4 L:W; propodus (palm) with 12 setae on inner side; fixed finger with calcified inner margin (distally four to five tubercles, proximally triangular tooth), with three setae on cutting edge and two ventral setae; dactylus 5.3 L:W, distally bent downward, with robust unguis.

Pereopod-1 (Fig. 48A) basis 7.2 L:W, naked; ischium with seta; merus 2.6 L:W, 0.7x carpus, with ventrodistal small spine; carpus 4.2 L:W, 0.8x propodus, with four small distal spine; propodus 8.0 L:W, 2.5x dactylus, with two unequal subdistal spines dorsally and one ventrodistal spine; dactylus 9.0 L:W, with dorsoproximal seta little longer than dactylus; unguis about as long as dactylus.

Pereopods 2–3 (Fig. 48B) similar to pereopod-1; unguis 1.5x dactylus.

Pereopod-4 (Fig. 48C) basis 5.6 L:W, with two penicillate ventral setae; ischium seta not observed; merus 3.1 L:W, as long as carpus, with two ventrodistal spines; carpus 3.7 L:W, 0.7x propodus, with four distal spines (two short and two long) and dorsodistal spine; propodus 9.5 L:W, with two ventrodistal spines and dorsodistal spine; dactylus 10.6 L:W; unguis 1.1x dactylus; combined 0.8x propodus.

Pereopod-5 as pereopod-4.

Pereopod-6 (Fig. 48D) similar to pereopod-4, but ischium with one seta and propodus with three dorsodistal spines;

Pleopods 1−5 (Fig. 48E) basal article 2.0 L:W; exopod 0.8x endopod, 5.2 L:W, with nine distal and ventrodistal plumose setae and dorsal long plumose seta; endopod 5.5 L:W, with 11 distal and ventrodistal plumose setae.

Uropod (Fig. 48F) basal article 0.9 L:W; exopod with two articles, 0.3x endopod, article-1 2.1 L:W, naked, article-2 2.4 L:W, with two distal setae; endopod with three articles, article-1 1.2 L:W, with six penicillate setae; article-2 4.1 L:W, with two penicillate distal setae; article-3 2.0 L:W, with five simple and one penicillate distal setae.

### Distribution

The species is known from the Gay Head–Bermuda transect between 4743‒4892 m depth.

### Remarks

*Armatognathia swing* sp. nov. is the second representative of the genus *Armatognathia* described in the Atlantic Ocean. A third species inhabitant the NE Atlantic in both abyssal Porcupine and Bay of Biscay plains remains undescribed (Bird, pers. comm.). From *Armatognathia milonga*, the other western Atlantic species (see above), it can be distinguished by the presence of only one small spine on the pereopod-1 merus (small seta and small spine in *A. milonga*) and shorter carapace that is only 1.3 as long as wide (carapace is 1.7x L: W). Two other species *A. birsteini* (type species) and *A. shiinoi* occur in the Indian Ocean and North Pacific, respectively. The new species can be distinguished by: relatively long antenna article-5, that is 2.5 times as long as article-4 (3.5 times in *A. shiinoi*) and absence of long distodorsal spine on carpus of pereopod-1 (present on *A. shiinoi*) and relatively short carapace that is only 1.3x L:W (2.0x L:W in *A. birsteini*).

## Discussion

### Diversity

The new family Paranarthrurellidae is so far represented by 16 nominal species classified to two genera. It is a rather less diverse family that holds 14^th^ place on the list of 21 tanaidomorph families gathering 1.8% of all tanaidomorph species (Table 6). The two paranarthrurellid genera, *Paranarthrurella* and *Armatognathia*, which were removed from Paratanaoidea *incertae sedis* group, reduced the number of the taxa with uncertain family classification by seven species (<1%). Nevertheless, the group Paratanaoidea *incertae sedis* still contains 52 species and 29 genera (5.8% and 15.3%, respectively).

**Table 6:** Diversity and contribution of Paranarthrurellidae fam. nov. to the suborder Tanaidomorpha.

Family	Genera	Species	[%]
Leptocheliidae	30	126	14.1
Typhlotanaidae	13	109	12.2
Tanaididae	19	91	10.2
Paratanaidae	10	57	6.4
Colletteidae	16	56	6.3
Akanthophoreidae	9	53	5.9
**Paratanaoidea** ***incertae sedis***	**29**	**52**	**5.8**
Pseudotanaidae	3	51	5.7
Neotanaidae	4	51	5.7
Agathotanaidae	7	51	5.7
Tanaellidae	5	49	5.5
Leptognathiidae	1	34	3.8
Anarthruridae	16	29	3.3
Tanaopsidae	1	17	1.9
**Paranarthrurellidae**	**2**	**16**	**1.8**
Tanaissuidae	5	12	1.3
Nototanaidae	7	12	1.3
Cryptocopidae	6	11	1.2
Heterotanoididae	1	5	0.6
Mirandotanaidae	3	4	0.4
Teleotanaidae	1	3	0.3
Pseudozeuxidae	2	2	0.2
total:	190	891	

In case of the species analyzed by us, small but noticeable morphological differences distinguishing each species can be pointed out. In our material, most of them belonging to historical collections^2,88^, we were able to discriminate all the species based only on morphology according to phenotypic condition for species delimitation^89^. In many cases, morphological study is insufficient for identification of various groups of Peracarida crustaceans^90–93^ including tanaids^94^ (Jakiel *et al*. unpublished data).

The finding of previously undescribed species, as well as considerable cryptic diversity (i.e. high genetic divergence associated with high morphological similarity) is quite common in the vast and under-explored deep-sea^95–97^. Deep-sea peracarids are highly diverse^98^, and, in particular they appear to exhibit high degrees of cryptic and genetic diversity^99^ including amphipods^91^; isopods^92,93^; tanaidaceans^94,100^, Jakiel *et al*. (submitted). In the material examined herein, high levels of genetic diversity in 18S and COI similarly revealed cryptic species whose morphology is indeed different upon thorough re-examination (i.e. *Paranarthrurella* sp.1, *Paranarthrurella* sp.2).

### Phylogeny

Obtaining reliable quality DNA sequences for the deep-sea small crustaceans is a big challenge. The lack of a reasonable amount of genetic data is a main obstacle for comprehensive phylogenetic analysis that could confidently corroborate the relationship between higher tanaidacean taxa and shed light on origin and radiation place of deep-sea tanaidaceans. So far, the only phylogenetic approach implies, that colonising the deep sea by tanaids might have happened more than once in the Paratanaoidea^31^. Yet, the long branches suggest an ancient diversification of the two main clades: (1) Tanaidae + Neotanaidae and (2) Paratanaoidea with Leptocheliidae and Nototanaidae as ancestors. In our limited molecular approach, all taxa representing here the superfamily Paratanaoidea cluster together in a clade in consistence with the results of Kakui *et al*.^31^. The Paranarthrurellidae nest within the families showing the most distinctive apomorphic characters, rather than with the Leptocheliidae, a presumed plesiomorphic group.

### Distribution and bathymetry

**Figure 49:**
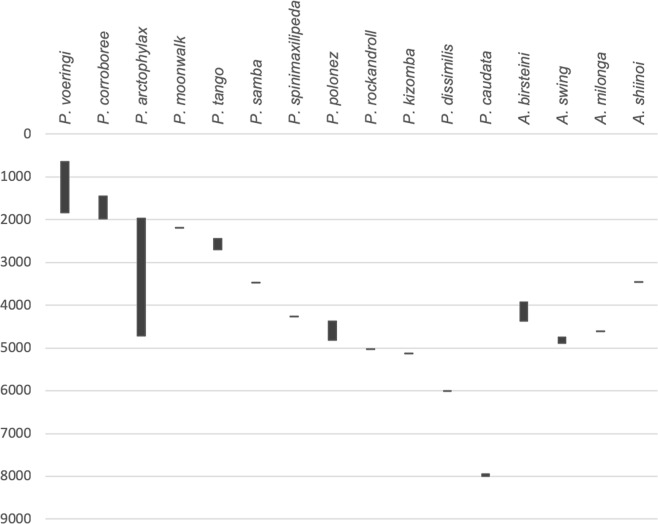
Bathymetric distribution of species for both *Paranarthrurella* and *Armatognathia* genera based on current study and literature data.

**Figure 50:**
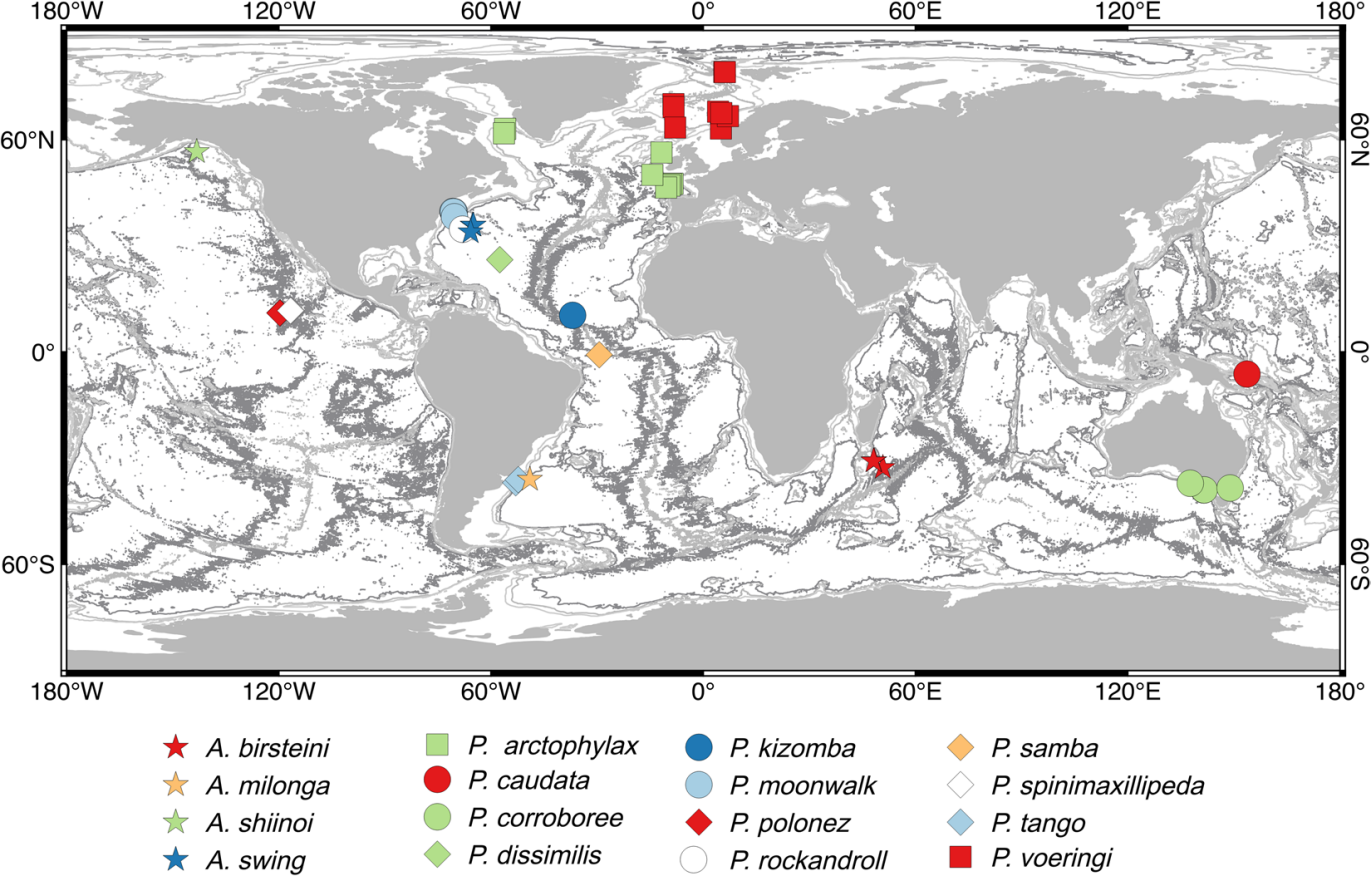
Worldwide distribution of the species of the Paranarthrurellidae fam.nov. based on the current study and literature data^2,9,12,16–18,86^.

The distribution of all taxa included in the Paranarthrurellidae indicates clearly the cosmopolitan and deep-water nature of the family (Figs 49 and 50). Tanaidacea are an abundant and diverse component of the benthic assemblages in all depths^100–102^ with 15 families of the superfamily Paratanoidea represented in the deep-sea: i.e. Agathotanaidae, Akanthophoreidae, Anarthruridae, Colletteidae, Cryptocopidae, Leptocheliidae, Leptognathiidae, Nototanaidae, Paratanidae, Pseudotanaidae, Pseudozeuxidae, Tanaellidae, Tanaopsidae, Tanaissuidae, and Typhlotanaidae. The Paranarthrurellidae, which is absent on the shelf, would be the sixteenth family within this group. Its occurrence in the deep-sea shows a distributional range from the bathyal to the hadal depths. The genus *Armatognathia* appears nearly exclusive to abyssal depths; whereas *Paranarthrurella*, reported into the hadal, is mainly distributed in the abyss, with an important occurrence in the bathyal (Fig. 49).

Originally described to accommodate one hadal species from the Bougainville Trench, *Paranarthrurella* holds the shallowest record for the family represented by *P. voeringi*, which is distributed in the upper bathyal of the Subarctic area of the North Atlantic, and *P. corroboree* occurring on the slope of the SE Australia. The absence of Paranarthrurellidae in shelf depths suggests that this family most probably radiated in the deep-sea, and all Paranarthrurellidae are blind species lacking eyelobes or even remnants thereof. Those lobes, which are occasionally observed in some deep-water representatives e.g. as *Protanais birsteini* Kudinova-Pasternak, 1970^103^ or *Bathytanaissus spinulosus* Bird and Holdich, 1989^104^, advocate for their shallow water ancestry.

*Paranarthrurella voeringi* shows the most widespread distribution of the species in the family. It has been collected at eight different stations, showing a distribution in the Atlantic Ocean from the 63ºN to the Arctic waters at the 79ºN (Table 5, Fig. 39). Furthermore, it exhibits a wide bathymetric range (1195 m). Such a large occurrence for a deep-sea tanaid, has been also noticed in this boreal area for *Pseudotanais affinis* Hansen, 1887, a putatively cryptic species with a quite similar zoogeography^94^. Within the same area in the Norwegian Basin, two *Paranarthrurella* species genetically distinguished (sp.1 and sp.2), look morphologically identical to *Paranarthrurella voeringi*. Both inhabit really cold temperatures (between −0.7 and −0.8 °C) fitting into the range that *P. voeringi* has been previously reported (between −0.4 and −1.4 °C)^6,8,61^ (references Table 5). The co-occurrence of both *Paranarthrurella* sp.1 and sp.2 at the same station shows how likely other records of *P. voeringi* could also correspond to distinct species from the genetical point of view but showing the same morphological features. Further molecular studies will be necessary to define the name of these species.

In the same way, *Paranarthrurella arctophylax* was described close to the Norwegian Basin in deeper but warmer area (2.8 °C)^105^, as Hansen noticed when he reported the species from the David Strait between 1.5 and 2.4 °C^8^ (Table 5). Later on, new records in the northern Bay of Biscay extended its bathymetrical distribution down to the abyssal, holding the widest depth range within the genus (2750 m); but always in warmer waters than the boreal neighbour *P. voeringi*^106,107^. If different water-mass conditions, including temperature, can drive the boundaries between both northern species (*P. arctophylax* and *P. voeringi*) to not overlap their geographical distribution, more records and genetical evidences would be necessary to prove the occurrence of *P. arctophylax* as a single species, especially in such a broad distance reported on both sides of the Mid-Atlantic Ridge.
